# Unconditional Cash Transfers and Maternal Assessments of Children's Health, Nutrition, and Sleep: A Randomized Clinical Trial

**DOI:** 10.1001/jamanetworkopen.2023.35237

**Published:** 2023-09-29

**Authors:** Jessica F. Sperber, Lisa A. Gennetian, Emma R. Hart, Alicia Kunin-Batson, Katherine Magnuson, Greg J. Duncan, Hirokazu Yoshikawa, Nathan A. Fox, Sarah Halpern-Meekin, Kimberly G. Noble

**Affiliations:** 1 Teachers College, Columbia University, New York, New York; 2 Duke University, Durham, North Carolina; 3 University of Minnesota, Minneapolis; 4 University of Wisconsin–Madison; 5 University of California, Irvine; 6 New York University, New York; 7 University of Maryland, College Park

## Abstract

**Importance:**

Children experiencing poverty are more likely to experience worse health outcomes, including injury, chronic illness, worse nutrition, and poorer sleep. The extent to which poverty reduction improves these outcomes is unknown.

**Objective:**

To evaluate the effect of a 3-year, monthly unconditional cash transfer on health, nutrition, sleep, and health care utilization among children experiencing poverty who were healthy at birth.

**Design, Setting, and Participants:**

This longitudinal randomized clinical trial recruited 1000 mother-infant dyads between May 2018 and June 2019. Dyads were recruited from postpartum wards in 12 hospitals in 4 US cities: New York, New York; Omaha, Nebraska; New Orleans, Louisiana; and Minneapolis/St Paul, Minnesota. Eligibility criteria included an annual income less than the federal poverty line, legal age for consent, English or Spanish speaking, residing in the state of recruitment, and an infant admitted to the well-baby nursery who will be discharged to the mother’s custody. Data analysis was conducted from July 2022 to August 2023.

**Intervention:**

Mothers were randomly assigned to receive either a high-cash gift ($333/mo, or $3996/y) or a low-cash gift ($20/mo, or $240/y) for the first several years of their child’s life.

**Main Outcomes and Measures:**

Primary preregistered outcomes reported here include an index of child health and medical care and child sleep disturbances. Secondary preregistered outcomes reported include children’s consumption of healthy and unhealthy foods.

**Results:**

A total of 1000 mother-infant dyads were enrolled, with 400 randomized to the high-cash gift group and 600 to the low-cash gift group. Participants were majority Black (42%) and Hispanic (41%); 857 mothers participated in all 3 waves of data collection. We found no statistically detectable differences between the high-cash and low-cash gift groups in maternal assessments of children’s health (effect size [ES] range, 0.01-0.08; SE range, 0.02-0.07), sleep (ES range, 0.01-0.10; SE, 0.07), or health care utilization (ES range, 0.01-0.11; SE range, 0.03-0.07). However, mothers in the high-cash gift group reported higher child consumption of fresh produce at child age 2 years, the only time point it was measured (ES, 0.17; SE, 0.07; *P* = .03).

**Conclusions and Relevance:**

In this study, unconditional cash transfers to mothers experiencing poverty did not improve reports of their child’s health, sleep, or health care utilization. However, stable income support of this magnitude improved toddlers’ consumption of fresh produce. Healthy newborns tend to grow into healthy toddlers, and the impacts of poverty reduction on children’s health and sleep may not be fully borne out until later in life.

**Trial Registration:**

ClinicalTrials.gov Identifier: NCT03593356

## Key Points

**Question:**

Does poverty reduction improve health, nutrition, and sleep in young children?

**Findings:**

In this randomized clinical trial of 1000 mother-child dyads experiencing poverty, a monthly unconditional cash transfer did not improve children’s health or sleep in the first 3 years of life. However, the cash transfers led to increased consumption of fresh produce at age 2 years.

**Meaning:**

Among children experiencing poverty, a monthly cash gift affected healthy food intake, but not health or sleep.

## Teaser

This randomized clinical trial evaluates the effect of monthly unconditional cash transfers on maternal assessments of health, nutrition, sleep, and health care utilization among US children experiencing poverty.

## Introduction

Children experiencing poverty are more likely to experience worse health outcomes, including injury,^1^ chronic illness,^2^ and poor sleep^3^ and are more likely to use emergency health services.^4^ Numerous factors likely contribute to these associations, including poor prenatal care,^5^ exposure to environmental toxicants,^6^ low quality housing and neighborhoods,^7^ and lack of access to medical care^7^ and nutritious foods.^8^ Poverty may also impact young children’s sleep through parental mental health^9^ and the quality of the sleep environment^10^ and bedtime routines.^9,10^

Previous quasi-experimental work suggests that even small increases in income may improve health trajectories for children experiencing poverty. For example, expansion of the Earned Income Tax Credit, the largest antipoverty policy in the United States prior to pandemic-related tax expansions, reduced the incidence of low birth weight infants in the population.^11^ Similarly, exogenous increases in minimum wage were found to improve children’s nutritional status in low-income countries.^12^ However, the health impacts of poverty reduction for otherwise healthy infants in the United States is unknown.

Monetary investment and improvements in parental stress are 2 potential pathways by which we have theorized that poverty reduction may improve developmental trajectories.^13^ For example, greater financial resources may allow parents to invest in high-quality inputs that support child health and sleep, including nutritious foods, preventive medical care, a separate sleep space, and safe housing. Additionally, reduced financial strain may reduce parental stress and mental health symptoms, which may subsequently improve the quality of family interactions, bedtime routines, and child sleep quality.

The present study evaluates the effect of a poverty reduction intervention on children’s health, nutrition, sleep, and health care utilization in the first 3 years of life. Such an intervention exemplifies a scalable public health approach. We hypothesized that a monthly, unconditional cash transfer would improve maternal assessments of children’s health, nutrition, and sleep, and reduce use of emergency health care.

## Methods

### Study Design

Baby’s First Years (BFY) is a parallel-group, randomized clinical trial (RCT) of poverty reduction. Between May 2018 and June 2019, 1000 mothers experiencing poverty were recruited from postpartum wards after giving birth, and were offered a monthly, unconditional cash transfer (referred to as a “cash gift”). Mothers were randomly assigned to either a high-cash gift group (n = 400) or a low-cash gift group (n = 600). The monthly gifts were initially promised for the first 40 months of their child’s life and were subsequently extended through 76 months. More information on study design can be found in Supplement 1 and in Noble et al.^13^

Upon providing written informed consent, mothers completed a baseline interview and received compensation for their participation ($50). For the next 3 years, mothers were invited to complete annual surveys around the time of their child’s birthday. All study procedures were approved by the institutional review board of Teachers College, Columbia University. This study follows the Consolidated Standards of Reporting Trials (CONSORT) reporting guideline for RCTs.

### Participants

Mothers were recruited from 12 hospitals across 4 US metropolitan areas: New York, New York; Omaha, Nebraska; New Orleans, Louisiana; and Minneapolis/St Paul, Minnesota. Eligibility criteria included (1) being of legal age to provide consent; (2) reporting a household income less than the federal poverty line; (3) being able to speak English or Spanish; (4) the infant not being admitted to the neonatal intensive care unit; (5) residing in the state of recruitment; (6) planning to remain in-state within the next year, and (7) infant to be discharged into the mother’s custody.

### Randomization and Masking

Randomization occurred within hospitals, with 60% randomized to the low-cash gift group and 40% randomized to the high-cash gift group. After obtaining written informed consent and conducting the baseline survey, interviewers retrieved the randomized group assignment, informed participants of their assigned group, and activated the debit cards; therefore, interviewers could not be masked to condition during recruitment. However, interviewers were not informed or reminded of participants’ treatment status during follow-up assessments.

### Intervention

Mothers in the high-cash gift group received $333/mo ($3996/y), whereas mothers in the low-cash gift group received $20/mo ($240/y). Funds were disbursed monthly onto an electronic debit card (branded 4MyBaby), accompanied by a text message notification.^13^ The receipt of $4000 in cash gifts increased the average family’s income by approximately 20%.^14^ Provisions instituted by state agencies and legislation ensured the cash gifts affected neither mothers’ eligibility for nor amount of public benefits.

Participants were informed they could spend the money how they wished, and receipt of the funds was not conditioned upon continued participation in the study. Among those who consented to allow access to transaction data (n = 900), only 5 families (all in the low-cash gift group) had not withdrawn funds from the debit card 3 years after randomization.

### Measures

At the baseline interview, mothers provided demographic information including racial and ethnic identity to allow possible exploration of heterogeneity in treatment effects. Participants were provided with the options of American Indian, Eskimo, or Aleut; Asian or Pacific Islander; Black or African American; White; or other for race; ethnicity was assessed by whether the mother identified as Hispanic. Each following year, mothers completed surveys of their child’s health, nutrition, sleep, and health care utilization. Such parent-reported measures are frequently used as proxies for child health outcomes (eg, Page et al^15^) and correlate with objective assessments.^15,16^

The primary outcomes for this RCT used to determine sample size centered on child development at age 4 years^13^ and are beyond the scope of this article. We additionally preregistered primary outcomes in child health and sleep at age 3 years, including an additive Child Health Index which is further described in the eMethods in Supplement 2. The secondary outcomes of interest were child health and sleep disturbances at ages 1 and 2 years and children’s consumption of healthy and unhealthy foods at age 2 years. Outcomes were preregistered at ClinicalTrials.gov. We also explored impact estimates for some outcomes that were not preregistered, including types of postbirth diagnoses, bedtime routines, cow’s milk consumption, vaccinations, missed medical care, Medicaid receipt, and 4 global measures of health, nutrition, sleep, and health care utilization.

### Health Outcomes at Ages 1, 2, and 3 Years: Overall Health and Diagnosis With a Health Condition or Disability

Mothers rated their child’s overall health on a 5-point Likert scale, from 1 (excellent) to 5 (poor). Mothers indicated via a dichotomous item whether their child was diagnosed with a health condition or disability since birth. Mothers who endorsed such a diagnosis were asked to specify the diagnosis, and these responses were coded.

### Nutrition Outcomes at Age 2 Years

On the age-2 survey, mothers responded to 4 items regarding their child’s food intake. The items were adapted from the Los Angeles County WIC Survey.^17^

#### Healthy Foods

Two items assessed how frequently their child ate fruits and vegetables on an average day. Frequency was assessed via a Likert scale ranging from 0 (never) to 5 (≥5 times per day). We created an additive index by summing the total number of times the child consumed produce each day.

#### Unhealthy Foods

Two items assessed how frequently their child consumed sweets (eg, sweetened cereals, fruit bars) and sweetened drinks (eg, juice, chocolate milk) on an average day. Frequency was assessed via a Likert scale ranging from 0 (never) to 5 (≥5 times per day). We created an additive index by summing the total number of times the child consumed unhealthy foods each day.

### Sleep Disturbances at Ages 1, 2, and 3 Years

Sleep disturbances were assessed through the Patient-Reported Outcomes Measurement Information System (PROMIS) Sleep Disturbance–Short Form,^18^ consisting of 4 items assessing the frequency of sleep-related difficulties over the last 7 days using a 5-point Likert scale, 1 (never) to 5 (always). One positively stated item was reverse coded before being summed. Higher scores indicated more sleep disturbances. Mothers needed to respond to at least 3 of the 4 items to obtain a valid score. Due to an administrative error, 1 item was excluded from the age-3 survey. Thus, the score at age 3 years reflects the sum of only 3 items, rather than 4.

### Healthcare Utilization Outcomes at Ages 1, 2, and 3 Years

#### Physician Visits

Mothers responded to 2 items indexing how often they sought health care for their child in the past year: “About how many times in the last year did you take your child to a doctor because they were sick?” and “About how many times in the last year did you take your child to a doctor because they were hurt or injured?” Responses were categorized as 0 to 1 visits, 2 to 5 visits, or 6 or more visits. Across all 3 waves of data collection, most mothers (>92%) reported fewer than 6 or more physician visits in the last year. We collapsed these items into 2 dichotomous indicators, representing whether the mother reported 2 or more physician visits due to illness or injury in the past year.

#### Emergency Department and Urgent Care Visits

Mothers reported the number of times they brought their child to an emergency department (ED) or urgent care center in the past year using a categorical indicator (0 visits, 1 visit, 2-5 visits, or ≥6 visits). Across waves of data collection, between 35% and 54% of mothers reported at least 1 visit, and nearly all mothers (98%-99%) reported fewer than 6 or more visits. For analyses, we created an ordinal variable reflecting whether the mother reported 0, 1, or 2 or more ED or urgent care visits in the last year.

### Missing Data

High response rates were observed at annual follow-up assessments: after adjusting for mother-child separations (n = 2), maternal incarcerations (n = 4), and infant deaths (n = 4), at least 92% of the sample participated in each time point (eFigure in Supplement 2). A total of 857 mothers completed all 3 surveys.

### Statistical Analysis

Intent-to-treat analyses were conducted by fitting a linear regression equation with robust standard errors for each outcome at each age (ie, ages 1, 2, and 3 years). We also examined cumulative impacts of the intervention by pooling across waves for each outcome, treating each observation independently at each wave of data collection (ie, ages 1, 2, and 3 years; 2768 observations). After adjusting for an anticipated 20% attrition by the age-3 assessment, this study was 80% powered to detect an effect of 0.207 SD.^13^

Analyses were adjusted for 27 preregistered covariates measured at baseline (Table 1) and the child’s exact age at the time of the interview. The COVID-19 pandemic began partway through in-person age-1 data collection, at which point data collection pivoted from in-person to telephone surveys (all age-2 and age-3 surveys were collected remotely). We include a dummy variable for survey administration method in the age-1 wave.

**Table 1.** Descriptive Statistics at Baseline^a^

Characteristic^b^	Low-cash gift group		High-cash gift group	
	Mean (SD)	Total No.	Mean (SD)	Total No.
Child sex, No. (%)				
Female	301 (50.17)	600	191 (47.75)	400
Male	299 (49.83)	600	209 (52.25)	400
Child weight at birth, lbs	7.10 (1.08)	599	7.10 (1.01)	399
Child gestational age, wk	39.10 (1.25)	596	39.00 (1.24)	399
Mother age at birth, y	26.80 (5.82)	600	27.40 (5.87)	400
Mother education, y	11.90 (2.83)	593	11.90 (2.96)	398
Mother race and ethnicity, No. (%)				
Black, non-Hispanic	237 (39.50)	600	177 (44.25)	400
Hispanic	243 (40.50)	600	166 (41.50)	400
White, non-Hispanic	67 (11.17)	600	34 (8.50)	400
Multiple, non-Hispanic	24 (4.00)	600	12 (3.00)	400
Other or unknown^c^	27 (4.50)	600	11 (2.75)	400
Mother marital status, No. (%)				
Never married	255 (42.50)	600	198 (49.50)	400
Single, living with partner	156 (26.00)	600	87 (21.75)	400
Married	125 (20.83)	600	86 (21.50)	400
Divorced or separated	30 (5.00)	600	11 (2.75)	400
Other family structure or status unknown	34 (5.67)	600	18 (4.50)	400
Mother health is good or better, No. (%)	527 (87.83)	600	367 (91.75)	400
Mother depression (CES-D)^d^	6.80 (4.52)	600	6.90 (4.61)	400
Cigarettes per week during pregnancy, No.	5.00 (21.17)	595	3.50 (11.76)	397
Alcohol drinks per week during pregnancy, No.	0.20 (1.63)	598	0.00 (0.39)	399
Children born to mother, No.	2.40 (1.38)	600	2.50 (1.41)	400
Adults in household, No.	2.10 (1.00)	600	2.00 (0.96)	400
Biological father lives in household, No. (%)	238 (39.67)	600	141 (35.25)	400
Household combined income, $	22 466 (21 360)	562	20 918 (16 146)	370
Household income unknown, No. (%)	38 (6.33)	600	30 (7.50)	400
Household net worth, $	−1981 (28 640)	531	−3308 (20 323)	358
Household net worth unknown, No. (%)	69 (11.50)	600	42 (10.50)	400
Household receives WIC, No. (%)	407 (68.63)	593	280 (70.53)	397
Household receives SNAP, No. (%)	345 (58.18)	593	233 (58.69)	397

Abbreviations: CES-D, Center for Epidemiologic Studies Depression Scale; SNAP, Supplemental Nutrition Assistance Program; WIC, Special Supplemental Nutrition Program for Women, Infants, and Children.

^a^ Table adapted from Noble et al.^13^

^b^ All variables (excluding WIC/SNAP enrollment) are included as covariates in linear analyses.

^c^ Includes American Indian, Eskimo, or Aleut; Asian or Pacific Islander; other; and unknown.

^d^ Scores range from 0-60, with higher scores reflecting greater depressive symptoms. A score of 16 or greater indicates a risk for clinical depression.

Adjustments for multiple comparisons were made using the Westfall-Young^19^ procedure when analyzing measures within the same construct (ie, health, nutrition, sleep, or health care utilization) in the same wave. Adjusted *P* values are featured in the results below, and statistical significance was set at *P* < .05. Data are publicly available at the Inter-university Consortium for Political and Social Research (ICPSR; ID 37871). Analyses were conducted using Stata version 16 (StataCorp). Data analysis was conducted from July 2022 to August 2023.

## Results

Recruitment details are in the previously published baseline flow diagram (eFigure in Supplement 2).^13^ Of the 1000 randomized mothers, 1.2% self-identified as American Indian, Eskimo, or Aleut; 0.9% as Asian or Pacific Islander; 41.5% as Black or African American; 10.1% as White; 3.6% as being multiple races; and 1.7% as being some other race. Of Hispanic-identifying participants (40.9%), most identified as from either the Dominican Republic (39.7%) or the US (32.3%). Overall, 49.2% of the infants were female.

Table 1 presents descriptive statistics by treatment status for all participants at baseline (1000). On average, infants were of normal birth weight (mean [SD], 6.64 [1.10] pounds) and born at term (mean [SD], 39.06 [1.30] weeks). Four mothers reported their child as deceased by the age-1 visit (3 infants in the high-cash gift group).

Descriptive statistics for primary outcomes are presented in Table 2. At age 1 year, 602 mothers (64.8%) rated their child’s overall health as excellent, 215 (23.1%) rated it as very good, and 93 (10.0%) rated it as good. Across all visits, 2.1% to 3.6% of mothers (19-33 mothers) rated their child’s health as fair or poor. By age 3 years, 96 mothers (10.5%) reported that their child had been diagnosed with a health condition or disability. Autism (n = 28) and asthma (n = 20) were the most frequently reported diagnoses. Across waves of data collection, between 15% and 28% of mothers reported bringing their child to an ED or urgent care center at least 2 times in the past year.

**Table 2.** Descriptive Statistics of Outcome Variables at Ages 1, 2, and 3 Years by Treatment Status^a^

Outcome	Age 1 y		Age 2 y		Age 3 y	
	Low-cash gift group (n = 547)	High-cash gift group (n = 382)	Low-cash gift group (n = 543)	High-cash gift group (n = 376)	Low-cash gift group (n = 542)	High-cash gift group (n = 378)
Child health						
Maternal rating of child’s overall health (1 = excellent, 5 = poor)	1.49 (0.73)	1.51 (0.80)	1.53 (0.76)	1.51 (0.80)	1.56 (0.82)	1.61 (0.87)
Maternal report of whether child has a diagnosis of health condition or disability, No. (%)	68 (12.45)	59 (15.45)	35 (6.46)	25 (0.0665)	53 (9.8)	43 (11.41)
Overall poor health index	2.93 (1.54)	3.10 (1.72)	2.48 (1.48)	2.48 (1.54)	2.51 (1.65)	2.53 (1.55)
Nutrition^b^						
Healthy foods (No. of times consumed per d)	NA	NA	4.18 (2.12)	4.50 (2.16)	NA	NA
Unhealthy foods (No. of times consumed per d)	NA	NA	3.45 (2.21)	3.48 (2.15)	NA	NA
Sleep						
PROMIS–Sleep Disturbance scale^c^	8.02 (3.44)	7.70 (3.36)	7.49 (3.47)	7.61 (3.35)	5.04 (2.61)	5.10 (2.50)
Health care utilization						
≥2 Physician visits due to illness per y (%)	287 (52.47)	214 (56.02)	170 (31.31)	118 (31.38)	172 (31.73)	114 (30.16)
≥2 Physician visits due to injury per y (%)	9 (1.65)	6 (1.57)	18 (3.31)	10 (2.66)	20 (3.69)	6 (1.59)
ED or urgent care visits per y	0.78 (0.83)	0.86 (0.85)	0.54 (0.74)	0.56 (0.76)	0.49 (0.74)	0.50 (0.73)

Abbreviations: ED, emergency department; NA, not applicable; PROMIS, Patient-Reported Outcome Measurement Information System.

^a^ Unless otherwise indicated, values are presented as mean (SD). Except for healthy food consumption, larger values indicate poorer outcomes in each domain.

^b^ Nutrition items were only administered on the age-2 survey.

^c^ One item was mistakenly excluded from the PROMIS-Sleep Disturbance Scale on the age-3 survey, resulting in a lower average score at that age.

### Impacts on Health, Nutrition, Sleep, and Healthcare Utilization

Effect sizes reflecting the standardized treatment impact, divided by the SD of the control group, are reported in Table 3. Marginal effects derived from probit regressions of dichotomous outcomes are presented in the Supplement (eTable 1 in Supplement 2).

**Table 3.** Intent-to-Treat (ITT) Impacts of Unconditional Cash Transfer on Child Health, Nutrition, Sleep, and Healthcare Utilization

Outcome	ES (SE)^a^			
	Age 1 y (n = 929)	Age 2 y (n = 919)	Age 3 y (n = 920)	Cumulative impacts (ages 1-3 y) (n = 2768)^b^
Health outcomes				
Maternal rating of child’s overall health*, z*	0.04 (0.07)	0.01 (0.07)	0.08 (0.07)	0.04 (0.05)
Maternal report of whether child has a diagnosis of health condition or disability^c^	0.04 (0.02)	0.01 (0.02)	0.03 (0.02)	0.03 (0.02)^d^
Nutrition^e^				
Healthy foods consumed per d*, z*	NA	0.17 (0.07)^f^	NA	NA
Unhealthy food consumed per d*, z*	NA	0.03 (0.06)	NA	NA
Sleep				
PROMIS–Sleep disturbance scale*, z*	−0.10 (0.07)	0.05 (0.07)	0.06 (0.07)	0.01 (0.05)
Health care utilization				
≥2 Physician visits due to illness^c^	0.05 (0.4)	−0.01 (0.03)	−0.01 (0.03)	0.01 (0.02)
≥2 Physician visits due to injury^c^	−0.01 (0.01)	−0.01 (0.01)	−0.02 (0.01)	−0.01 (0.01)
ED or urgent care visits*, z*	0.11 (0.07)	0.01 (0.07)	0.04 (0.07)	0.05 (0.05)

Abbreviations: ED, emergency department; ES, effect size; PROMIS, Patient-Reported Outcome Measurement Information System.

^a^ Except for healthy food consumption, larger values indicate poorer outcomes in that domain. ES reflects the standardized difference between the 2 groups, divided by the SD of the control group. Robust SEs are in parentheses. *P* values were adjusted using the Westfall-Young procedure, such that family-wise adjustments for multiple comparisons were made for each statistical test conducted within the same construct in the same wave (ie, 4 families for each time point). Estimates are adjusted for the covariates listed in Table 1 (except Supplemental Nutrition Assistance Program and Women, Infants, and Children program participation), site-based fixed effects, survey administration method (ie, telephone or in-person) at the age-1 survey, and child age at the time of the assessment.

^b^ Cumulative impacts reflect the estimates of the intervention on the respective outcome, pooled across waves (ie, age 1, 2, and 3 years).

^c^ Marginal effects for dichotomous outcomes are available in eTable 1 in Supplement 2.

^d^ *P* < .10.

^e^ Nutrition items were only administered at the age-2 visit.

^f^ *P* < .05.

The cash gift did not impact any child health (effect size range, 0.01-0.08; SE range, 0.02-0.07), sleep (effect size range, 0.01-0.10; SE, 0.07), or health care utilization (effect size range, 0.01-0.11; SE range, 0.03-0.07) outcome at ages 1, 2, or 3 years (Table 3). However, receipt of the cash gift caused significantly higher child consumption of fruits and vegetables at age 2 years, the only age at which it was measured (β = 0.17; SE = 0.07; *P* = .03). Exploratory analyses revealed this effect was explained by increased fruit consumption (β = 0.22; SE = 0.07; *P* < .001) rather than vegetable consumption (β = 0.06; SE = 0.07; *P* = .41). Children in the high-cash gift group consumed 0.22 SDs more servings of fruit per day than children in the low-cash gift group. The cash gift did not alter consumption of sweets and sweetened beverages (β = 0.03; SE = 0.06; *P* = .69).

Exploratory analyses of impacts of the cash gift on postbirth diagnoses, bedtime routines, cow’s milk consumption, vaccinations, missed medical care, and Medicaid receipt are reported in Supplement 2. The Supplement also reports analysis of the preregistered Child Health Index (impact estimates, eTable 2 in Supplement 2; factor analysis, eTable 4 in Supplement 2) and global measures of health, nutrition, sleep, and health care utilization (eTable 3 in Supplement 2).

## Discussion

This preregistered study found that 3 years of monthly, unconditional cash transfers for families experiencing poverty did not improve maternal reports of children’s health, sleep, or health care utilization. However, the cash gifts did lead to increased reported produce consumption at age 2 years. Previous work has found that low-income mothers tend to perceive fresh produce as expensive and inaccessible^20,21^ and may be averse to introducing novel foods that their children may reject.^22^ The cash gifts may have encouraged low-income mothers to take the financial risk of investing in fresh produce.

By design, this sample consisted of infants who did not require neonatal intensive care at birth, of whom the vast majority were born at-term, and of normal birth weight. It is therefore unsurprising that most mothers rated their children as having excellent or very good health, with rates of health conditions or disabilities aligned with national averages. Most studies linking poverty reduction to children’s health do not utilize such exclusionary criteria (eg, Strully et al^23^). Although national estimates vary, between 5% and 14% of all children have a disability, with those experiencing poverty demonstrating higher rates than more economically advantaged peers.^24^ It is possible that positive impacts of the cash transfers on health were less likely among this sample of children who were generally healthy at birth.

Rates of emergency medical use among this sample were higher than the national average for low-income mothers: 11% of children living in poverty reported at least 2 ED visits in 2019, and 14% reported at least 2 urgent care visits that same year.^25^ In comparison, 15% to 28% of this sample reported at least 2 ED or urgent care visits annually. Notably, the mortality rate for this sample was higher than the national average of 2.03 deaths for every 1000 full-term infants.^26^ The lowest rates of emergency and routine medical care use were reported during the COVID-19 pandemic (ie, age-2 and age-3 data collection), suggesting that parents may have altered their care-seeking behaviors in response to the pandemic.

Structural barriers might interfere with preventive care use,^27^ leading to higher emergency health care utilization. Given the complex lives of poor families, a modest increase in monthly income may be insufficient to overcome barriers to accessing a regular medical home, resulting in the high use of emergency health care services. Direct interventions that connect families with services may be more effective at reducing emergency health care utilization among children experiencing poverty (eg, Goodman et al^28^).

We have previously reported that the monthly cash gifts resulted in greater parental investments in their children, both by increasing expenditures on child-focused goods (eg, books and toys) and increasing parental time spent engaged in developmentally supportive activities (eg, reading and playing).^15^ eTable 5 in Supplement 2 provides a summary of recent papers reporting impact estimates of the BFY study. However, we also found no effect of the cash gifts on other potential pathways that may have improved outcomes in health-related domains. For example, the cash gifts did not improve maternal stress, mental health symptoms, or relationship quality,^29^ which are closely associated with the quality of children’s sleep and bedtime interactions.^9^ The cash gifts also had no effect on reported food insecurity, housing quality, or the likelihood of purchasing a crib.^15^ It is possible that the modest cash gifts were simply not large enough to provide relief to families in ways that would improve such social determinants of health. Indeed, although the gifts resulted in a nearly 20% increase in income for the families in the high-cash gift group, most families were still residing in poverty.^15^

The effects of a poverty reduction intervention on children’s health may not emerge until later in development. Many illnesses associated with childhood poverty, such as hypertension, type 2 diabetes, and heart disease, tend to emerge in adolescence or adulthood.^30,31^ Natural experiments such as the Great Smokey Mountain Study report that cash transfers during middle childhood are associated with positive effects on physical and mental health in adulthood.^32^ It will be important to follow the BFY sample to measure the extent to which investments in early childhood may reduce the incidence of disease processes that emerge later in life.^33,34^

An important strength of this study is its large sample, experimental design, low rates of attrition, and preregistered analysis, which represents an improvement over prior studies that have examined associations between income and children’s health, sleep, and nutrition in cross-sectional or observational studies. Additionally, as both groups received a monthly cash gift, there was no confounding between receipt of the cash gifts and either possession of a debit card or receipt of communication from the research team.

### Limitations

A limitation of the present study is that it relies entirely on maternal assessments, which may introduce bias and be less reliable than objective assessments. However, parental report of children’s overall health, medical history, and sleep duration tends to be highly correlated with objective assessments, including abstraction of medical records and actigraphy.^17,35^ Another limitation is reflected in the limited variability on some survey items, potentially indicating that the truncated response options provided were not ideally suited for assessing medical care in young children. Furthermore, to the extent that even a small cash transfer may have promoted positive impacts on health, it is possible that true impacts may have been masked.

## Conclusions

The present study found no effect of monthly, unconditional cash transfers in the first 3 years of life on maternal reports of children’s health, sleep, or health care utilization. However, the cash transfer caused an increase in toddler consumption of fresh produce. Healthy newborns tend to grow into healthy toddlers, and the impacts of poverty reduction on children’s health and sleep may not be fully borne out until later in life. We intend to follow up with the families through middle childhood and will continue to assess the children's health and development over time.

## Notes

Supplement 1.
Trial Protocol and Statistical Analysis Plan

Supplement 2.
eMethods.

eReferences
**eTable 1.** Marginal Effects of the Cash Gift on Dichotomous Outcomes **eTable 2.** Descriptive Statistics and Treatment Impacts of the Child Health Index **eTable 3.** Intent-to-Treat (ITT) Impacts of Unconditional Cash Transfer on Global Measures of Child Health, Nutrition, Sleep, and Health Care Utilization **eTable 4.** Factor Loadings for a 2-Factor Solution of the Child Health Index Items **eTable 5.** Findings From Current Published Papers, Working Papers, and Preprints Reporting BFY Impacts **eFigure.** Flow Diagram for Baseline, Age-1, Age-2, and Age-3 Data Collection

Supplement 3.
Data Sharing Statement
