# Errors in Results, Table 2, Table 3, and Supplement 2

**DOI:** 10.1001/jamanetworkopen.2024.6438

**Published:** 2024-03-20

In the Original Investigation titled “Unconditional Cash Transfers and Maternal Assessments of Children’s Health, Nutrition, and Sleep: A Randomized Clinical Trial,”^1^ published September 29, 2023, the preregistration document stated that all linear analyses would be standardized to the control group; however, the analyses conducted for this paper were standardized to the full sample. The corrected article^1^ includes changes in the second paragraph of the “Impacts on Health, Nutrition, Sleep, and Health Care Utilization” subsection in the Results section; Table 3; and Supplement 2. Text was also added to the Methods section to clarify the standardization process. Additionally, the mean (SD) birth weight reported should have read 7.11 (1.05) pounds, and the percentages for maternal report of child’s diagnosis in Table 2 have been updated. This article has been corrected.^1^

## References

[1] Sperber JF, Gennetian LA, Hart ER, . Unconditional cash transfers and maternal assessments of children’s health, nutrition, and sleep: a randomized clinical trial. JAMA Netw Open. 2023;6(9):e2335237. doi:10.1001/jamanetworkopen.2023.35237 37773497 PMC10543132

