# Supplementary material for: Unconditional Cash Transfers and Maternal Assessments of Children's Health, Nutrition, and Sleep: A Randomized Clinical Trial
Source: JAMA Netw Open. 2023 Sep 29;6(9):e2335237. doi: 10.1001/jamanetworkopen.2023.35237 (PMC10543132; doi:10.1001/jamanetworkopen.2023.35237)
Supplement: Supplement 1. — Trial Protocol and Statistical Analysis Plan [file jamanetwopen-e2335237-s001.pdf]

TEACHERS COLLEGE

COLUMBIA UNIVERSITY

Institutional Review Board

Box 151 | 525 West 120th St. New York, NY 10027

212-678-4105 | RH 13 | IRB@tc.edu | tc.edu/IRB

IRB APPLICATION TEMPLATE

SECTION I: PROTOCOL DESCRIPTION

1. Study Title: Baby's First Years

Research Summary/lay abstract:

Recent advances in developmental neuroscience suggest that experiences early in life have profound and enduring influences on the developing brain. Family economic resources shape the nature of many of these experiences, yet the extent to which they affect children's development is unknown. Our team of neuroscientists, economists and developmental psychologists proposes to fill important gaps in scientific knowledge about the role of economic resources in early development by evaluating the first randomized controlled trial to determine whether unconditional cash payments have a causal effect on the cognitive, socio-emotional and brain development of infants and toddlers in low-income U.S. families. Specifically, 1,000 mothers of infants with incomes below the federal poverty line from four diverse U.S. communities will receive monthly cash payments by debit card for the first 76 months of the child's life. Parents in the experimental group will receive $333 per month ($4,000 per year), whereas parents in the control group will receive a nominal monthly payment of $20. In order to understand the impacts of the added income on children's cognitive and behavioral development, we will assess treatment/control group differences at age 4, 6 and 8 (and, for a subset of measures, ages 1, 2 and 3) on measures of cognitive, language, self-regulation and socio-emotional development. Brain circuitry may be sensitive to the effects of early experience even before early behavioral differences can be detected. In order to understand the impacts of added income on children's brain functioning at age 4, 6, and 8, we will assess treatment/control group differences in measures of brain activity (electroencephalogram [EEG] and event-related potentials [ERP]).

To understand how family economic behavior, parenting, and parent stress and well-being change in response to income enhancement, we will assess treatment/control differences in family expenditures, food insecurity, housing and neighborhood quality; family routines and time use; parent stress, mental health and cognition; parenting practices; and child care arrangements at child ages 4, 6, and 8, and, for a subset of these measures, child age 1, 2, and 3. This study will thus provide the first definitive understanding of the extent to which income plays a causal role in determining child cognitive, socio-emotional and brain development among low-income families.

Furthermore, in response to the ongoing Covid-19 pandemic, the study will collect information on masking practices and relate that to children's development.

2. Principal Investigator (*person conducting the research*). Professional title and email.

- Kimberly Noble (kgn2106@tc.columbia.edu), Professor of Neuroscience and Education, Teachers College, Columbia University

- Katherine Magnuson (kmagnuson@wisc.edu), Vilas Achievement Professor of Social Work, Director, Institute for Research on Poverty, University of Wisconsin, Madison
- Greg Duncan (gduncan@uci.edu), Distinguished Professor of Education, University of California, Irvine
- Nathan Fox (fox@umd.edu), Distinguished University Professor, University of Maryland, College Park
- Lisa Gennetian (lisa.gennetian@duke.edu), Pritzker Professor of Early Learning Policy Studyies, Sanford School of Public Policy, Duke University
- Hirokazu Yoshikawa (yoshikhi@gmail.com), Courtney Sale Ross Professor of Globalization and Education at NYU Steinhardt, Professor of Applied Psychology, Co-Director of the Global TIES for Children center, New York University

3. Write an original, brief, non-technical description of the purpose of your research. Include a narrative that explains the major parts of your study and how the data will advance your research hypothesis or question. ***NOTE:*** *This section should be easy to read for someone not familiar with your academic discipline. Provide relevant background information and scientific justification for your study. You may provide citations as necessary. Please adhere to a 350-word limit (not including citations).*

A growing body of small-scale studies documents that the cognitive and brain development of low-income children differs from that of children reared in higher-income families. Differences favoring more affluent children are found for young children's language, memory, executive function, and socioemotional processing, with corresponding differences in neural structure and function in brain regions that support these skills [1]. At the same time, a large body of social science research, including quasi-experimental designs, documents income disparities in more general measures of children's achievement, school performance, and learning-related behaviors such as attention and self- regulation [2]. Developmental scientists agree that poverty is especially likely to shape children's early development because of the high plasticity and rapid growth of the brain during the first three years of life. Yet, there has not been a rigorous study of how income supports for families affect the brain function and development of infants and toddlers.

A large social science literature has documented that poverty is associated with lower quality caregiving environments [3], but these studies are largely correlational, precluding strong conclusions about the causal nature of the associations. Our study will estimate causal impacts of increased income, as generated by unconditional cash supplements, on key dimensions of family life, which we conceptualize in two main pathways. First, economic models view families with greater income as better able to purchase or produce important inputs into their young children's development. This ***investment pathway*** results in children experiencing more materially enriching early environments. Second, psychologists and sociologists point to the ways in which economic disadvantage impairs children's development through a ***stress pathway.*** This pathway includes income effects on the quality of family relationships [4], as well as on biological indices of chronic stress [5]. These pathways have shown distinct associations with developmental outcomes, with generally stronger impacts of investment on early cognitive skills and stronger impacts of the stress constructs on early socio-emotional skills.

Results of this study will provide strong and clear evidence about the magnitude and pathways of causal connections between poverty reduction in the early years of life and child cognitive and brain development. Beyond its core contributions to science, the proposed large study will provide important evidence about the likely effects of tax and income-enhancement policies for young children, such as the Child and Earned Income Tax Credits, incentive-based employment programs, and related social policies designed to enhance family economic well-being.

Masking practices and children's development, a supplement to the Baby's First Years primary aims

Young children rapidly acquire language and socioemotional skills through their interactions. Scientific evidence points to the importance of the quantity and quality of children's early experiences, and particularly their interactions with caregivers. As a result, the context of the Covid-19 pandemic, which has potentially changed the nature of children's interactions with others, raises important questions about whether children's development has been impacted. In particular, there has been speculation and concern that mask wearing may interfere with children's language and socioemotional development. In the absence of research, scant evidence can confirm or refute these concerns. Although mask wearing is a vital component of infection control and crucial for public health, the long-term consequences of mask wearing for child development are unknown. Questions in the age 3 instrument will characterize the mask-wearing behavior of low-income mothers and their three-year-old children in and outside of the home. We will use that information to relate mask wearing in the child's environment (e.g., by their mother, peers, care providers, and relatives) as well as the child's own mask-wearing behavior to (a) the child's language and socioemotional development as reported in a maternal interview at age 3, and (b) the child's brain activity and neurocognitive measures of language learning, assessed through laboratory-based measures at age 4.

[1] Duncan G and Magnuson K, (2012) "Socioeconomic status and cognitive functioning: Moving from correlation to causation" Wiley Interdisciplinary Reviews: Cognitive Science, 3:377–386; Noble, K. G. *et al.* Family income, parental education and brain structure in children and adolescents. *Nature Neuroscience* **18**, 773-778, doi:doi:10.1038/nn.3983 (2015). PMCID: PMC4414816; Hackman, D. & Farah, M. J. Socioeconomic status and the developing brain. *Trends in Cognitive Sciences* **13**, 65-73, doi:10.1016/j.tics.2008.11.003 (2009); Brito, N. H. & Noble, K. G. Socioeconomic status and structural brain development. *Frontiers in Neuroscience* **8**, doi:10.3389/fnins.2014.00276 (2014). PMCID: PMC4155174
[2] Akee R, Copeland W, Keeler G, Angold A, and Costello J (2010). "Parents' incomes and children's outcomes: A quasi-experiment." American Economic Journal: Applied Economics 2010, 2(1):86–115; Dahl, G. B. & Lochner, L. (2012). "The impact of family income on child achievement: Evidence from the Earned Income Tax Credit," American Economic Review, 102(5): 1927-56; Duncan GJ, Morris PA, Rodrigues C. (2011). "Does money really matter? Estimating impacts of family income on young children's achievement with data from random-assignment experiments." Developmental Psychology, 47(5):1263–1279.
[3] Yeung, W. J., Linver, M. R. & Brooks–Gunn, J. How money matters for young children's development: Parental investment and family processes. *Child Development* 73, 1861-1879 (2002); Guo, G. & Harris, K. M. The mechanisms mediating the effects of poverty on children's intellectual development. *Demography* 37, 431-447 (2000); Linver, M. R., Brooks-Gunn, J. & Kohen, D. E. Family processes as pathways from income to young children's development. *Developmental Psychology*

38, 719-734 (2002); Kaushal, N., Magnuson, K. & Waldfogel, J. in *Whither Opportunity? Rising Inequality, Schools, and Children's Life Chances* (eds Greg J Duncan & Richard J Murnane) 187-205 (Russell Sage Foundation, 2011); Conger, R. D. *et al.* Economic pressure in African American families: A replication and extension of the family stress model. *Developmental Psychology* 38, 179-193, doi:10.1037/0012-1649.38.2.179 (2002); McLoyd, V. C. The impact of economic hardship on Black families and children: Psychological distress, parenting, and socioemotional development. *Child Development* 61, 311-346, doi:10.1111/j.1467-8624.1990.tb02781.x (1990).
[4] Conger, R. D. *et al.* Economic pressure in African American families: A replication and extension of the family stress model. *Developmental Psychology* **38**, 179-193, doi:10.1037/0012-1649.38.2.179 (2002); Mistry, R. S., Vandewater, E. A., Huston, A. C. & McLoyd, V. C. Economic well-being and children's social adjustment: The role of family process in an ethnically diverse low-income sample. *Child Development* **73**, 935-951 (2002); Conger, R. D. *et al.* Family economic stress and adjustment of early adolescent girls. *Developmental Psychology* **29**, 206-219 (1993)
[5] Evans, G. W. The environment of childhood poverty. *American Psychologist* **59**, 77-92, doi:10.1111/j.1467-9280.2007.02008.x (2004); Evans, G. W. & English, K. The environment of poverty: Multiple stressor exposure, psychophysiological stress, and socioemotional adjustment. *Child Development* **73**, 1238-1248, doi:10.1111/1467-8624.00469 (2002); Van Uum, S. *et al.* Elevated content of cortisol in hair of patients with severe chronic pain: A novel biomarker for stress. *Stress* **11**, 483-488, doi:10.1080/10253890801887388 (2008); Dettenborn, L., Tietze, A., Kirschbaum, C. & Stalder, T. The assessment of cortisol in human hair: Associations with sociodemographic variables and potential confounders. *Stress* **15**, 578-588, doi:10.3109/10253890.2012.654479 (2012)

4. State your research question(s). Your planned research protocol should be one that can realistically address your research question(s).

1) What are the impacts of cash payments made to low-income families in the early years of life on children's school achievement, cognitive and behavioral development?
   This question will be addressed by assessing treatment/control group differences at age 4, 6, and 8 (and, for a subset of measures, age 1, 2, and 3) in validated and reliable measures of cognitive, language, self-regulation and socio-emotional functioning
2) What are the impacts of cash payments on low-income children's brain functioning at age 4, 6, and 8?
   This question will be addressed by assessing treatment/control group differences using electroencephalographic-based measures of brain circuitry, including resting brain activity as well as the neural signatures of vocabulary development and memory formation.
3) How are family and child developmental contexts affected by unconditional cash supplements?
   To address this question we will assess treatment/control differences in family expenditures, food insecurity, housing and neighborhood quality, routines and time use, parent stress, parent-child interactions, parenting practices and child care arrangements at child age 4, 6, 8, and, for a subset of measures, at child ages 1, 2, and 3.

5. Provide the inclusion criteria for the participant population (*e.g., by gender, class, race, occupation, or age*). Provide a rationale for selecting this population for research purposes.

- Participant is a mother who gave birth to an infant in the well baby nursery Mother's household was at or below the poverty line in the last calendar year
    - Rationale: We aim to see the impact of cash payments on families living below the federal poverty line over the span of the baby's first three years of life.
- Participant is able to provide at least one piece of contact information in order to re-contact them for future visits
    - Rationale: We would like to continue to follow up with the mother and child three times after recruitment; contact information is necessary to do follow up data collection.

6. Federal guidelines state that research cannot exclude any classes of participants without scientific justification. Indicate who will be excluded from your study and why (*e.g., persons under 18 years of age*).

- Maternal age < 18 years (with the exception of Nebraska <19)
- Mother not fluent in English or Spanish
- Multiple gestation pregnancy
- Family lives out of the state
- Family lives in the state and indicates they are very likely to move
- Baby will not go home with the mom

7. Provide the maximum number of participants you plan to enroll for each participant population and justify the sample size.

1,000 mothers of newborn infants with household cash incomes in the prior calendar year below the federal poverty line for households of their size (including the infant as a household member).

Study sample description is listed below:

Full study sample:

The proposed study will enroll a total of 1,000 mothers of newborn infants with household cash incomes in the prior calendar year below the official poverty line for households of their size (including the infant as a household member). We plan to identify 1,200 eligible mothers, with 1,000 participants expected to take up the opportunity of a cash gift. Enrollment will be evenly distributed across four sites: New York City; New Orleans; the Omaha and Lincoln metropolitan areas; and Minneapolis/St. Paul [HOSPITAL NAMES REDACTED FOR PARTICIPANT PRIVACY.] The study investigators and local partners have successfully completed studies and recruited mothers in these hospital settings, including the current pilot study. The four sites were selected because they are diverse in terms of racial and ethnic composition of low-income residents, cost of living, urbanicity, and the benefits of state safety net programs such as Temporary Assistance to Needy Families (TANF) and state Earned Income Tax Credits (EITC).

Census data for our hospitals' catchment areas suggest our sample will be roughly 35% African American, 45% White (including Hispanics), 7% Asian, 14% other; 20% are expected to also identify as being Latino.

Site-specific sample:

Please see Appendix B for letter of support from each of the hospitals as well as table indicating expected number of participants to be recruited by site.

8. Describe your recruitment methods. **How** and **where** will participants be recruited (*e.g., flyers, announcements, word-of-mouth, snowballing, etc.*)? Submit a copy of all recruitment letters, scripts, emails, flyers, or social media posts you plan to use to recruit participants for your study as separate documents with your application. You will need to include your IRB Protocol number (*e.g., 18-123*) on all recruitment materials, including announcements, online posts, and email text, etc.

Recruitment (and our three subsequent annual data collections) will be spread evenly over the course of 12 months to avoid possible impacts of seasonal variation and to keep interviewer workload manageable. The Survey Research Center (SRC) at the University of Michigan will recruit/enroll participants and conduct baseline surveys. SRC is especially skilled in fielding national studies that replicate protocols across multiple sites with high fidelity and provide data for the ongoing monitoring of the quality of recruitment efforts and data integrity. Extra quality assurance of these elements, as well as all age-4, 6, and 8data collection, will be conducted by our research team.

Mothers will be recruited in postpartum wards of participating hospitals shortly after giving birth (98.6% of all births in the US in 2012 occurred in hospitals, with a higher percentage among births to low-income, Black and Hispanic women). Nursing staff on the postpartum wards will indicate which rooms house mothers whose infants are admitted to the well-baby nursery, and if there are any mothers who, in their professional judgment, should not be approached to participate in a research study. Initial contact will be made in person by SRC researchers, who approach mothers to ask if they are interested in participating in a research study, explain the general purposes and procedures of the study (with the exception of the cash gift) They will explain the study using the study brochure (attached). At [REDACTED FOR PARTICIPANT PRIVACY] Hospital, this initial contact determining research interest will be done by a clinical research nurse instead of the SRC interviewer. From this point forward the protocol will be implemented equally across sites. If the mother is interested, she will complete a short screening interview to determine eligibility (Eligibility Form A adapted into a computer-assisted instrument BFY_Intro-Screener_v10_TC_IRB.docx.), which is based on income and several other inclusion/exclusion criteria. Information from Eligibility Form A will be kept, recorded, and used for research purposes for all participants, in order to understand enrollment processes. After determining eligibility and interest in participating in a baseline interview and subsequent data collection, mothers will be administered the Research Consent Form and HIPAA Form, complete the Baseline Questionnaire, and receive compensation for this interview. After completing the baseline survey, the interviewer will ask the participant for forms of contact for future waves of participation, including phone number(s), email address(es), address(es), and contact information for friends/family members who would know how to reach the participant if the participant's own contact information becomes invalid. We will also collect social media contact information. Social media will only be used as a method of contacting the participants if other methods fail; we will only use social media as a means to arrange research-related communications via other more private means. At a minimum,

participants will need to provide at least one of the above forms of contact. Participants who fail to provide at least one of the above forms of contact will not be invited to participate in future data collection and will not be offered the cash gift.

Next, mothers will be given the option to participate in the cash gift, which is NOT contingent on continued research participation. This sequence of events is designed to avoid coercion in the enrollment process. Mothers will be explained about how the debit card works, where they may use the card, and how to contact our team in the event of loss or theft or other difficulties in using the card. They will also be explained that the gift is not taxable and should not be reported on tax returns. Finally, mothers will be explained how receipt of the cash gift does or does not affect receipt of other social services and benefits. (Please see gift letter, attached.)

In the state of Nebraska, as added assurance that the cash gift is not counted toward determining eligibility for these benefits, the Nebraska Department of Health and Human Services has requested the names, address and date of birth of mothers receiving the gift. If a mother refuses to allow provision of this information to the Nebraska DHHS, she may still elect to receive the gift, and extra steps may have to be taken on her behalf to ensure that the gift is not counted in determining eligibility. We note that Nebraska passed legislation to ensure that the gift does not impact eligibility, and thus we are confident that mothers will not lose eligibility for listed services as a result of the gift.

In the state of Minnesota, as added assurance that the cash gift is not counted toward determining eligibility for these benefits, the [REDACTED FOR PARTICIPANT PRIVACY] County Human Services and Public Health Department and [REDACTED FOR PARTICIPANT PRIVACY] County Public Health have requested the names, address and date of birth of mothers receiving the gift. If a mother refuses to allow provision of this information, she may still elect to receive the gift, and extra steps may have to be taken on her behalf to ensure that the gift is not counted in determining eligibility. We note that Minnesota passed legislation to ensure that the gift does not impact eligibility, and thus we are confident that mothers will not lose eligibility for listed services as a result of the gift.

Before activating the debit card, we will debrief the participants by explaining that the purpose of the study is to gain an understanding of how receiving a cash gift might affect the experiences of families with new babies. Specifically, we will be studying the effects of the cash gift on family life and child development.

Participants will then be randomly assigned to either the experimental group (40% - $333/month for 40 months) or the control group (60% - $20/month for 40 months). Due to COVID-19 and the plans for delaying the in-person activities, the payments were extended to month 52. We propose a second extension of monthly cash gift payments to bring the total number of payments to 76. The interviewer will activate the debit card for the participant, assign a pin number, and provide instructions about its use. Participants receive their first payment on the card at this time

We will request permission to optionally track spending on the debit card (also on the Transaction Tracking and Form), although it will be made clear that the cash gift participation is 100% free of contingencies, and as such this additional research participation is optional and voluntary. Debit card tracking identifies the type of merchant or facility where the card was used, the amount spent, and the date/time of the usage. The report will not include information regarding the specific items purchased. We will also request permission to obtain their full or last four digits of their social security number in order to access data on the services and benefits the family receives to study how government social services and benefits that participating families receive may be affected by the gift.

This voluntary research involves a home visit or phone interview at age 1, phone interviews at ages 2 and 3, and lab visits at ages 4, 6, and 8. Participants will have the opportunity to accept or decline data collection activities at each wave, regardless of their initial consent to participate in the longitudinal study.

All of the enrollment procedures outlined above worked well in our pilot study, conducted in New York City in the summer-fall of 2014.

Although a small number of women may then choose to take the cash payments and decline to participate in the future research, we think this is quite unlikely as (1) participants will have already indicated a willingness to participate in future data collection efforts, (2) SRC has an outstanding track record of recruitment and retention and (3) the research activities will be separately compensated. None of the 30 pilot study mothers refused our interview requests.

9. Describe the location, setting, and timing of data collection (*e.g., face-to-face interview at a mutually convenient location, at the start of the semester*). Include the state, city, school district, etc. ***Note:*** *If you are recruiting participants from institutions other than Teachers College include a site permission form (template located in Mentor/Documentation) or a pending IRB approval from the institution(s) with this submission. If you are conducting any part of your research within NYC DEPARTMENT OF EDUCATION (DOE) Schools, it is required that you receive approval from Teachers College IRB prior to submitting your application to the Department of Education IRB (DOE IRB).*

Data Collection Procedures

We will collect a host of age-appropriate measures of children's cognitive, behavioral and brain functioning, as well as maternal/family process characteristics. The University of Michigan Survey Research Center (SRC) will collect baseline, age 1 , age 2, and age 3 data collection. . Research assistants will conduct data collection during the lab visits at ages 4, 6, and 8. U-M SRC is especially skilled in fielding national studies that replicate protocols across multiple sites with high fidelity and provide data for the ongoing monitoring of the quality of recruitment efforts and data integrity. Extra quality assurance of these elements, as well as all age-4, 6, and 8 data collection, will be conducted by our research team.

At time of recruitment, a baseline survey will be conducted to obtain information about demographic characteristics, economic habits, maternal health/mental health, housing, and other family process constructs.

U-M SRC and the research team will work collaboratively between all study waves to conduct mid-wave follow up with respondents, to verify contact information. The follow up may be conducted via face-to-face visits, mail (by sending a postcard asking for address confirmation), email, phone, or by sending a text messages.

At age 1, a phone interview or home visit will be conducted with families in order to collect information about child health and general developmental milestones, as well as family process measures. U-M SRC interviewers will conduct age 1 data collection.

At age 2, a maternal phone survey will be conducted to collect additional family process data with questions related to language development, mother-child behavioral interaction quality, and generalized child development information. U-M SRC interviewers will conduct age 2 data collection.

At age 3, the maternal phone survey will again collect family process information. To ensure retention, after the age 3 maternal survey at or around 36 months we will follow up with the families up to 3 times at months 39, 42, and 48 with a brief phone call or a visit to update contact information and, during the last check-n to invite them to the lab visit at age 4. U-M SRC interviewers will conduct age 3 data collection and mid-wave follow up efforts.

At age 4, the primary focus of the lab visit will be to collect measures of children's cognitive, behavioral, and brain functioning (EEG/ERP), as well as to collect data on maternal and child stress physiology (epigenetic aging), maternal executive function, and maternal surveys on family life and well-being. Local research assistants managed by the research team will conduct age 4 data collection.

At ages 6 and 8, the primary focus of the lab visits will be to collect measures of children's school achievement, children's cognitive, behavioral, and brain functioning (EEG/ERP), as well as to collect data on maternal and child stress physiology (epigenetic aging), maternal executive function, and maternal surveys on family life and well-being. Local research assistants managed by the research team will conduct ages 6 and 8 data collection.

Locations of data collection:

All recruitment, initial consent, and baseline interview procedures will take place in the well-baby nurseries of the following hospitals:

[HOSPITAL NAMES REDACTED FOR PARTICIPANT PRIVACY]

All lab visits will take place at in the laboratory of identified PIs at each city and will be conducted by the investigator's research assistants. The following is a list of the Site PIs, who direct the labs where the ages 4, 6, and 8 lab visits will take place:

Minnesota
University of Minnesota - Michael Georgieff, MD

New York
Teachers College Columbia University - Kimberly Noble, MD Ph.D.

Louisiana
University of New Orleans – Sarah Black, Ph.D (until April/2023) and Debra Kahrson, Ph.D (starting on April/2023)

Nebraska
University of Nebraska Lincoln - Timothy Nelson, Ph.D. & Jennifer Nelson, Ph.D.

Reliance agreements for the three sites outside of New York City have been established under this protocol.

SECTION II: DESCRIPTION OF STUDY ACTIVITIES & PROCEDURES

10. List what your participants will be asked to do during your study and your data collection process (*e.g., fill out a 25-question, closed-ended, paper survey*). ***Note:*** *Submit copies of all instruments, surveys, interview questions, observation checklists, etc. that you plan to use for data collection as separate documents. Indicate whether data are collected as part of an initial participant screening or the actual study. If you have multiple participant groups (e.g., parents, teachers, and students or control groups and experimental groups), please specify which group you are asking to complete which task(s). If applicable, submit separate translated copies of all questionnaires, interview questions, consent forms, and recruitment materials, for each participant population. Upload a copy of the back-translation (translation into the target language and back into English) document using Google-Translate to validate translation accuracy. Alternatively, the translator can sign the "translation verification" form in Mentor/Documentation.*

Intervention Procedures

The intervention in this project is a simple income manipulation in the form of cash payments to families, funded by philanthropy. Mothers in the treatment group will receive monthly cash payments of $333 ($4,000 per year) for the first 76 months of the child's life, paid on the day of the month of the child's birthday by automatic loading on an electronic debit card. To put the magnitude of the payments in context, the proposed annual income supplement of $4,000 would increase income in the average poor family in our pilot study by about 20%. Monthly text messages will announce the payment to recipients. In order to eliminate any payment-mode effect, the control group will receive a nominal payment -- $20 per month, delivered in the same way as for the experimental group. Debit cards are a superior mode of cash transfer for practical reasons (e.g., few low-income individuals have bank accounts) as well as for conceptual reasons: We wanted to preserve the unconditional cash transfer nature of the transaction and enable point-of-sale or ATM cash transactions. As in the pilot study, Greenphire will administer the debit cards to the mothers. Our pilot study experience suggests that all mothers will use their debit cards regardless of experimental condition, thus leading us to expect that take up will be near-universal.

We will provide support to families in the event the card is lost or stolen. Additionally, we will follow up with mothers who whose cards have a protracted period of inactivity. The Project Director will contact the mom to confirm they have the card and know how to use the card.

Each phase of pilot testing and data collection are described below.

Phase 1 Pilot Testing: Baby's First Years Baseline Survey

Description: We propose to pilot the baseline survey on mothers whose demographic characteristics are similar to those who will be recruited at the hospitals, specifically, age and socioeconomic status.

Purpose: We have programmed the administration materials in Blaise, a computer assisted interview software tool. This will facilitate large-scale data collection across all hospitals. By testing 3-5 mothers we will confirm that length of the baseline survey does not exceed our proposed one-hour length. We will also understand if any minor script modifications would facilitate clarity of the administration materials to participants. Please note that families will not be enrolled in the cash gift, as the intended purpose of this pilot is solely to assess administration of the survey materials.

Recruitment of Participants: Dr. Kimberly Noble maintains a database of families in NYC that have participated or expressed interest in participating in studies of child development. Families in the database with infants 6 – 24 months of age will be invited to participate in the pilot testing of a survey about their experiences and family life.

Confidentiality: Participants' responses will be assigned a subject ID and their names will not be linked to their responses. Responses will not be used as part of data collection or data analysis for the larger study. They will exclusively be used to inform research study design.

Compensation: Moms participating will receive $50 for completing the hour survey and $30 or a car service for transportation costs.

Location: Pilot testing will take place in the Neurocognition Early Experience and Development Lab at Teachers College, Columbia University.

Procedure:

Consent: Mom will consent to participating in the pilot for a study of child development. (See Pilot Consent Form). At this point the mom will receive a $50 compensation.

Pilot Testing: The interviewer will administer Eligibility Form A and the Baseline Questionnaire. The mothers participating in the pilot will NOT be enrolled in a debit card.

Risks: There are no anticipated risks for participating in the pilot testing of the administration materials.

Instrument: "BFY_Baseline_Questionnaire"

Phase 2 Pilot Testing (site-based): Baby's First Years Age 1 Visit

Description: Phase 2 of the pilot testing for the age 1 visits will extend pilot testing to the other sites, and include University of Michigan interviewers. All activities will happen as described in "Phase 1 Pilot Testing" above, except for the following differences:

Purpose: In phase 2 of pilot testing, we plan to run through the Age 1 Study visit with 3-15 mothers in all four study sites, with University of Michigan interviewers who will conduct the actual visits. These pilot visits will allow us to ensure that EEG can be administered by the team that will conduct the visits with BFY participants, and that is the only component of the visit that will be tested.

Recruitment of Participants: Participants will be recruited with the help of local site PIs through flyers.

Consent: Mothers will consent to participating in the pilot for a study of child development on behalf of themselves and their infant (see Age 1 Phase 2 Pilot Consent Form).

Compensation: Mothers participating in this study will receive $50 for participating in the visit. Compensation is not contingent on completing particular components of the visit, and families can choose to stop participating at any time.

Instrument: "BFY_Age 1 Instrument"

Age 1 Data Collection Protocol

Overview

In Baby's First Years, Age 1 participants recruited in the Baseline will be asked to participate in a home visit scheduled within two weeks before or after their child's first birthday. The Age 1 visit will be scheduled and conducted by a University of Michigan Survey Research Center (U-M SRC) interviewer. Before the visit, all families will be sent brief letters with advance information and a pre-payment check of $50 or a small study gift (a book, toy, or blanket). Interviewers will follow up with scheduling phone calls (or emails and text messages if more appropriate) to arrange for the best time to conduct the visit. Visits are estimated to last around 90 minutes including a 45 minute questionnaire and three additional components: a child electroencephalogram (EEG), mother's hair collection for cortisol analysis, and a video-recorded parent-child interaction activity. At the beginning of the visit, the interviewer will present the consent form, give participants an opportunity to ask any questions, and ask for a digital signature before the interview begins. Participants who did not or could not cash the check will receive $50 in cash in appreciation for their time.

Age 1 Visit Protocol

Eligibility

All participants who consented to participate in the Baby's First Years Study at the time of their child's birth, completed the Baseline interview, and provided at least one piece of contact information will be invited to participate in Baby's First Years Age 1 visit, regardless of whether or not they accepted the BFY Monthly Gift (via the 4MyBaby debit card). Participants will also have an opportunity to withdraw at any time. If participants ask to withdraw from the study, they may be contacted again to confirm their withdrawal and to determine the reasons for withdrawal. However, they will not be contacted for the purpose of conducting the Age 1 interview unless they re-consider and decide to continue participation. Participants who withdraw from the study will continue receiving the BFY Gift., unless they specify that they no longer want to receive it.

Sample Release and Pre-visit Activities

We aim to complete Age 1 visits within 2 weeks before or after the first birthday of the recruited child although some participants may end up being interviewed earlier or later than planned. U-M data collection team will send an advance mailing inviting all mothers to participate in the Age 1 visit. The mailing will be sent approximately 4 – 6 weeks before the first birthday of the recruited child. Approximately, 2 – 4 weeks before the first birthday, the sample will be released to interviewers and they will start contacting participants via phone (or email and text messages as appropriate) and scheduling visit appointments.

The advance mailing will include:
a) An introductory letter thanking participants for being part of the study and reminding them they will be contacted again soon (see: BFY_Age1PrecontactLetter)
c) A prepayment check of $50 or a study gift (a book, toy, or blanket). A decision on whether or not to mail a prepayment or a study gift will be based on cost and expected efficiency of each approach.

Participants who provided an email address may additionally be sent advance information via email (see: BFY_Email_Protocol). Participants who provided a cell phone number may also receive a short text message sent by the interviewer once the sample is released (see: BFY_Text Message Protocol).

Interviewer Contact Protocol

Once sample is released, interviewers will attempt to reach participants using contact information obtained during Baseline recruitment. Interviewers will begin by calling available phone numbers on various days and times of day such as weekday morning, afternoon, or evening, as well as weekend morning, afternoon or evening. If U-M interviewer reaches an answering machine/voicemail response they will leave a scripted message, which does not reveal the study name (see: BFY_VoicemailScript). Interviewer will leave their name and a phone number and ask for a call back to either the interviewer work number or the U-M toll–free number.

If participants cannot be reached using the available phone numbers, interviewers may attempt to go in person to the address provided during recruitment. Interviewers may leave "Sorry I Missed You" (SIMY) cards with interviewer work number or the U-M SRC's toll free number (see: BFY-SIMY_Card.doc), possibly with a small gift. The interviewer may also refer hard-to-reach participants to a specialized U-M SRC locating team. The locating team calls available numbers and

uses directory assistance to obtain additional information. The locating team may also conduct internet searches to obtain updated address or telephone numbers. Additionally, at the time of recruitment, some participants provided alternative contact information including phone, email, or address of the best person to contact if the participants are hard to find. Interviewers and/or the locating team may reach out to designated contact person(s) asking for help in reaching the participant. They may also ask contact person(s) to let the respondent know we are trying to reach them and ask them to contact the U-M SRC toll-free number. In some cases, we may offer a small finder's fee of $5 to $10 in appreciation for assistance in locating a BFY participant.

Depending on available contact information, interviewers may also try to reach participants via email or text messages. Email and text messages may also be used to remind respondents about upcoming appointments. (see: BFY_Email_Protocol.doc and BFY_Text Message Protocol.doc)

Upon reaching participants, interviewers will confirm whether participant received an advance letter with BFY Age 1 visit information and whether they have any further questions. Interviewers will respond to any questions and setup an appointment to conduct the Age 1 in-person visit. Interviewers will schedule the visit for a time most convenient for the participant. Interviewers will be asked to try to schedule a visit on a specific date designated by the study team, however, if that date does not work for participants they will be able to choose a more suitable date and time.

Unexpected situations

During the pre-visit contact, the interviewer may come across information that will require a specific course of action or to provide the mother with more information about her participation in the BFY study. Although it isn't possible to predict all of the special circumstances that may arise, we've outlined four potential situations, below. Participants who cannot take part in the Age 1 visit will continue to receive the BFY Gift unless they specifically opt-out and choose not to receive cash payments.

State Residence and Eligibility for Social Services

Prior to scheduling a visit, interviewers will confirm a participant's current address. Mothers who have moved to a different state since they gave birth will be informed that the BFY Gift may no longer be excluded from eligibility determination for receiving social services from the new state of residence. Interviewer will record whether or not the mother decided to continue or to stop receiving the gift. Interviewers will refer participants to obtain further information and support from Lauren Mayer, Baby's First Years Project Director at Teachers College Columbia University, at (608) 291-7359 or via email at 4mybabycard@gmail.com.

Incarcerated or Institutionalized Participants

Participants who are incarcerated or institutionalized will not be asked to participate in the Age 1 visit. Interviewers will attempt to determine how long the incarceration or institutionalization may last to determine whether the mother should be re-contacted at a later time during Age 1 or in the future waves.

Child Status

Mothers who are permanently separated from the recruited child (through foster care, adoption, or other reasons) will not be asked to participate in the Age 1 visit.

Deceased Participants

If the recruited child passed away, participants will not be asked to participate any further. Interviewer may attempt to determine when death occurred. Families of mothers who are deceased will not be contacted and their BFY Gift will cease to be issued to the deceased participant's debit card.

Age 1 Visit Protocol [NOTE: The Covid-19 pandemic emerged part-way through the Age-1 wave of data collection. All in-person data collection was paused at that time, and data collection was limited to phone surveys for the remainder of the wave.]

Participants can choose the time and place of Age 1 visits. U-M SRC interviewers will be prepared to conduct the Age 1 visit at participants' home or any other location preferred by the respondent. Approximately within 24 hours before the visit, interviewers will remind participants about the upcoming interview via a phone call, email, or a text message (see: BFY_Text Message Protocol.doc) depending on participant's preference. At the time of appointment, a U-M SRC interviewer and an assistant will arrive in person at participant's home or any other location specified as a convenient place to conduct the visit.

Assistants

On some visits, U-M SRC interviewer may be aided by an assistant. Assistants' role is to help manage the distractions during administration of the protocol primarily to reduce respondent burden, reduce time spent at respondents' home, and to help the interviewer carry out data collection tasks. Assistants may use toys and tablets to distract the baby or their siblings. They may also help interviewer set up and take-down equipment for the Age 1 visit components. Assistants will not perform any data collection functions. All data collection activities are carried out by the U-M SRC interviewer.

Assistants are hired by local site PIs and managed by an Assistant Coordinator at each site. The Site Coordinator arranges for assistants to be available to help at each home visit scheduled, when possible. There is one coordinator at each of the four study sites. The coordinator is the liaison between interviewers and assistants and manages the assistants' schedules, accommodating home visit schedule changes and communicating cancellations to assistants.

Age 1 Intro and Consent

Interviewers will begin the visit by confirming that a participant has received the advance information and were able to cash the prepayment check. Interviewers will provide participant with a cash payment if they were not able to cash the check.

Interviewers will begin the visit by confirming some of the data obtained during recruitment (child's date of birth, etc.) and by confirming that the child is well, with the mother and available to participate in the visit components (Section A of the BFY Age 1 Instrument).

Interviewers will proceed to explain the components of Age 1 visit and will obtain a signature on a digital Age 1 Research Consent (see: BFY Age 1_ResearchConsent). A paper copy of the Age 1 Research Consent will be provided to each participant. All Age 1 procedures will be explained in lay language, and participants will have the opportunity to have any questions answered before signing. Interviewers will offer to read the full consent form, if needed. Participants will have the opportunity to read it themselves. It will be made clear that if a participant no longer wishes to partake in the study, she can withdraw participation at any time with no consequences.

Instrument B Consent

Participants who will not be asked questions about their children and will not be asked about child-specific parts of the visit will be presented with an alternative consent form (see: BFY Age 1_ResearchConsent_B). See below for more information about instrument B.

Instrument A and Instrument B

The age 1 instrument includes two versions, A and B. Version A includes all questions for the mother as well as child-related content and activities during which the child needs to be present. Version B excludes child-related content. In rare situations, it may become obvious that mother has been separated from the child either on a temporary basis or permanently. If a child lives with adoptive parents, the interview will not be conducted at all. If child lives with a non-familial foster parent, participants will be asked an abridged version of the instrument which includes only questions intended for the mother and excludes all child-related content. If mother indicates that it won't be possible for her child to ever be available to be interviewed along with her (question: ChildNotAtHome), the interviewer will administer the abridged instrument as well.

Voluntary Statement and Consent to Record

Following standard U-M SRC procedures, the interviews will be recorded for quality control purposes only. Recordings are encrypted and are transmitted to SRC home offices via secure FTP. Recordings are not included in the list of project deliverables sent to the University of Wisconsin. They will be securely destroyed by SRC at the end of the study. The following statement will be read to all respondents before the interview: "Before we begin, I would like you to know that this interview is completely voluntary and the information you provide is confidential. If we should come to any question you do not want to answer, please tell me and we'll go on to the next question." Respondents are also read a statement about recording and have the option to opt-out "Parts of this interview may be recorded for quality control purposes only. If you do not wish to be recorded, please let me know. You can still participate in the interview." If the respondent does not want to be recorded, the interviewer turns off the recording.

Interview flow and modules

The Age 1 instrument (see: BFY_Age 1 Instrument) has been designed anticipating that, during the visit, a child may become irritated, may need to take a nap or may be otherwise not available for parts of the interview. For that reason, the Age 1 instrument consists of modules which can be administered in varying order depending on child availability. Modules can be accessed independently and contain either self-stated questions intended only for the mother or questions and components that involve child or parent-child interaction. Depending on child availability, interviewers can choose the order in which modules are administered. Interviewers are instructed to start the interview by administering a Module: "Intro" including Section A (Intro), B (Mothers' Demographics) and C (Household Roster). Interviewers should then proceed to a Module: "Priority" including the key self-stated measures in Sections G (A Year in Review), M (Maternal Health), K (Income and Benefits), L (Expenditures and Economic Stress). After those two modules are administered, interviewers will proceed either with the self-stated questions for the mother (if child is not available) or with the components that require child involvement (EEG, Playtime, Section R).

Age 1 instrument also includes an Audio Computer Assisted Self-Interview (ACASI) module designed to collect answers to more sensitive questions. ACASI section is answered privately by the respondent. Interviewer turns the laptop to the respondent and instructs them to either read or listen to prerecorded questions via headphones and provide answers in complete privacy.

If a developmental delay is documented on the Ages and Stages Questionnaire at any time point, we will offer the mother the option of notifying her pediatrician of the result, thereby facilitating referral to Early Intervention by the pediatrician if the pediatrician feels it is warranted.

Randomizing the timing of the "Maternal happiness and agency/optimism (HOPE)" section – a BFY sub-experiment

In the proposed survey instrument, we plan to randomize the sequence of the "Maternal happiness and agency/optimism (HOPE)" section in relation to the "Income and Receipt of Public Program Benefits" section (see: BFY_Age 1 Instrument). The HOPE section will be randomized so that 50% of participants respond to the section prior to the Income section, and the other 50% respond to the section after the Income section.

The intention of the proposed randomization is to understand if cuing thoughts about income/money issues through survey questions induces variance in the way that participants' answer questions about their happiness and optimism.

Age 1 Electroencephalogram (EEG)

The objective of Age 1 EEG is to measure child brain activity at age 1, which is when approximately one-third of the total study cash gift intervention will have been administered. Conducting EEG at this point, will help to understand any added impacts of the gift on children's brain function at this point in the intervention, as well as to track the trajectory of brain development between ages 1 and 3, interviewers will measure brain function using EEG (electroencephalography).

If the baby is awake and available, interviewers can begin the Module: “EEG.” Interviewers will begin by explaining the process to the mother by showing her a brief video (see: EEG_Informational Video) and answering any questions.

BFY Age 1 EEG will be conducted with the Neuroelectric Enobio EEG acquisition system. The stretchy, infant-friendly cap is outfitted with 20 dry electrodes that are attached to a stretchy cap that fits on an infant’s head. Unlike standard electrodes, the dry electrodes do not require the application of any type of gel or saline between the electrode and the scalp. The dry electrodes can be used in scalp areas with or without hair.

The Interviewer will launch the Neuroelectric NIC2 software on their laptop and begin preparing the baby for the EEG. During this time, the interviewer or assistant may use a tablet with an engaging video or toy to keep the baby entertained and distracted (see: EEG_Video for Baby). Interviewer or assistant will put a clean neoprene cap on baby’s head. The cap will go over the head, and is secured with the chin strap. The interviewer or assistant will attach the DRL and CMS electrodes using two stickers placed below the infant’s ear.

Interviewers will verify that each electrode is settled and detecting brain function, and after that will record the EEG data with the Neuroelectric software for ~5 minutes. After recording the data, interviewers will note a few post-measurement observations to help researchers understand the context of the EEG (level of noise during the measurement, level of movement, potential interference from electronic devices nearby, etc.).

EEG data is saved on a password-protected laptop in an encrypted location. Once collected data is offloaded via a secure VPN and deposited on U-M servers. Data is then sent to the University of Wisconsin via a secure FTP connection and stored in the BFY Data Repository maintained by the University of Wisconsin.

Age 1 Parent-Child Interaction and LENA

The objective of parent-child interaction is to record 10 minutes of stimulated interaction on video and with the LENA device.

Following the instrument script in Section PT: “Playtime” (see:BFY_Age1_Playtime), interviewer will introduce this component and tell the mother that the interaction will be videotaped using a video camera connected to the laptop and confirm that this would be the right time to conduct this interaction. If participant does not want to be videotaped or refuses for another reason, this component will not be conducted. If timing is not appropriate and participant would like to conduct this part of the visit later, interviewer will select another module to complete and then return to Section PT: “Playtime” later.

Interviewer will provide 3 toys and a blanket to facilitate the parent-child interaction. Toys will be sterilized and the blanket will be cleaned after each use (or a new blanket will be provided). Participants will be asked to play with their babies for 10 minutes, using one toy after another for about 3 – 4 minutes. During the 10 minutes of playtime they will be videotaped.

Parent-child interaction video recording is saved on a password-protected laptop in an encrypted location. Collected data will be offloaded via a secure VPN and deposited on U-M servers. Data is then sent to the University of Wisconsin via a Secure FTP connection and stored in the BFY Data Repository maintained by the University of Wisconsin.

LENA Recording

Before the parent-child interaction begins, the interviewer will request that the child wears a small, child-safe recorder worn in a comfortable t-shirt. The recorder "meets U.S. and international safety standards for electronics and toys. Unlike a cell phone, it does not transmit, and it uses the same type of low-power processors as hearing aids." (source: https://www.lena.org/technology/). Approximately 10 minutes of voice recording is translated and processed using automatic Language Environment Analysis (LENA) algorithms "in the cloud" to produce the word counts, turn counts, and other information about the recording.

LENA recordings are identified by participant ID and no personally identifiable information is shared with LENA. Once recording is completed offline, interviewer uses LENA processing software to extract the files and process locally. The recording is sent "to the cloud" to apply algorithm and return the file to the local interviewer laptop. After processing, the audio is automatically deleted from LENA server as soon as analysis completes. LENA does not listen to or keep participants' recordings. Once recordings are processed and stored on interviewer laptop they are offloaded via a secure VPN and deposited on U-M servers. Data is then sent to the University of Wisconsin via a Secure FTP connection and stored in the BFY Data Repository maintained by the University of Wisconsin.

Age 1 Hair Cortisol Collection

Hair is collected on the visit to measure maternal cortisol levels as a biomarker of stress and anxiety. Interviewers will ask for participants' permission to take a very small amount of hair from the back of participant's head; no more than the thickness of pencil lead.

All interviewers will follow the same scripted instructions displayed on the computer (see: BFY_Age 1 Instrument, Section S). Interviewers will:

- Put on gloves
- Use a comb to partition the hair between the ears on the back of the head below the midline.
- Fasten the hair above the ears out of the way with plastic or aluminum hair clip(s).
- Isolate a small bundle of hair that is approximately the size of a pencil lead (3 mm in diameter.)
- Tie the string around this small bundle of hair close (0.25 cm – 0.5cm) from the root.
- Cut hair as close as possible to the scalp (between the scalp and the string).
- Place the sample in the aluminum foil, and fold the foil over the sample.
- Mark the foil with an arrow, the arrow head indicating the end of hair closest to the scalp (i.e. where the string is)
- Place the hair and the sample authorization form in a zip-lock bag.
- Unclip hair, and sanitize hands with rubbing alcohol

Hair collection will not take place for participants whose hair is shorter than three centimeters.

Collected samples will be mailed in a pre-stamped and pre-addressed envelope to Teachers College Columbia University. Samples will be identified only by a study ID and will not contain any identifiable information.

Age 1 Picture Souvenir

Participants will have the option of receiving a picture of themselves and their baby once the visit is complete. Interviewers will use a Polaroid camera to take this picture and attach it to a study specific postcard labeled "Baby's First Years."

The objective of the picture souvenir is to provide the participant with another token of appreciation of their time, as well as a reminder of the study visit.

Age 1 Administrative Records and Transaction Tracking Consent

Upon recruitment, participants were asked to provide consent to track 4MyBaby debit card transactions and to provide access to administrative data on social services obtained.

At the Age 1 visits, participants who did not provide consent for either of these will be told again about the research objectives associated with both sources of data and their importance and asked if they would reconsider providing consent to obtain Administrative Data and for consent to track their debit card spending.

Respondents who provided Administrative Consent but their Social Security Number was invalid will be asked to update their SSN. SSN is immediately encrypted upon entering in the survey instrument and can only be decrypted once transmitted to the secure data enclave.

Nebraska Administrative Records Consent

Nebraska's Department of Health and Human Services (DHHS) requires that their *Authorization for Disclosure of Protected Health Information* form be completed in order to release administrative records of participants. Accordingly, Nebraska participants will be asked for consent using this form (see: Authorization for Disclosure of Protected Health Information). The form will be completed in hard-copy and will be mailed to the University of Michigan, then scanned and deposited on U-M servers. Data is then sent to the University of Wisconsin via a secure FTP connection and stored in the BFY Data Repository maintained by the University of Wisconsin.

Nebraska participants will also be asked to provide their SSN electronically, for administrative records that are not housed at DHHS.

Since Dr. Katherine Magnuson is listed as the recipient of the data authorized by participants, interviewers will provide the following information if asked exactly where the data is going and who will be receiving and using the data:

"*Katherine is one of the BFY researchers, and is a professor of social work at the University of Wisconsin. We'd like to know about the social services and benefits that your family may be receiving, to understand more about your day-to-day life. Katherine is in charge of getting this information from Nebraska's Department of Health and Human Services, and keeping it safe and confidential. DHHS will not know that you're getting the gift money, unless you already provided consent for that to happen."*

Home Observations

At the end of the visit, interviewer will fill out short series of observations (Section V: "Observations") about the site of the interview including where did the interview take place (e.g., at home or out-of-home, which room, etc.), whether the place appeared safe for the child, whether they saw evidence of pests, whether they saw any books, whether anyone else was present during the interview, and whether the mother revealed or interviewer otherwise found out about the amount of cash gift the mother is receiving from the study.

4MyBaby card replacement

BFY cash gift is deposited on a debit card which expires 3 years from issuance. U-M SRC interviewers will replace ClinCards provided to mothers at the time of recruitment with new cards at the time of the Age 1 and Age 2 visits to ensure each card lasts a full 42 months.

Monitoring cooperation and increasing response rates:

Monitoring production will be important to ensure visits are completed within the desired 2-week window before/after the first birthday. We anticipate that some participants may take longer to respond to our interview request and may voice concerns with participation. We plan to implement a few strategies to ensure participation within the specified time. Those strategies may include:

- Understanding and addressing participants concerns;
- Offering to break up one longer visit into shorter visits;
- Offering more flexible choices of dates and time for conducting the visit;
- Utilizing assistants to reduce respondent burden by
    - Accelerating administration of the protocol (assistants will help interviewer move through the protocol quickly and reduce total amount of time spent in participants' homes)
    - Helping to manage distractions (e.g., assistants will help keep siblings entertained during the protocol administration);
- Monitoring production and implementing initiatives to boost on-time participation
    - U-M will monitor the number of successful / unsuccessful attempts to gain cooperation and record specific concerns raised by participants.
    - Non-respondents may receive additional mailings, emails, or text messages addressing their concern and offering appropriate alternatives.
    - In some cases, we may increase the token of appreciation for participating in BFY from $100 up to $200. There will be two cases in which the token of appreciation could be increased to $200, listed below. Participants receive $100 as base, and some participants could receive either one or both of the $50 incentives listed below, if they qualify. This outline will be offered equally to all participants.
        - Incentive for keeping appointments
            - Amount: $50

    - Eligibility: Respondents who reschedule or cancel appointment for the 2nd time or respondents who have had one no show.
    - How will it be offered? Interviewer will make a one-time offer of $50 during the next contact attempt for keeping their next appointment. Offer will be made via phone, email, or a text message.
  - Age 1 End Game Incentive
    - Amount: $50
    - Eligibility: Respondents whose child is 15 months or older
    - How offered? Interviewer (phone, email, text) or paper mailing.

Reports of Child Abuse/Endangerment

BFY plans to report evidence of suspected child abuse, neglect or endangerment, corresponding to statutes in the study sites included in the research, if observed by an interviewer. Although interviewers will not ask any interview questions or make any standardized observations which meet the definition of criminal child abuse, neglect or endangerment, interviewers may very rarely overhear or observe something in the respondent's home which requires a mandated report. Interviewers who are mandated reporters in the state in which the interview is conducted will file reports directly with the state, and are also required to notify SRC if a report has been filed. Interviewers who are not considered to be mandated reporters will be instructed to contact a licensed clinician employed by SRC, and SRC will work with the interviewer to complete any necessary mandated reporting requirements.

Counseling or Support for Study Participants

All adult respondents will be given a list of national Hotline numbers prior to the start of an interview.
The SRC has a referral system in place whereby any respondent requesting or, in the judgment of the non-clinician interviewer, requiring further assistance is referred by interviewers to a licensed social worker with mental health experience. The Baby's First Years respondents and interviewers will be supported by the University of Michigan Survey Research Center's Clinical Contact Protocol team (CCP). The CCP team consists of licensed social workers with mental health experience, who have been hired to support the Survey Research Center's interviewing efforts.

The CCP members take referrals from interviewers. Upon receipt of an email or phone request, the CCP will follow up directly with respondents (and interviewers) and will provide confidential referrals for local support services. Typically, the support to respondents includes referrals to clinical, mental health, and other related services in the local area, though sometimes the CCP member simply talks directly with the caller and no further services are necessary. The social worker will contact the individual by telephone, assess the needs of the respondent(s), and connect the respondent with local services. Interviewers engage the CCP through a request in a secure web portal.

Interviewers are not tasked or required to provide counseling assistance to respondents, other than to take information for a referral to a CCP, or refer to numbers from the Hotline List when

requested to do so by the respondent. The CCP will assess the respondent's situation and make appropriate referrals.

Age 2 Data Collection Protocol **[NOTE: As noted below, we had planned for flexible data collection during the age-2 wave, depending on the status of the Covid-19 pandemic and associated restrictions. In reality, no in-person data described below were collected during this wave.]**

Overview

In Baby's First Years, Age 2 participants recruited in the Baseline will be asked to participate in the 2nd year home visit scheduled within two to four weeks before or after their child's second birthday. Just like Age 1 visits, the Age 2 visit will be scheduled and conducted by a University of Michigan Survey Research Center (U-M SRC) interviewer.

Due to circumstances related to Covid-19 and restrictions on in-person visits, we have planned a flexible survey administration to allow for both phone and in-person data collection. When in-person work is restricted or participant refuses in-person data collection but agrees to a phone-based interview, we plan to administer the first (Main) portion of the Age 2 interview via phone (CATI, computer-assisted telephone interview). The second portion of the Age 2 interview, including in-person (CAPI, computer-assisted personal interview) Activities, will be conducted after restrictions are lifted. Respondents who complete the first, Main part of the interview via phone will be asked to participate in the in-person Activities. Respondents contacted after restrictions are lifted will be able to complete both the Main and Activities portions of the interview, during one in-person visit. However, to reduce respondent burden, in some cases we may still choose to break up the interview into two parts and conduct the Main interview over the phone. Further description of the content of the Main and the Activities interviews are included later in this protocol.

Before the Main interview, all families will be sent brief letters with information about upcoming Age 2 activities as well as a pre-payment check of $50. Interviewers will follow up with phone calls (or emails and text messages if more appropriate) to arrange for the best time to conduct the phone interview and/or the visit. The Main part of the interview is estimated to last between 60 and 75 minutes and the Activities portion is expected to take another 30 – 45 minutes, resulting in a combined interview time of 90-120 minutes. At the beginning of the Main interview, the interviewer will present the consent, give participants an opportunity to ask any questions, and ask for a digital signature before the interview begins.

Participants who did not or could not cash the pre-payment check will receive $50 in cash in appreciation for their time. After completing the in-person Activities, respondents will receive $50 in cash for a total of $100 for the Age 2 visit. If Covid-19 conditions do not allow for an in-person visit around the child's birthday, respondents will receive the $50 pre-payment check, $50 for completing the phone interview and $50 for a later in-person follow-up visit, should conditions allow for it at another time.

Age 2 Visit Protocol

Eligibility

We will conduct Age 2 interviews with participants who consented to participate in the Baby's First Years study during the Baseline interview and accepted the BFY Gift during the baseline hospital visit.

Participation in the Age 1 interview is not a necessary condition to participate in Age 2. We will invite all participants who may or may not have participated in the Age 1 visit.

We will NOT conduct Age 2 visits with participants whose recruited child is deceased.

If a participant asked to withdraw from the study during Age 1, they may be contacted again in Age 2 to confirm their withdrawal and to determine the reasons for withdrawal. However, they will not be contacted for the purpose of conducting the Age 2 interview unless they re-consider and decide to continue participation. Participants who withdraw from the study will continue receiving the BFY Gift.

Sample Release and Pre-visit Activities

We aim to complete Age 2 visits within 4 weeks before or after the 2nd birthday of the recruited child although some participants may end up being interviewed earlier or later than planned. As in Age 1, the U-M data collection team will send an advance mailing inviting all Moms to participate in the Age 2 visit. The mailing will be sent approximately 4 – 8 weeks before the second birthday of the recruited child. Approximately, 2 – 6 weeks before the second birthday sample will be released to interviewers and they will start contacting participants via phone (or email and text messages as appropriate) and scheduling visit appointments.

The advance mailing will include:

a) An introductory letter thanking participants for being part of the study and reminding them they will be contacted again soon (see: BFY_Age2PrecontactLetter).
c) A prepayment check of $50
d) A copy of the Age 2 Research Consent.
e) The Age 2 Main instrument Respondent Booklet including answer scales to the instrument question which will facilitate and speed up completing of the Age 2 Main instrument over the phone.

Participants who provided an email address may additionally be sent advance information via email (see: BFY Email_Protocol). Participants who provided a cell phone number may also receive a short text message sent by the interviewer once the sample is released (see: BFYText Message Protocol).

Interviewer Contact Protocol
Once sample is released, the interviewer will attempt to reach participants using contact information obtained during Baseline and Age 1 data collection. Interviewers will begin by calling available phone numbers on various days and times of day such as weekday morning, afternoon, or

evening, as well as weekend morning, afternoon or evening. If U-M interviewer reaches an answering machine/voicemail response, they will leave a scripted message, which does not reveal the study name (see: BFY_VoicemailScript). Interviewer will leave their name and a phone number and ask for a call back to either the interviewer's work number or the U-M toll–free number.

If participants cannot be reached using the available phone numbers, interviewers may attempt to go in person to the address provided during recruitment. Interviewers may leave "Sorry I Missed You" (SIMY) cards with interviewer work number or the U-M SRC's toll free number (see: BFY-SIMY_Card.doc). In-person visits will only be conducted if permissible under Covid-19 state,local, and IRB regulations. Interviewer may also refer hard-to-reach participants to a specialized U-M SRC locating team. The locating team calls available numbers and uses directory assistance to obtain additional information. The locating team may also conduct internet searches to obtain updated address or telephone numbers. Additionally, at the time of recruitment, some participants provided alternative contact information including phone, email, or address of the best person to contact if the participants are hard to find. Interviewers and/or the locating team may reach out to designated contact person(s) asking for help in reaching the participant. They may also ask contact person(s) to let the respondent know we are trying to reach them and ask them to contact the U-M SRC toll-free number. In some cases, we may offer a small finder's fee of $10 to $20 in appreciation for assistance in locating a BFY participant.

Depending on available contact information, interviewers may also try to reach participants via email or text messages. Email and text messages may also be used to remind respondents about upcoming appointments. (see: BFY_Email_Protocol.doc and BFY_Text Message Protocol.doc)

Upon reaching participants, interviewers will confirm whether a participant received an advance letter with BFY Age 2 visit information and whether they have any further questions. Interviewers will respond to any questions and setup an appointment to conduct the Age 2 phone or in-person interview.

Unexpected situations

During pre-visit contact, interviewer may come across information that will require a specific course of action or to provide Mom with more information about her participation in the BFY study. Participants who cannot take part in the Age 2 visit will continue to receive the BFY Gift unless they specifically opt-out and choose not to receive cash payments.

State Residence and Eligibility for Social Services

Prior to scheduling a visit, interviewers will confirm a participant's current address. If Mom moved out of the recruitment state she will be informed that she can continue to participate in the study and continue receiving the BFY Gift. However, she will also be informed that the BFY Gift may no longer be excluded from eligibility determination for receiving social services from the new state of residence. Interviewer will record whether or not Mom decided to continue or to stop receiving the gift and whether or not she is interested in further participation in the study. Interviewers will refer participants to obtain further information and support from Lauren Mayer, Baby's First Years Project Director at Teachers College Columbia University, at (608) 291-7359 or via email at 4mybabycard@gmail.com.

Incarcerated or Institutionalized Participants

Participants who are currently incarcerated or institutionalized will not be asked to participate in the Age2 visit. Interviewers will attempt to determine how long the incarceration or institutionalization may last to determine whether Mom's release date would allow us to interview her during Age 2 data collection or whether she should be re-contacted in the future waves.

Child Status

Mothers who are permanently separated from the recruited child (through foster care, adoption, or other reasons) will be asked to participate in a modified version of the Age 2 visit. The modified version of the visit will exclude any questions about the target child, focusing instead on the experiences of the mother.

Deceased Participants

If the recruited child passed away after the Age 1 visit was completed, participants will not be asked to participate any further. Interviewer may attempt to determine when death occurred. Families of mothers who are deceased will not be contacted and their BFY Gift will cease to be issued to the participant's debit card.

Age 2 Visit

Participants can choose the time and place of Age 2 interviews. U-M SRC interviewers will be prepared to conduct the Age 2 interviews via phone, at participants' home, or any other location preferred by the respondent. Approximately 24 hours before the interview, interviewers will remind participant about the upcoming appointment via a phone call, email, or a text message (see: BFY_Text Message Protocol.doc) depending on participant's preference. If the interview is conducted in person, at the time of appointment, a U-M SRC interviewer and an assistant will arrive at participant's home or any other location specified as a convenient place to conduct the visit.

Assistants

Similar to Age 1, during Age 2 in-person visits, U-M SRC interviewer may be aided by an assistant. The assistants' role is to help manage the distractions during administration of the protocol primarily to reduce respondent burden, reduce time spent at respondents' home, and to help the interviewer carry out data collection tasks. Assistants may use toys and tablets to distract the baby or their siblings. They may also help interviewer set up and take-down equipment for the Age 2 visit components. Assistants will not perform any data collection functions. All data collection activities are carried out by the U-M SRC interviewer.

Assistants are hired by local site PIs and managed by an Assistant Coordinator at each site. The Site Coordinator arranges for assistants to be available to help at each home visit scheduled, when possible. There is one coordinator at each of the four study sites. The coordinator is the liaison between interviewers and assistants and manages the assistants' schedules, accommodating home visit schedule changes and communicating cancellations.

Age 2 Intro and Consent

Interviewers will begin the interview by confirming that the participant has received the advance information and were able to cash the prepayment check. Interviewers will either provide participant with a cash payment if interview is conducted in person or request another check to be mailed if the respondent did not receive or were not able to cash the check.

Interviewers will begin the visit by confirming some of the data obtained during recruitment and the Age 1 interview (child's date of birth, etc.) and by confirming that the child is well, with Mom, and available to participate in the Age 2 Main and Activities portions of the interview (Section A of the BFY Age 1 Instrument).

Interviewers will proceed to explain components of the Age 2 Main and Activities instrument as described in the Research Consent. All Age 2 procedures will be explained in lay language, and participants will have the opportunity to have any questions answered before signing. Interviewers will offer to read the full consent form, if needed. Participants will have the opportunity to read it themselves. It will be made clear that if a participant no longer wishes to partake in the study, she can withdraw participation at any time with no consequences. If the Main interview is conducted via phone, interviewer will date and sign electronic consent as witnesses and write-in that consent was conducted via phone in lieu of respondent signature. If the Main interview is conducted in person, interviewers will obtain respondent signature on a digital Age 2 Research Consent (see: BFY_Age2ConsentA). A paper copy of the Age 2 Research Consent will be provided to each participant in the advance mailing or during the in-person visit.

Instrument A and Instrument B

Age 2 instrument includes two versions, A and B. Version A includes all questions for the mother as well as child-related content and activities during which the child needs to be present. Version B excludes child-related content. In rare situations, it may become obvious that mother has been separated from the child either on a temporary basis or permanently. If a child lives with adoptive parents, the interview will not be conducted at all. If child lives with a foster parent, participants will be asked an abridged version of the instrument which includes only questions intended for the mother and excludes all child-related content. If mother does not think her child will be available to be interviewed along with her, the interviewer will administer the abridged instrument as well.

Voluntary Statement and Consent to Record

Following standard U-M SRC procedures, the interviews will be recorded for quality control purposes only. Recordings are encrypted and are transmitted to SRC home offices via secure FTP. Recordings are not included in the list of project deliverables sent to the University of Wisconsin. They will be securely destroyed by SRC at the end of the study. The following statement will be read to all respondents before the interview: "Before we begin, I would like you to know that this interview is completely voluntary and the information you provide is confidential. If we should come to any question you do not want to answer, please tell me and we'll go on to the next question." Respondents are also read a statement about recording and have the option to opt-out "Parts of this interview may be recorded for quality control purposes only. If you do not wish to be

recorded, please let me know. You can still participate in the interview." If the respondent does not want to be recorded, the interviewer turns off the recording.

Interview flow and modules

The Age 2 instrument has been designed anticipating that some restrictions on in-person visits due to Covid-19 will persist during the first few weeks of Age 2 data collection. As such, the instrument has been separated into two parts:

1) The Main interview which can be administered both via phone (CATI) and in-person (CAPI).
2) The Activities instrument which can only be administered in-person.
3) An Administrative instrument, asked only of respondents who did not provide the information during Baseline and Age 1 visits.

Age 2 Main Instrument (CATI or CAPI)

Age 2 Main instrument can be conducted via phone and in-person as the conditions allow. Interviewers are instructed to always start the Age 2 data collection with the Main interview and by administering a Module: "Intro" including Section A (Intro), B (Mothers' Demographics) and C (Household Roster). Interviewers should then proceed to complete the Main instrument including Sections C – T. Once restrictions on in-person visits are lifted interviewers can administer the Main interview in person and also complete the in-person Activities instrument to complete the Age 2 protocol.

Age 2 Main instrument will include a module with potentially sensitive questions. This section will be included in both the Main and Activities instrument. It will be administered either via phone, if there are restrictions on in-person work, or via Audio Computer Assisted Interview in the in-person Activities instrument once in-person work is no longer limited. The ACASI section is answered privately by the respondent. Interviewer turns the laptop to the respondent and instructs them to either read or listen to prerecorded questions via headphones and provide answers in complete privacy.

Age 2 Activities Instrument

The Age 2 Activities instrument can be conducted in-person only and will include the following sections listed below.

Age 2 Activities: MCDI:

In Age 2 Baby's First Years (BFY) is administering MacArthur-Bates Communicative Development Inventories (MacArthur-Bates CDI) which is a short syndicated instrument, which assesses children's language development. Traditionally, MacArthur-Bates CDI is administered in-person. Parents / guardians are read specific instructions by the experimenter and asked about "words that your child 'understands and says.' Parents/guardians then check the box "yes/no" for any word that their child understands and says.

Per administration guidelines, Age 2 BFY prepared a digital in-person form of the MacArthur-Bates CDI, however, the form could not be used due to limitations imposed by COVID-19. To adapt, BFY has prepared a short online survey in Qualtrics, which can be filled out by the mother on any device and submitted after the main Age 2 BFY Interview. The BFY Qualtrics M-B CDI includes a Spanish and an English version of the MacArthur-Bates CDI instrument which are displayed based on the language child hears at home. Mothers of children exposed to both languages are asked to assess which words in both Spanish and English the child understands and says. The full version of the BFY Qualtrics MacArthur-Bates CDI Survey is included in the attached document (BFY Qualtrics MacArthur-Bates CDI Survey.pdf).

BFY Age 2 MacArthur-Bates CDI is administered in the following way:

- Prior to the interview, interviewers typically call, text, or email the mother to confirm her interview appointment. During that contact, interviewers also provide mothers the link to the short M-B CDI survey. Interviewers will ask participants to enter limited personal information about their child to log into their unique MCDI online survey via Qualtrics. The following question is added to the survey:

  "Just to confirm for our records, what is your child's first name initial and the month and date of their birth?

  First Name Initial:
  Month day of birth (MM/DD):"
- During the main Age 2 Interview, the interviewer reads a set of instructions for how to complete the MacArthur-Bates CDI, answers any questions, and confirms that mother received the link and can access the survey. The Interviewer also asks for mom to complete the survey right after the main interview.
- Following the Main Age 2 interview, if the MacArthur-Bates CDI survey is not completed within 24 hours, interviewers place a courtesy reminder via call, email, or text message to confirm mother can access the survey, to troubleshoot any problems, and to thank mother for her willingness to complete the survey. If the survey is not completed within 1 week following the Main Age 2 interview, interviewers will send one more single text message, email or call (leaving a VM if mother is not available) to remind mothers of the importance of completing the survey and to thank them for their participation in this component of Age 2 BFY.

Age 2 Activities: Flanker

During the in-person Age 2 visit, BFY interviewers will administer the NIH Toolbox version of Flanker task.

"The NIH Toolbox Flanker Inhibitory Control and Attention Test (Flanker) measures both a participant's attention and inhibitory control. The test requires the participant to focus on a given stimulus while inhibiting attention to stimuli (fish for ages 3-7 or arrows for ages 8-85) flanking it."

http://www.healthmeasures.net/images/nihtoolbox/Technical_Manuals/Cognition/Toolbox_Flanker_Inhibitory_Control_and_Attention_Test_Technical_Manual.pdf

The standardized, app-based version is easy to administer, and the task itself should only lasts 3 minutes. Flanker will be administered on an iPad. Interviewers will setup the tablet, enter information on app-based questions about mothers age and education level and hand the device over to Mom to follow on-screen instructions. Participants' data are identified only by their BFY sample ID and no other information is entered into the app. The data download is conducted after the visit by the interviewers. Data from the Flanker app is sent to the protected access point within the SRC U-M servers.

Age 2 Activities: Parent-Child Interaction

The objective of parent-child interaction is to record 10 minutes of play-based interaction.

Following the instrument script in Section PT: "Playtime", interviewer will introduce this component and tell Mom that the interaction will be videotaped using a video camera connected to the laptop and confirm that this would be the right time to conduct this interaction. If participant does not want to be videotaped or refuses for another reason, this component will not be conducted. If timing is not appropriate and participant would like to conduct this part of the visit later, interviewer will select another module to complete and then return to Section PT: "Playtime" later.

Interviewer will provide 3 toys in separate bags? and a blanket to facilitate the parent-child interaction. Toys will be sterilized after each use. Participants will be asked to play with their child for 10 minutes, using one toy after another for about 3 – 4 minutes. During the 10 minutes of playtime they will be videotaped.

Parent-child interaction video recording is saved on a password-protected laptop in an encrypted location. Collected data will be offloaded via a secure VPN and deposited on U-M servers. Data is then sent to the University of Wisconsin via a Secure FTP connection and stored in the BFY Data Repository maintained by the University of Wisconsin.

Age 2 Activities: LENA Recording

During parent-child interaction, the child will be asked to wear a small, child-safe recorder worn in a comfortable vest. The recorder "meets U.S. and international safety standards for electronics and toys. Unlike a cell phone, it does not transmit, and it uses the same type of low-power processors as hearing aids." (source: https://www.lena.org/technology/). Approximately 10 minutes of voice recording is translated and processed using automatic Language ENvironment Analysis (LENA) algorithms "in the cloud" to produce the word counts, turn counts, and other information about the recording.

LENA recordings are identified by participant ID and no personally identifiable information is shared with LENA. Once recording is completed offline, interviewer uses LENA processing software to extract the files and process locally. The recording is sent "to the cloud" to apply algorithm and return the file to the local interviewer laptop. After processing, the audio is automatically deleted from LENA server as soon as analysis completes. LENA does not listen to or keep participants' recordings. Once recordings are processed and stored on interviewer laptop they are offloaded via a secure VPN and deposited on U-M servers. Data is then sent to the University of Wisconsin via a

Secure FTP connection and stored in the BFY Data Repository maintained by the University of Wisconsin.

Age 2 Epigenetics / Saliva Collection

During Age 2 visits interviewers will collect saliva samples from both mother and child. Saliva will be collected using kits provided by DNAGenotek (DNA Genotek Inc. www.dnagenotek.com).

Interviewers will begin by explaining the process to the mother verbally or by showing her a brief video, and answering any questions.

At the beginning of the visits, interviewers will ask participants not to eat or drink anything including water for approximately 30 minutes before collecting saliva samples. Prior to the collection, interviewers will confirm if participants ate, drank anything including water, smoked, or chewed gum within 30 minutes of donating saliva.

After collection, saliva tubes will be mailed to the TCCU for storage and batched mailings to the analysis lab at The University of Texas at Austin. Samples will be only identified by participants BFY Sample ID and no personally identifiable information will be included on the shipment.

Samples will be analyzed for epigenetic aging using the Horvath method and cellular mechanisms for aging (methylation). DNA will be extracted from the samples taken but it will be immediately treated with a chemical that alters the DNA. Because of this treatment, the DNA can't be sequenced and will not be sequenced to determine the epigenetic age or any other epigenetic measure.

Saliva collection protocol:

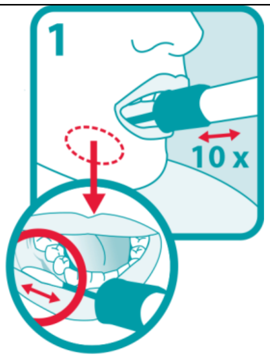	**Step 1**<br>Open DNAGenotek package with the "OPEN HERE" arrow<br>Remove collector by its handle from the packaging (ensure sponge tip does not come into contact with any surface prior to collection!)<br>Place sponge as far back in the mouth as comfortable and rub along the lower gums in a back and forth motion<br>Gently rub gums 10 times (if possible avoid rubbing teeth)<br>Tips for 10 mos and up: Tell them a little tickle will happen and try to give them a job of some sort (like holding something you'll need later or pointing to different stickers you say in the room)
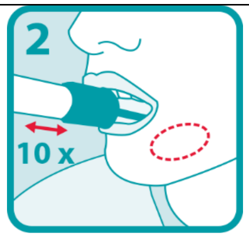	**Step 2**<br>Gently repeat rubbing motion on the opposite side of the mouth along the lower gums for an additional 10 times

TEACHERS COLLEGE
COLUMBIA UNIVERSITY
Institutional Review Board
Box 151 | 525 West 120th St. New York, NY 10027
212-678-4105 | RH 13 | IRB@tc.edu | tc.edu/IRB

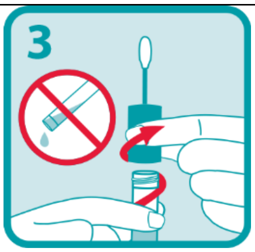	**Step 3**<br>Hold the tube upright to prevent the blue liquid inside the tube from spilling<br>Unscrew the blue cap from the collection tube without touching the **sponge**
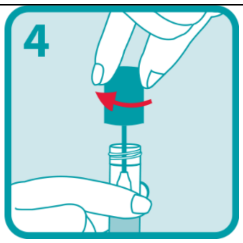	**Step 4**<br>Turn the cap upside down<br>Insert the sponge into the tube and close cap tightly
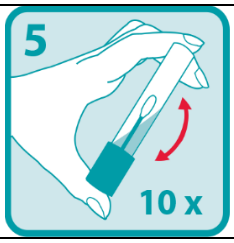	**Step 5**<br>Invert the capped tube and shake vigorously 10 times
	**Step 6**<br>Put sample in biohazard bag and seal bag closed bag by peeling off blue liner<br>Sample will be labeled with the following information:<br>Study Name: BFY<br>Participant ID<br>Parent/Child (circle one)<br>Collection date<br>Time collected and AM/PM (circle one)<br>Interviewer initials<br>Sample will be mailed to the storage at TCCU and then shipped in batches to the University of Texas at Austin for analysis.

Age 2 Administrative Consent Instrument

During the recruitment wave and again during Age 1 visit, participants were asked to provide consent to access to administrative data on social services obtained.

During Age 2 visit, participants who thus far did not provide consent will be told again about the research objectives associated with their administrative data and its importance, and asked if they would reconsider providing consent to obtain Administrative Data.

The administrative records consent will be conducted over the phone. We will not ask for social security numbers on the consent form. Interviewers will explain the administrative consent over the phone with a paraphrased script, and will include the following four things:

a. Remind participants that what is expressed verbally is also on the consent form which they should have a copy, and if not you can send them a copy.
b. Remind them the study is voluntary, they can skip any questions, or stop anytime, without penalty.
c. If they would like a copy of the verbal script you can send it to them.
d. Ask if they understand what is asked of them, if they have questions, and if they would like to continue.

Home Observations

At the end of the visit, interviewer will fill out a short series of observations (Section V: "Observations") about the site of the interview including where did the interview take place (e.g., at home or out-of-home, which room, etc.), whether the place appeared safe for the child, whether they saw evidence of pests, whether they saw any books, whether anyone else was present during the interview, and whether Mom revealed or interviewer otherwise found out about the amount of cash gift Mom is receiving from the study.

ClinCard replacement

BFY cash gift is deposited on a debit card which expires 3 years from issuance. U-M SRC interviewers have started replacement of ClinCards / 4MyBabyCards provided to Moms during Age 1 data collection. Remaining Moms will receive their replacement cards during Age 2 visits.

Monitoring cooperation and increasing response rates:

Monitoring production will be important to ensure visits are completed within the desired window before/after the first birthday. We anticipate that some participants may take longer to respond to our interview request and may voice concerns with participation. We plan to implement a few strategies to ensure participation within the specified time. Those strategies may include:

- Understanding and addressing participants concerns;
- Offering to break up one longer visit into shorter visits;
- Offering more flexible choices of dates and time for conducting the visit;
- Utilizing assistants to reduce respondent burden by
    - Accelerating administration of the protocol (assistants will help interviewer move through the protocol quickly and reduce total amount of time spent in participants' homes)
    - Helping to manage distractions (e.g., assistants will help keep siblings entertained during the protocol administration);
- Monitoring production and implementing initiatives to boost on-time participation
    - U-M will monitor the number of successful / unsuccessful attempts to gain cooperation and record specific concerns raised by participants.

- Non-respondents may receive additional mailings, emails, or text messages addressing their concern and offering appropriate alternatives.
- In some cases we may increase the token of appreciation for participating in BFY from $100 up to $200.

Reports of Child Abuse/Endangerment

As during Age 1, during Age 2 BFY plans to report evidence of suspected child abuse, neglect or endangerment, corresponding to statutes in the study sites included in the research, if observed by an interviewer. Although interviewers will not ask any interview questions or make any standardized observations which meet the definition of criminal child abuse, neglect or endangerment, interviewers may very rarely overhear or observe something in the respondent's home which requires a mandated report. Interviewers who are mandated reporters in the state in which the interview is conducted will file reports directly with the state, and are also required to notify SRC if a report has been filed. Interviewers who are not considered to be mandated reporters will be instructed to contact a licensed clinician employed by SRC, and SRC will work with the interviewer to complete any necessary mandated reporting requirements.

Counseling or Support for Study Participants

The SRC has a referral system in place whereby any respondent requesting or, in the judgment of the non-clinician interviewer, requiring further assistance is referred by interviewers to a licensed social worker with mental health experience. The Baby's First Years respondents and interviewers will be supported by the University of Michigan Survey Research Center's Clinical Contact Protocol team (CCP). The CCP team consists of licensed social workers with mental health experience, who have been hired to support the Survey Research Center's interviewing efforts.

The CCP members take referrals from interviewers. Upon receipt of an email or phone request, the CCP will follow up directly with respondents (and interviewers) and will provide confidential referrals for local support services. Typically, the support to respondents includes referrals to clinical, mental health, and other related services in the local area, though sometimes the CCP member simply talks directly with the caller and no further services are necessary. The social worker will contact the individual by telephone, assess the needs of the respondent(s), and connect the respondent with local services. Interviewers engage the CCP through a request in a secure web portal. Only the following resources will be offered by interviewers directly:

- Substance Abuse and Mental Health Services Administration (SAMHSA) National Helpline
- National Domestic Violence Hotline
- National Suicide Prevention Lifeline
- National Child Abuse Hotline
- Local Covid-19 support and information lines

Interviewers are not tasked or required to provide counseling assistance to respondents, other than to take information for a referral to a CCP, or refer to numbers from the Hotline List (including

covid-19 resource referrals) when requested to do so by the respondent. The CCP will assess the respondent's situation and make appropriate referrals.

BFY Age 2 In-Person Locating Restart

Safe in-Person Locating Protocol

We seek approval to activate limited in-person work as part of the Baby's First Years (BFY) during the COVID-19 pandemic. Specifically, would like an approval to conduct in-person locating visits according to the protocol described in this document in an effort to find respondents for whom we have exhausted prior contact information. This protocol will be carried out in all study sites Nebraska, New York, Minnesota, and Louisiana.

BFY In-Person Locating Activation plan:

Activation of limited in-person locating requires two approvals:

a. An approval from the BFY IRB of Record (TCCU IRB) to resume in-person locating activities outlined in this protocol.

b. An approval from the University of Michigan COVID-19 Research Reengagement Committee to authorize in-person work on the U-M research staff. Authorization is the subject to the following guidelines. (https://www.research.umich.edu/covid-19/research-reengagement)

   The University of Michigan Research Reengagement Committee application was approved on January 18, 2021.

Along with the receipt of approval from TCCU/NYSPI and the U-M Research Reengagement Committee, the U-M IRB is also notified but this notification is not a required step to commence the in-person locating work.

BFY Safe In-Person Locating protocol:

Guiding principles of the in-person locating protocol:

- As needed only
- Voluntary
- Minimizing exposure risk for interviewers and participants
- Complying with CDC prescribed guidelines, protective equipment, procedures
- Preceded by U-M training on Covid-19 research procedures

As needed only

The BFY Age 2 project will resume in-person locating during the COVID pandemic in order to help find respondents that have been unreachable for their BFY Age 2 interview. The project has exhausted all current methods of locating some respondents (R) e.g., calling, texting, emailing,

database searches. Our last resort to keep the respondents in the study is to complete an in-person locating visit in an attempt to reach the respondent. The main goals of the visit are to:

1. Visit the best last-known address on file for the respondent
2. Speak with the respondent (if available) or the informant who opens the door
3. Obtain updated contact information for participants (address, cell phone, emails, or Contact Persons);
4. Schedule an appointment for the Age 2 phone Interview (if speaking with the respondent)
5. In some cases, interviewers may also deliver post-interview payments in cash to families who do not use banking services and may experience difficulties processing mailed payments via check.

Voluntary

The locating effort will be voluntary and interviewers will be allowed to self-select for this effort. Interviewers who do not volunteer will be held harmless in terms of project assignment.

Minimizing Covid-19 Risk to Participants and Interviewers

The locating protocol will minimize the exposure risk by mandating social distancing and mask wearing.

Protection for respondents:

All visits will be conducted outside of the respondent/informant households. Interviewers will NOT enter respondent's or informant's homes during this effort. Exceptions may include apartment building lobbies, foyers, hallways. However, interviewers may individually assess whether it would be safe to enter any of the apartments / public housing units and choose not to conduct work if it were to violate the social distancing guidelines. If the interviewer determines that there is no way to safely distance they may omit to work that area or building.

Interviewers will be trained on and must follow all State regulations and the CDC guidelines while working on this effort. Interviewers will wear a mask at all times and will be required to stay six feet away from the door when it opens and maintain a distance of six feet apart from respondents and informants during the conversation.

Interviewers will be required to limit interactions with any given person to less than 15 minutes to meet current guidelines on minimizing exposure.

Interviewers will conduct daily University of Michigan ResponsiBlue health screening and will not work on the locating task if they exhibit any of the Covid-19 symptoms.

Protection for interviewers:

Interviewers will be asked to determine whether the respondent/informant poses any Covid-19 risk. While wearing a mask and positioned at least six feet away from the door, the interviewer will introduce themselves briefly, and then ask necessary minimum questions to establish whether anyone in the household may be experiencing or has been exposed to Covid-19 symptoms. Here is the proposed script for the interviewer:

"Hi, my name is ... and I am from University of Michigan. We are looking for ... who is participating in one of our studies." "Before we continue, just to be on the safe side, are you currently experiencing or have you been exposed to anyone who has any symptoms of Covid-19?"

If the informant responds "yes", the interviewer will thank and terminate the visit. A log with this information will be entered in the case notes.

Protective Equipment

The project will provide interviewers with the essential equipment needed to protect themselves in the field for the locating effort. Each interviewer will receive:

- hand sanitizer
- sanitizing wipes
- 1 or 2 cloth masks
- A supply of disposable masks (a larger quantity to suffice for at least 1 mask per trip)

Training
Interviewers will complete a required University of Michigan Human Research During COVID-19 Training Module and attestation.

A copy of the training is available for review here:

Human Research During Covid (Copy)

Interviewers will be also instructed by the BFY Management Team on use and sanitation of equipment used during in-person visits. Interviewers will also be trained on the appropriate protocol if they encounter a respondent or informant who either self-reports COVID exposure, or has indications of symptoms associated with COVID (coughing, fever, etc.).

Daily Health Screening

Interviewers will be required to complete a U-M ResponsiBlue Health Screening each day starting before the first Locating visit.

Interviewers will alert the BFY U-M SRC Production Management Team if the health-screen is positive for known Covid-19 symptoms and will stop working in-person until tested negative or symptom-free for at least 7-10 days (based on the current CDS guidelines).

Safety in the field
When working in the field, if the interviewer determines that there isn't a way to safely social distance they may omit to work that area or building. The interviewer must report details of the conditions to their Team Leader (TL). Safety is a priority when working on this effort and will be at the interviewer's discretion.

Interviewers must follow all State regulated CDC guidelines while working on this effort.

- They will be required to stay 6 feet apart from respondents and informants and wear a mask at all times.
    - Interviewers will **NOT** enter respondent's or informant's homes during this effort.
    - Exceptions may include apartment lobby's, foyers, hallways. However, interviewers will also individually assess whether it would be safe for interviewers to enter any of the apartments / public housing units.

- Avoid touching surfaces
- Practice hand hygiene and respiratory etiquette
- Social distance
- Wear a mask
- Proper disposal of used Person Protection Equipment (PPE).
- Stay home when appropriate

Interviewers will be expected to knock on doors to speak with the respondent if located, but also to speak with CPs, current residents, or neighbors who might be able to provide information. Interviewers will:

- only work in their assigned state / local municipality.
- Work a maximum of 2 non-consecutive days a week,
- Visit a maximum of 2 addresses on a given trip.
- Enter a call record for every household contacted in-person, whether a neighbor's house, the respondent, or another informant. Must state where the contact took place and who the contact was with.
- Limit interactions with any given household to less than 15 minutes to meet current guidelines on minimizing exposure.
- Terminate the visit if respondent/informant indicates they experience or have been exposed to one of the Covid-19 symptoms.

Contact Tracing Logs
Interviewers will be instructed to enter a log with a description of the visit to each household. The note will include address and brief information about the persons contacted.

When interacting with the recruited study participants, interviewers will collect any available contact information (confirm first / last name, phone, email address, etc.).

When interacting with an informant (e.g., another unrelated person residing at the address or a neighbor) due to privacy and confidentiality protection interviewers will record only gender of the informant (e.g., "female informant") and will not be asked to collect detailed contact information (names, phone numbers, etc.).

Leave Behind
As part of the locating visit interviewers may also leave a small gift (a gift for Mom, book, diapers, etc.) or, if no one is home, leave a small card with address information hanging on the door-knob. In some cases, interviewers may also deliver post-interview payments in cash to families who do not use banking services and may experience difficulties processing mailed payments via check.

Additional Permission for Spanish-Speaking Locating Travel
BFY currently does not have any Spanish-speaking interviewers in Louisiana and Nebraska. We would like to obtain permission for a willing Spanish-speaking interviewer to travel to Nebraska from Minnesota. This would represent a slightly higher exposure risk involving travel to/from Nebraska by car or plane and residing at the hotel for 3 - 4 days to conduct the locating trips. The trips would be scheduled at maximum once per month.

Summary of Current Covid-19 Regulations / Restrictions in BFY Data Collection States:

In summary, as of January 10, 2021, the BFY project would meet the following guidelines for safe visits as specified by local regulations:

NY: New York State has implemented a micro-cluster strategy for imposing restrictions due to Covid. Clusters are flagged as red, orange, and yellow based on the state outbreak criteria. The highest level in the metropolitan NYC clusters (Manhattan, Bronx, and Brooklyn clusters), where most of the BFY sample is clustered, is currently yellow. Under that level, the state permits non-residential indoor and outdoor gatherings of maximum 25 people. Orange clusters are also allowing non-essential businesses to operate with appropriate distancing, PPE, and capacity restrictions. The state's website features an address-level check. We plan to instruct the NY team to check addresses intended to visit to determine the level/color of restrictions before the locating trips.
Source:
https://forward.ny.gov/micro-cluster-strategy
https://forward.ny.gov/cluster-action-initiative

NE: Nebraska Department of Health and Human Services imposes tiered color restrictions by zone according to the threshold of available hospital beds. The tier/levels are green, blue, yellow, orange, and red. Under the strictest (Red) level, all indoor and outdoor gatherings are limited to 10 and 25 persons respectively and the essential business can remain open provided appropriate protection, and distancing is observed. As of 12/24 all counties in Nebraska are determined to be under the Blue level with lesser restrictions limiting indoor gatherings to 75% and lifting all restrictions on the size of outdoor gatherings while the protective guidance is still recommended (wearing masks and social distancing).
Source:
http://dhhs.ne.gov/Pages/COVID-19-Directed-Health-Measures.aspx
http://dhhs.ne.gov/Documents/DHM-Measure-Table-ENGLISH.pdf

MN: As of January 10, 2021 MN has loosened restrictions on social gatherings. Per the MN Governor Emergency Executive Order 21-01, the current limit on outdoor social gatherings has been raised to 15 people consisting of no more than 3 households. Participants of any social gathering must adhere to social distancing and safety guidelines. Businesses are allowed to operate but must adopt a Covid-19 Preparedness Plan including criteria that are met by the BFY project. The preparedness plan should require work from home whenever possible, ensure sick workers stay at home, establish social distancing policies, ensuring worker hygiene and source control, as well as cleaning, disinfection, and ventilation protocols. In addition, all customer-facing businesses

must include requirement that workers maintain physical distancing of six feet, which are also met by the BFY project.

Source:
https://mn.gov/covid19/assets/EO%2021-01%20Final_tcm1148-462106.pdf

LA: The most recent executive order recognizes that Louisiana is under a public health emergency and imposes a statewide mask mandate with which our project will comply. The current Phase 2 order is in effect until January 13, 2021. Under the Phase 2 order, all LA residents must wear a face covering unless they will not come into contact with others or will be able to maintain appropriate distancing of six feet apart. Non-essential businesses may remain open provided they observe capacity restrictions for indoor spaces and maintain social distancing. There are explicit exceptions - businesses that shall remain closed - and include places where many individuals may gather without observing social distancing guidelines such as, amusement parks, water parks, concert, music halls, etc.

Source:
https://gov.louisiana.gov/assets/ExecutiveOrders/20-1222-209-JBE-2020-AS.pdf

Zoom Health

Zoom health will only be utilized if a participant prefers use video call rather than a phone call for the interview and survey questions. It is HIPAA approved and has all security requirements that match best practices for sensitive conversations. We would follow the following configuration when PII is likely to be discussed:

- New password or meeting ID between every participant
- Lock meeting after expected participant has joined
- No social security measures

Age 3 Data Collection Protocol

Overview

In Baby's First Years, Age 3 participants recruited in the Baseline will be asked to participate in the 3rd year phone interview scheduled within two to four weeks before or after their child's third birthday. Just like Age 1 and Age 2 interviews, the Age 3 phone interview will be scheduled and conducted by a University of Michigan Survey Research Center (U-M SRC) interviewer. Consistent with the Age 2 protocol, the Age 3 interviews will be administered via phone (CATI).

Prior to the Age 3 interview, all families will be sent brief advance letters including information about the Age 3 interview, a pre-payment check of $50, and a paper copy of the Age 3 consent. Interviewers will follow up with phone calls (or emails and text messages if more appropriate) to arrange for the best time to conduct the phone interview. Similar to Age 2, Age 3 interview's length is estimated to be between 60 and 75 minutes.

At the beginning of the Age 3 interview, the interviewer will confirm that the respondent received a paper version of the consent. They will also read the Age 3 consent to the respondent, provide an opportunity to ask any questions, and obtain the oral consent which will be recorded in the instrument. If requested, interviewer will mail another paper copy of the consent.

Age 3 Visit Protocol

Eligibility

We will conduct Age 3 interviews with participants who consented to participate in the Baby's First Years study during the Baseline interview and accepted the BFY Gift during the Age 0 visit.

Participation in the Age 1 and Age 2 interviews is not a necessary condition to participate in Age 3. We will invite all participants who may or may not have participated in the Age 1 or 2 interviews.

We will NOT conduct Age 3 visits with participants whose recruited child is deceased.

If a participant asked to withdraw from the study during Age 2 they may be contacted again in Age 3 to confirm their withdrawal and to determine the reasons for withdrawal. However, they will not be contacted for the purpose of conducting the Age 3 interview unless they re-consider and decide to continue participation. Participants who withdraw from the study will continue receiving the BFY Gift.

Sample Release and Pre-visit Activities

We aim to complete Age 3 interviews within a window of 4 weeks before or after the 3rd birthday of the recruited child although some participants may end up being interviewed earlier or later than planned. The advance letter with information, prepayment, and the consent will be sent approximately 4 – 8 weeks before the focal child's 3rd birthday. Approximately, 2 – 6 weeks before the 3rd birthday, sample will be released to interviewers and they will start contacting participants via phone (or email and text messages as appropriate) to schedule appointments to complete the Age 3 interview.

4MyBabyCard Payment Extension Notifications

As participants are contacted for Age 3, they will also be notified that the conditional income enhancement in the form of monthly gifts on the 4MyBabyCard will be extended from 40 to 52 months of the focal child's age. This extension was communicated to and approved by the IRB in a separate modification. Participants will be notified via mail and/or when speaking to the interviewer prior to the Age 3 interview.

Advance mailing

The specific contents of the advance mailing will include:

a) An introductory letter thanking participants for being part of the study and reminding them they will be contacted again soon (see: BFY_Age3PrecontactLetter.docx).
c) A prepayment check of $50
d) A copy of the Age 3 Research Consent (see: BFY_Age3ResearchConsent.docx).

e) The Age 3 instrument Respondent Booklet including answer scales to the instrument question which will facilitate and speed up completing of the Age 3 instrument over the phone.
f) A separate insert announcing the extension of 4MyBabyCard payments (see: BFY_4MyBabyCardExtensionInsert.docx).

Participants who provided an email address may additionally be sent advance information via email (see: BFY Email_Protocol). Participants who provided a cell phone number may also receive a short text message sent by the interviewer once the sample is released (see: BFYText Message Protocol).

Interviewer Contact Protocol
Once sample is released, the interviewer will attempt to reach participants using contact information obtained during Baseline and Ages 1-2 data collection. Interviewers will begin by calling available phone numbers on various days and times of day such as weekday morning, afternoon, or evening, as well as weekend morning, afternoon or evening. If U-M interviewer reaches an answering machine/voicemail response, they will leave a scripted message, which does not reveal the study name (see: BFY_VoicemailScript). Interviewer will leave their name and a phone number and ask for a call back to either the interviewer's work number or the U-M toll–free number.

In-person work has been suspended between March 2020 and April 2021 due to Covid-19. In-person work has been re-authorized and commenced in May 2021 for the purpose of locating participants who cannot be reached via phone or other means or to deliver Age 3 tokens of appreciation to participants who do not receive mail or are unable to cash the checks. If participants cannot be reached using the available phone numbers, interviewers may attempt to go in person to the address provided during recruitment. Interviewers may leave "Sorry I Missed You" (SIMY) cards with the interviewer's work number or the U-M SRC's toll free number (see: BFY-SIMY_Card.doc). Interviewer may also refer hard-to-reach participants to a specialized U-M SRC locating team. The locating team calls available numbers and uses directory assistance to obtain additional information. The locating team may also conduct internet searches to obtain updated address or telephone numbers. Additionally, at the time of recruitment, some participants provided alternative contact information including phone, email, or address of the best person to contact if the participants are hard to find. Interviewers and/or the locating team may reach out to designated contact person(s) asking for help in reaching the participant. They may also ask contact person(s) to let the respondent know we are trying to reach them and ask them to contact the U-M SRC toll-free number. In some cases, we may offer a small finder's fee of $10 to $20 in appreciation for assistance in locating a BFY participant.

Depending on available contact information, interviewers may also try to reach participants via email or text messages. Email and text messages may also be used to remind respondents about upcoming appointments. (see: BFY_Email_Protocol.doc and BFY_Text Message Protocol.doc)

Upon reaching participants, interviewers will confirm whether a participant received the Age 3 advance letter, respond to any questions, and setup an appointment to conduct the Age 3 phone interview.

Unexpected situations
While scheduling the Age 3 interview, interviewer may come across some unexpected situations.

State Residence and Eligibility for Social Services

Prior to scheduling a visit, interviewers will confirm a participant's current address. If Mom moved out of the recruitment state she will be informed that she can continue to participate in the study and continue receiving the BFY Gift. However, she will also be informed that the BFY Gift may no longer be excluded from eligibility determination for receiving social services from the new state of residence. Interviewer will record whether or not Mom decided to continue or to stop receiving the gift and whether or not she is interested in further participation in the study. Interviewers will refer participants to obtain further information and support from Lauren Mayer, Baby's First Years Project Director at Teachers College Columbia University, at (608) 291-7359 or via email at 4mybabycard@gmail.com.

Incarcerated or Institutionalized Participants

Participants who are currently incarcerated or institutionalized will not be asked to participate in the Age 3 interview. Interviewers will attempt to determine how long the incarceration or institutionalization may last to determine whether Mom's release date would allow us to interview her during Age 3 data collection or whether she should be re-contacted in the future waves.

Child Status

Mothers who are either permanently separated from the focal child or have not seen the focal child for more than 6 months (through foster care, adoption, or other reasons) will not be asked to participate in the Age 3 interview.

Deceased Participants

Mothers of children deceased before Age 3 will not be asked to participate in the Age 3 interview. Interviewer may attempt to determine when the death occurred. Similarly, families of mothers who are deceased will not be contacted and their BFY Gift will cease to be issued to the participant's debit card.
Age 3 interview:
Participants can choose the most convenient time to complete their Age 3 interview. U-M SRC interviewers will be prepared to conduct the Age 3 interviews via phone and in a few limited cases of hearing impaired participants, the interview may be conducted via Zoom Health. Approximately 24 hours before the interview, interviewers will remind participant about the upcoming appointment via a phone call, email, or a text message (see: BFY_Text Message Protocol.doc) depending on participant's preference.

Age 3 Intro and Consent

Interviewers will begin the interview by confirming that the participant received the advance letter including the prepayment check and the paper copy of the Age 3 consent.

Interviewer will also confirm that the respondent received and understood the information about the extension of the 4MyBabyCard information and were able to cash the prepayment check.

Interviewers will begin the interview by confirming that the child is well, with Mom and that the respondent is ready to begin the interview (Section A of the BFY Age 1 Instrument).

Interviewers will then proceed to explain the Age 3 Research Consent in lay language and allow participants to ask questions before providing oral consent. It will be made clear that if a participant no longer wishes to partake in the study, she can withdraw participation at any time with no consequences. Interviewers will also offer to re-mail the paper consent. Since Age 3 interviews are conducted via phone, similar to the protocol in Age 2, the interviewer will obtain oral consent, date and sign electronic consent as a witnesses and write-in that consent was conducted via phone in lieu of respondent signature. A paper copy of the Age 3 Research Consent will be provided to each participant in the advance mailing.

Instrument A and Instrument B

Similar to Age 2, Age 3 instrument includes two versions, A and B. Version A includes maternal questions as well as questions about the focal child. Version B excludes child-related content. In rare situations, it may become obvious that mother has been separated from the child either on a temporary basis or permanently. If the focal child lives with adoptive parents, the interview will not be conducted at all. However, if child lives with a foster parent and/or mother has spent at least some time with the child in the past 6 months, participants will be asked an abridged version B of the instrument which includes only questions intended for the mother and excludes all child-related content.

Voluntary Statement and Consent to Record

Following standard U-M SRC procedures, the interviews will be recorded for quality control purposes only. Recordings are encrypted and are transmitted to SRC offices via secure FTP. Recordings are not included in the list of project deliverables sent to the University of Wisconsin. They will be securely destroyed by SRC at the end of the study. The following statement will be read to all respondents before the interview: "Before we begin, I would like you to know that this interview is completely voluntary and the information you provide is confidential. If we should come to any question you do not want to answer, please tell me and we'll go on to the next question." Respondents are also read a statement about recording and have the option to opt-out "Parts of this interview may be recorded for quality control purposes only. If you do not wish to be recorded, please let me know. You can still participate in the interview." If the respondent does not want to be recorded, the interviewer turns off the recording.

Age 3 Main Instrument (CATI)

Age 3 instrument will be administered via phone (CATI). Interviewers start Age 3 interview by administering a Module: “Intro” including Section A (Intro & Consent) and C (Household Roster). Interviewers then proceed to complete the remaining modules: Mom 1, Mom 2, ACASI, and Closing.

The Age 3 ACASI module includes some potentially sensitive questions. This section will continue to be administered via phone in Age 3. At the start of the section, respondents are reminded that

some questions are sensitive and interviewer confirms they can be answered privately. Respondents are reminded that they can skip any questions they do not feel comfortable answering.

Closing Module

Closing module will include
questions to obtain the current residence address at the time of Age 3 interview.
Interviewer will also indicate whether a participant revealed or the interviewer otherwise found out about the amount of cash gift Mom is receiving from the study.
Monitoring cooperation and increasing response rates:
Monitoring production will be important to ensure visits are completed within the desired window before/after the focal child's birthday. We anticipate that some participants may take longer to respond to our interview request and may voice concerns with participation. We plan to implement a few strategies to ensure participation within the specified time. Those strategies may include:

- Understanding and addressing participants concerns;
- Offering to break up one longer visit into shorter visits;
- Offering more flexible choices of dates and time for conducting the visit;
- Monitoring production and implementing initiatives to boost on-time participation
    - U-M will monitor the number of successful / unsuccessful attempts to gain cooperation and record specific concerns raised by participants.
    - Non-respondents may receive additional mailings, emails, or text messages addressing their concern and offering appropriate alternatives.
    - In some cases we may increase the token of appreciation for participating in BFY from $100 up to $200.

Reports of Child Abuse/Endangerment

Similar to Age 2, in Age 3 BFY plans to report evidence of suspected child abuse, neglect or endangerment, corresponding to statutes in the study sites included in the research, if observed by an interviewer. Although interviewers will not ask any interview questions or make any standardized observations which meet the definition of criminal child abuse, neglect or endangerment, interviewers may very rarely be exposed to a situation or a participant feedback which requires a mandated report. Interviewers are instructed to file a report with a licensed clinician at the University of Michigan SRC Clinical Contact Protocol team (CCP). The CCP team triages the case and determines the appropriate next steps including working the interviewer to complete mandated reporting as required by local state guidelines.
Counseling or Support for Study Participants
In addition to handling mandated reports, the SRC has a referral system in place whereby any respondent requesting or, in the judgment of the non-clinician interviewer, requiring further assistance is referred by interviewers to a licensed social worker with mental health experience. The Baby's First Years respondents and interviewers will be supported by the CCP team which consists of licensed social workers with mental health experience, who are available to support the Survey Research Center's interviewing efforts.

The CCP members take referrals from interviewers. Upon receipt of an email or phone request, the CCP will follow up directly with respondents (and interviewers) and will provide confidential referrals for local support services. Typically, the support to respondents includes referrals to clinical, mental health, and other related services in the local area, though sometimes the CCP member simply talks directly with the caller and no further services are necessary. The social worker will contact the individual by telephone, assess the needs of the respondent(s), and connect the respondent with local services. Interviewers engage the CCP through a request in a secure web portal.

Interviewers are not tasked or required to provide counseling assistance to respondents, other than to take information for a referral to a CCP, or refer to numbers from the Hotline List when requested to do so by the respondent. The CCP will assess the respondent's situation and make appropriate referrals.

Age 4 Data Collection Protocol

Overview

In Baby's First Years, Age 4, participants recruited at Baseline will be asked to participate in the 4th year university lab visit ("the Visit") scheduled within four weeks before or after their child's fourth birthday. Unlike the Age 1, 2, and 3 phone calls, for which the University of Michigan Survey Research Center (UM SRC) conducted data collection, Age 4 lab visits will take place in local university labs by locally hired research staff in the four study sites. Reliance agreements for the three sites outside of New York City have been established under this protocol. Our local university partners are:

- Minnesota: University of Minnesota
- Nebraska: University of Nebraska at Lincoln
- Louisiana: University of New Orleans
- New York: Teachers College, Columbia University

Prior to the Visit, all families will also receive a call from a UM SRC interviewer to notify them that outreach will be happening from the local university moving forward. Study staff in the university labs will follow up with phone calls, emails, and/or text messages) to arrange for the best time to schedule the Visit. The Visit is estimated to last between 2.5 and 3 hours. At the beginning of the Visit, the interviewer will seek informed consent, give participants an opportunity to ask any questions, and ask for a signature before data collection begins.

Participants will receive up to $310 in cash in appreciation for their time, plus transportation support, a meal, and a small gift for the child.

Eligibility

We will conduct Age 4 Visits with participants who consented to participate in the Baby's First Years study during the Baseline interview and accepted the BFY Gift during the baseline hospital visit.

Participation in the Ages 1, 2, and/or 3 interviews is not a necessary condition to participate in Age 4. We will invite all participants who may or may not have participated in prior data collection waves.

We will NOT conduct Age 4 visits with participants whose recruited child is deceased.

If a participant asked to withdraw from the study during prior study waves, they may be contacted again to confirm their withdrawal and to determine the reasons for withdrawal. However, they will not be contacted for the purpose of conducting the Age 4 Visit unless they re-consider and decide to continue participation. Participants who withdraw from the study will continue receiving the BFY Gift through 76 months of age

For future waves of data collection beyond the scope of the initial study, the study team will contact all eligible participants to inform them of data collection activities. Participants will be told that they have the right to decline to participate at any time.

Pre-visit Activities, Sample Release, and Scheduling the visit

We aim to complete Age 4 Visits within four weeks before or after the fourth birthday of the recruited child, although some participants may end up being interviewed earlier or later than planned. Because data collection activities and participant communication will be transferred from the UM SRC to the local research labs, careful consideration has been made to communicate this to participants in an organized way.

Approximately 2 months prior to the child's birthday, UM SRC will contact participants and share information about the upcoming university visit and contact information for the local university lab with participants (see: SRC interviewer 46 month script). 3-6 weeks after that, participants will receive communication from the local lab via calls, emails, and/or texts, and in some cases face-to-face visits to schedule the upcoming visit.

At this time, should the participant choose to participate in the age 4 study activity, information on language preference of the participant, language(s) the child speaks, diagnosis of child health problem or disability, visit transportation, additional family members that the participant may bring to the visit, and snack preferences will be collected from participants to prepare for the visit. Participant questions will be answered, and the visit will be scheduled.

Participants who provided an email address may additionally be sent advance information via email (see: BFY Age 4 email scripts). Participants who provided a cell phone number may also receive advance information via a short text message sent by the research assistant (see: BFY Age 4 text message scripts).

For some research participants, WhatsApp is a more reliable platform for day to day communication than a traditional phone/text plan. To accommodate, in addition to phone calls and SMS/texts, the research team will communicate with participants about the visit and study activities via WhatsApp, using the text message scripts. In line with TCIT Information Security's guidance, no protected health information (PHI), personally identifiable information (PII) or sensitive participant data will be collected using the application.

After scheduling the visit, research assistants will send an informational email and text reviewing the details of the visit. Then, a few days before the scheduled visit date, research assistants will send a confirmation email and text to participants confirming details regarding the visit (BFY Age 4 email scripts).

Research Assistant Contact Protocol

Once participants age into the contact window, the interviewer will attempt to reach participants using contact information obtained during prior data collection waves. Research assistants will begin by calling and texting available phone numbers on various days and times of day such as weekday morning, afternoon, or evening, as well as weekend morning, afternoon or evening. If the research assistant reaches an answering machine/voicemail response, they will leave a scripted message. (see: BFY Age 4 voicemail scripts). Study staff will include the study name ("Baby's First Years") in messages as an important identifier of the activity, but will not include the words "study", "participant", or "participation", for participant privacy. The research assistant will leave their name and a phone number and ask for a call back to either the research assistant's work number or the lab's phone number.

If participants cannot be reached using the available phone numbers, research assistants may attempt to go in person to the address provided during recruitment. Research assistants may leave "Sorry I Missed You" (SIMY) cards with the research assistant's work number or the lab's number (see: BFY Age 4_SIMY_Card.doc). Research assistants may use directory assistance to obtain additional information, and conduct internet searches to obtain updated address or telephone numbers. Additionally, during prior waves of the study, some participants provided alternative contact information including phone, email, or address of the best person to contact if the participants are hard to find. Research assistants may reach out to designated contact person(s) asking for help in reaching the participant. They may also ask contact person(s) to let the respondent know we are trying to reach them and ask them to contact the lab's phone number. In some cases, we may offer a small finder's fee of $10 to $20 in appreciation for assistance in locating a BFY participant.

Depending on available contact information, research assistants may also try to reach participants via email, text, or WhatsApp messages. Email, text, and WhatsApp messages may also be used to remind respondents about upcoming appointments. (see: BFY Age 4 email scripts and BFY Age 4 text message scripts)

TEACHERS COLLEGE
COLUMBIA UNIVERSITY
Institutional Review Board
Box 151 | 525 West 120th St. New York, NY 10027
212-678-4105 | RH 13 | IRB@tc.edu | tc.edu/IRB

Unexpected situations

During pre-visit contact, research assistants may come across information that will require a specific course of action or to provide the participant with more information about her participation in the BFY study. Participants who cannot take part in the Age 4 visit will continue to receive the BFY Gift unless they specifically opt-out and choose not to receive cash payments.

State Residence and Eligibility for Social Services

Prior to scheduling a visit, research assistants will confirm a participant's current address. If Participant moved out of the recruitment state she will be informed that she can continue to participate in the study and continue receiving the BFY Gift. However, she will also be informed that the BFY Gift may no longer be excluded from eligibility determination for receiving social services from the new state of residence. The research assistant will record whether or not the Participant decided to continue or to stop receiving the gift and whether or not she is interested in further participation in the study. Interviewers will refer participants to obtain further information and support to the 4MyBaby card hotline at (608) 291-7359 or via email at 4mybabycard@gmail.com.

Consenting for Future Waves of Data Collection

At baseline, most participants gave consent to be contacted for future research beyond the initial scope of the study. During the age 4 visit, participants who did not provide that consent at baseline will be asked again and have the opportunity to consent to be contacted for future waves of the research.

Incarcerated or Institutionalized Participants

Participants who are currently incarcerated or institutionalized will not be asked to participate in the Age 4 Visit. Research assistants will attempt to determine how long the incarceration or institutionalization may last to determine whether Mother's release date would allow us to interview her during Age 4 data collection.

Child Status

Mothers who are permanently separated from the recruited child (through non-familial foster care, adoption, or other reasons) will not be asked to participate in age 4 data collection. However, their BFY Gift will continue to be loaded in alignment with the rest of the sample .

Deceased Participants

If the recruited child passed away, participants will not be asked to participate any further. Interviewer may attempt to determine when death occurred. Families of mothers who are deceased will not be contacted. Their BFY Gift will continue to be loaded through 52 months but the participant will not be eligible for any future BFY Gift extensions.

4MyBabyCard Payment Extension

As participants are contacted for Age 4, they will also be notified that the conditional income enhancement in the form of monthly gifts on the 4MyBabyCard will be extended from 52 to 76 months of the focal child's age. Participants will also be notified via Study Newsletter.In alignment with the prior cash gift extension, gift payments will be extended to all participants on an opt-out basis, meaning that all will receive the additional payments unless they indicate that they do not want the extension. We propose to extend to everyone except: those who indicate that they do not want the payments; and those who are deceased.

Age 4 Visit Activities

Participants can choose the day and time of Age 4 visits. Research staff at the four university locations will be prepared to conduct the Age 4 visits in their respective labs. For cases in which it is not feasible for participants to come to the university labs for data collection, alternative arrangements will be made for phone-based data collection, or data collection in another location preferred by the respondent.

Primary Research Staff and Assistants

For Age 4 lab visits, a staff of two primary researchers (Researcher 1 and Researcher 2) will be present to conduct the maternal survey, child cognitive assessments, EEG, and biomarker collection components of the visit. Additional assistants will also be present to provide sibling childcare, help with equipment set up and take-down, or provide assistance with maintaining the flow of the visit. Assistants who are appropriately trained may perform data collection functions.

Primary research staff and assistants are hired by local site PIs and managed by a Site Coordinator at each site. The Site Coordinator arranges for staff and assistants to be available to conduct and help with lab visits, phone interviews, or alternative location visits, when possible. There is one coordinator at each of the four study sites. The coordinator is the liaison between researchers and assistants and manages the research staff and assistants' schedules, accommodating in-person visit schedule changes and communicating cancellations.

Age 4 Intro and Consent by Data Collection Mode

While the majority of age 4 data collection will take place in the university labs, in some limited circumstances data collection will take place via phone, to accommodate participant preferences. Details for phone interview eligibility can be found under "Age 4: Alternate Mode Protocol".

In-person visit

On the day of the visit, after leading the participant and child to the lab, Researcher 1 will proceed to explain components of the Age 4 visit as described in the Research Consent. All Age 4 procedures will be explained in lay language, and participants will have the opportunity to receive answers to their questions before signing. Researcher 1 will offer to read the full consent form, if needed. Participants will have the opportunity to read it themselves. It will be made clear that if a participant no longer wishes to partake in the study, they can withdraw participation at any time with no consequences. If the visit is conducted in person, interviewers will obtain respondent signature on a digital Age 4

Research Consent (see: BFY_Age 4 Consent). A paper copy of the Age 4 Research Consent will be provided to each participant upon request.

Phone interview

In certain instances where a mother and child cannot come into the lab for the visit, or would prefer to take the survey in advance of an in-person visit, the researchers may offer a phone interview to procure Age 4 data. In these cases, the only component of the protocol that will be administered is the Maternal Survey. Researchers will email or text a copy of the consent form, and will also offer to read it to the participant. All Age 4 maternal survey procedures will be explained in lay language, and participants will have the opportunity to have any questions answered before verbally providing consent, which will be recorded and initialed by the researcher. Before reading the parts of the consent form that describe in-person activities, the researcher will remind the participant that they may choose to skip any research activity or question at the time of the call and the lab visit, and that signing the consent form does not obligate them to any research activity.

Researchers will begin the interview by confirming some of the data obtained during previous waves of data collection (child's date of birth, etc.).

It will be made clear that if a participant no longer wishes to partake in the study, they can withdraw participation at any time with no consequences. The researcher will date and sign electronic consent as witnesses and write-in that consent was conducted via phone in lieu of respondent signature. After the phone survey, payment in the form of an electronic gift card will then either be emailed, texted, or mailed to the participant according to their preference.

Split-mode visit assisted by an alternate adult

In some instances, the participating mother may not be able to visit the lab herself, but consents to one or more of the child measures. In these cases, researchers will begin the interview by phone, confirming some of the data obtained during previous waves of data collection (child's date of birth, etc.), and collect verbal consent. Researchers will email or text a copy of the consent form, and will read the full text to the participant if desired. All Age 4 procedures will be explained in lay language, and participants will have the opportunity to have any questions answered before verbally providing consent, which will be recorded and initialed by the researcher. For participants who provide consent to the child activities and would like to send the child to the lab with another adult, the researchers will collect contact information for the chaperoning adult and will schedule the visit either with the participating mother or directly with the chaperoning adult, at the preference of the participating mother. Compensation will be provided directly to the mother in the form of an electronic gift card that will be emailed, texted, or mailed, according to the mother's preference.

Transportation compensation

The study team will arrange round-trip car services for participant transportation to the nearest research site.

If participants decline a lab-provided car service and choose to travel on their own, they will be reimbursed upon arrival. Payment for travel is dependent upon mileage distance (round trip) from the participant's home to the study site as follows: $25 for 1-40 miles round trip, $50 for 41-80 miles round trip, $75 for 81-120 miles round trip, $100 for 121-160 miles round trip. If the start and end points are different, participants will be paid for the total miles driven. The rates are above the current IRS mileage reimbursement rate of 58.5 cents per mile, and will be adjusted accordingly if there is a change throughout the data collection wave.

Individual arrangements will be made with participants who live > 3 hours' drive from a research site. All participants in this category will be offered the following: Mileage reimbursement; 2 nights at a local hotel (1 room); 5-6 meals per person; and reimbursement for a local activity for children. Reimbursement will be adjusted for the number of family members visiting, which will be capped at four people.

Voluntary Statement and Consent to Record

Some of the data collection will be recorded for quality control purposes only. Recordings are initially saved to a password protected SD card and then uploaded onto TC's Google Drive. Once, transferred to TC's Google Drive, they are deleted from the SD card.

Participants will have the opportunity to opt out of this recording.

Age 4 Visit, Data Collection Activities

For in-person visits, researchers will administer the following Age 4 visit activities. They will be administered in the proposed order in most cases, but the sequence may change depending on the needs of the individual participant.

Data security, transfer, and storage note: unless otherwise noted below, data will be stored on TC's servers, which are secure and password protected, and then sent to the University of Wisconsin via a Secure FTP connection and stored in the BFY Data Repository maintained by the University of Wisconsin.

Age 4 Activities: Parent-Child Interaction

The objective of parent-child interaction is to record 10 minutes of play-based interaction.

The researcher will introduce this component and tell the participant that the interaction will be videotaped using a video camera. If the participant does not want to be videotaped or refuses for another reason, this component will not be conducted. If timing is not appropriate and participant would like to conduct this part of the visit later, then the researcher will accommodate.

The researcher will lead the mother and child to a room where they will be by themselves during this free play activity. As the researcher leaves the room, they will ask the mother and child to remove their masks for these 10 minutes to ensure high-quality data is recoded by the video

camera. After this activity is complete, the mother will be asked to put the mask back on herself and her child.

Researchers will provide a picture-book and a toy food playset to facilitate the parent-child interaction. Toys will be sterilized after each use. Participants will be asked to play with their child for a total of 10 minutes. First they will interact with the picture-book for about 5 minutes. For the remaining 5 minutes, a researcher will provide the Mother and child with the toy food playset. During the 10 minutes of playtime, they will be videotaped.

Data security, transfer, and storage note: parent-child interaction recordings are initially saved to a password protected SD card and then uploaded onto the secure TC server, which is password protected. Once, transferred to TC's server, they are deleted from the SD card. Data is then sent to the University of Wisconsin via a Secure FTP connection and stored in the BFY Data Repository maintained by the University of Wisconsin.

Age 4 Activities: LENA Recording

During parent-child interaction, the child will be asked to wear a small, child-safe recorder worn in a comfortable vest. The recorder "meets U.S. and international safety standards for electronics and toys. Unlike a cell phone, it does not transmit, and it uses the same type of low-power processors as hearing aids." (source: https://www.lena.org/technology/). Approximately 10 minutes of voice recording is translated and processed using automatic Language Environment Analysis (LENA) algorithms "in the cloud" to produce the word counts, turn counts, and other information about the recording.

Data security, transfer, and storage note: LENA recordings are identified by participant ID and no personally identifiable information is shared with LENA. Once recording is completed offline, interviewer uses LENA processing software to extract the files and process locally. The recording is sent "to the cloud" to apply a scoring algorithm and return the file to the local interviewer laptop. After processing, the audio is automatically deleted from LENA server as soon as analysis completes. LENA does not listen to or keep participants' recordings. Once recordings are processed and stored on interviewer laptop they are offloaded via a secure VPN and deposited on TC servers. Data is then sent to the University of Wisconsin via a Secure FTP connection and stored in the BFY Data Repository maintained by the University of Wisconsin.

Age 4 Activities: Age 4 Maternal Survey

This survey will gather data from participants that include the following elements: household roster, residential information, labor market participation, income, expenditures on the child, childcare, parent-child activities, child behaviors and developmental status, healthcare, economic stress, food insecurity, parenting stress, maternal mental health, relationship quality, and life events (see: BFY Age 4 survey instrument)

Age 4 Activities: Child Cognitive Battery

To complete the cognitive battery, the child will sit with Researcher 1 at a child-friendly table in a room that minimizes distraction. Though Mother will be separated from the child at the point of the visit (unless requested otherwise), a baby monitor camera is placed in the room so that Mother can observe from the room where she completes her own cognitive testing and the Maternal Survey

(see: BFY Age 4 survey instrument). Both Mother and child are informed that they are free to take a break or see each other at any point during the visit. The child is provided with a sticker chart and a sheet of stickers so that progress can be tracked throughout the session and motivation is maintained. The child is also provided with optional snacks and water during the cognitive battery testing.

Minnesota Executive Function Scale (MEFS)
Children will complete a brief measure of executive function by completing a brief sorting game on a laboratory iPad. A researcher will guide the participant through the game by reading the on-screen instructions and providing feedback where necessary. The task's difficulty is adjusted to the child's age, and accurate performance leads to more challenging trials. Inaccurate performance will lead to less challenging trials, until the measure is complete. This task usually takes 5 – 7 minutes for the child to complete.

Receptive One-Word Picture Vocabulary Test-4 (ROWPVT-4; Monolingual and Bilingual)
Children's vocabulary will be measured using the ROWPVT-4. During this assessment, a researcher presents a flipbook that displays 4 pictures on each page. The researcher states one word (e.g., "doll") and the participant is instructed to point to the picture that matches or to say the number above the picture that matches. When the participant gets a pre-determined number of items incorrect, the testing concludes. This assessment typically takes 10-15 minutes. This test can also be administered with instructions for Bilingual (English-Spanish) participants.

Weschler Nonverbal Scale of Ability Subtests (WNV)
The Matrices subtest of the WNV will be collected. During this assessment, a researcher presents a stimulus book laid flat in front of the child on the table. The Matrices subtest examines children's reasoning skills by asking them to choose an image from a series of distractors that best matches a group of images at the top of the page ("Which one of these goes here?" as the researcher points). The task is similar to choosing which puzzle piece fits best according to a pattern. After the first few practice trials, the assessment is administered without verbal instruction or direct feedback to maintain the nonverbal nature of the assessment. The assessment continues until a pre-determined number of incorrect trials is reached. This subtest typically takes 5-10 minutes depending on the child's mastery of the task.

The Reading House (English and Spanish)
The Reading House is a measure of children's pre-literacy skills. Children are handed a small book and asked a series of questions about the physical book itself, the letters and sounds on the inside of the book, the names of various items displayed, etc. Children are also asked to write their name on the back of the book, if able. This assessment typically takes 5-10 minutes to complete. There is both an English and Spanish version, depending on the child's preferred language of communication. At the end of the session, children are given the book as part of a take-home gift.

Pencil Tap

Pencil Tap is a measure of children's inhibitory control. In this assessment, children are provided with one large, unsharpened pencil and the researcher holds an identical pencil. Children are instructed that they are to listen to the researcher tap the pencil on the table in front of them. The researcher says, "When I tap my pencil one time, you tap your pencil two times. When I tap my pencil two times, you tap your pencil one time." The first few trials are used as practice and the child is given feedback, where necessary. After practice, children complete a total of 16 trials. This assessment typically takes about 3 – 5 minutes.

Age 4 Activities: Minnesota Executive Function Scale (MEFS)

The mother will also complete the MEFS. Though starting on a higher level of difficulty, administration of the MEFS is similar to that of children, with the researcher reading instructions allowed and helping to provide feedback where necessary. This assessment usually takes 5-7 minutes for the participant.

***Data security, transfer, and storage note*: MEFS data, participant DOB, and gender are identified by participant ID and uploaded to the Reflection Sciences, the purveyor of the instrument, in a cloud-based portal. Once a score is produced, identifiable information is downloaded and removed from their portal and offloaded via a secure VPN and deposited on TC servers. Data is then sent to the University of Wisconsin via a Secure FTP connection and stored in the BFY Data Repository maintained by the University of Wisconsin. Some de-identified data will remain in the portal for Reflection Sciences own uses, including further improving the service. Reflection Sciences will safeguard the de-identified information according to commercially reasonable administrative, physical and technical standards (e.g., National Institute of Standards and Technology, Center for Internet Security, Gramm-Leach Bliley Act, Payment Card Industry Data Security Standards (PCI-DSS)) and shall not disclose the same except as required by the established agreement or authorized by Teachers College in writing. The License Agreement with Reflection Sciences was reviewed and approved by the TC general counsel to ensure adequate protection of participant privacy and data security. It was agreed that the deidentified information provided to Reflection Sciences was outside of the framework of PII.** **Age 4 Activities: Epigenetics / Saliva Collection**

During Age 4 visit, researchers will collect saliva samples from both mother and child. Saliva will be collected using kits provided by DNAGenotek (DNA Genotek Inc. www.dnagenotek.com).

Researchers will begin by showing the participant a brief overview video, and answering any questions (see: BFY_video_epigenetics intro).

At the beginning of the visits, researchers will ask participants not to eat or drink anything including water for approximately 30 minutes before collecting saliva samples. Prior to the collection, interviewers will confirm if participants ate, drank anything including water, smoked, or chewed gum within 30 minutes of donating saliva.

After collection, saliva tubes will stored at the local university labs, and batch mailings will be sent to a lab at the Max Planck Institute for chemical analysis. Samples will be only identified by participants BFY Sample ID and no personally identifiable information will be included on the shipment.

Samples will be analyzed for epigenetic aging and methylation. Data analysis will be limited to the epigenome, not the genome.

Saliva collection protocol:

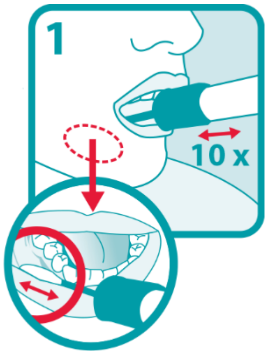

Step 1
Open DNAGenotek package with the "OPEN HERE" arrow
Remove collector by its handle from the packaging (ensure sponge tip does not come into contact with any surface prior to collection!)
Place sponge as far back in the mouth as comfortable and rub along the lower gums in a back and forth motion
Gently rub gums 10 times (if possible avoid rubbing teeth)
Tips for 10 mos and up: Tell them a little tickle will happen and try to give them a job of some sort (like holding something you'll need later or pointing to different stickers you say in the room)

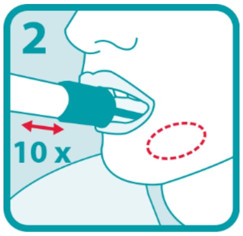

Step 2
Gently repeat rubbing motion on the opposite side of the mouth along the lower gums for an additional 10 times

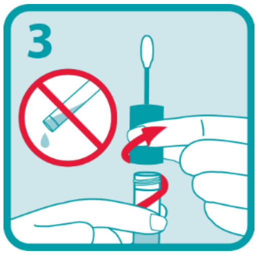

Step 3
Hold the tube upright to prevent the blue liquid inside the tube from spilling
Unscrew the blue cap from the collection tube without touching the **sponge**

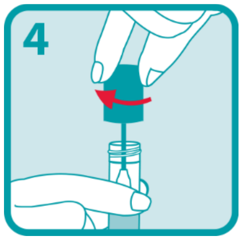

Step 4
Turn the cap upside down
Insert the sponge into the tube and close cap tightly

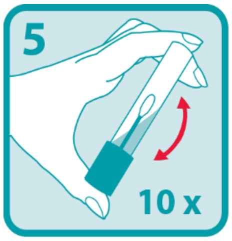

Step 5
Invert the capped tube and shake vigorously 10 times

Step 6
Put sample in biohazard bag and seal bag closed bag by peeling off blue liner
Sample will be labeled with the following information:
Study Name: BFY
Participant ID
Parent/Child (circle one)
Collection date
Time collected and AM/PM (circle one)
Interviewer initials
Sample will be stored in the local labs and then shipped in batches to the Max Planck Institute for chemical analysis.

Saliva data security, transfer, and storage note: Saliva samples will be mailed without any identifying information to a lab at the Max Planck Institute for analysis. Biospecimens that are collected as part of this study will not be used for commercial profit, and analysis will not include whole genome sequencing. They will be destroyed once they are processed for this study, and will not be used in future research studies. Deidentified data values will be sent from the outside labs to TC, for ultimate upload to the secure servers via FTP.

Age 4 Activities: Age 4 Height and Weight
Height and weight of both mother and child will be measured using a mechanical beam scale. Both the mother and child step onto the scale and a researcher obtains both height (cm) and weight (lbs). This typically takes 2 – 3 minutes to complete.

Age 4 Activities: Electroencephalogram (EEG)
The objective of Age 4 EEG is to measure child brain activity at age 4. Conducting EEG at this point will help to understand the effect of four years of monthly unconditional cash support on children's brain function. The researcher will begin by explaining the process to the mother by showing her a brief video and answering any questions (see: BFY Age 4 EEG Video).

BFY Age 4 EEG will be conducted using a Magstim/EGI high-density EEG system located at each site. The stretchy, child-friendly cap is outfitted with 128 leads that are attached to a stretchy cap that fits on the child's head.

The researchers will launch the NetStation software and prepare the child for the EEG. During this time, the researcher will use a tablet with an engaging video to keep the child entertained and distracted (e.g., *Finding Nemo* or *Moana*). The researcher will put a clean cap on the child's head. The cap will go over the head, and is secured with the chin strap.

Researchers will verify that each electrode is settled and detecting brain function, and after that will record the EEG data with the Magstim/EGI high-density EEG system system for ~15 minutes. During this time, children will sit quietly while watching video clips, looking at bubbles, and/or listening to sounds from a speaker (e.g., beeps, tones, animal sounds).

Data security, transfer, and storage note: Deidentified EEG data is recorded onto on a password-protected computer. Collected data is then offloaded via a flash drive and transferred to a laboratory computer for secure VPN and deposited on the TC secure drive for processing. Following processing, data is then sent to the University of Wisconsin via a secure FTP connection and stored in the BFY Data Repository maintained by the University of Wisconsin.

Age 4 Administrative Consent Instrument, Nebraska only

During the recruitment wave and again during Age 1 visit, participants were asked to provide consent to access to administrative data on social services obtained.

During Age 4 visit, participants in the Nebraska site only will be told again about the research objectives associated with their administrative data and its importance, and asked if they would consider providing consent to obtain Administrative Data.

Nebraska's Department of Health and Human Services (DHHS) requires that their Authorization for Disclosure of Protected Health Information form be completed in order to release administrative records of participants. Accordingly, Nebraska participants will be asked for consent using this form (see: Authorization for Disclosure of Protected Health Information).

PI Katherine Magnuson, Professor of Social Work at the University of Wisconsin-Madison, is listed as the recipient of the data authorized by participants. Researchers will provide the following information if asked exactly where the data is going and who will be receiving and using the data:

> "Dr. Magnuson is one of the BFY researchers, and is a professor of social work at the University of Wisconsin. We'd like to know about the social services and benefits that your family may be receiving, to understand more about your day-to-day life. Dr. Magnuson is in charge of getting this information from Nebraska's Department of Health and Human Services, and keeping it safe and confidential. DHHS will not know that you're getting the gift money, unless you already provided consent for that to happen."

Data security, transfer, and storage note: The completed form will be scanned and uploaded onto TC's secure server. Digital (scanned) copies will be deleted from the local computer and scanner after upload. The physical form will be kept in a locked cabinet in a locked room. Once data collection is complete for all participants, the physical forms will be mailed through secure shipping to Dr. Katherine Magnuson at University of Wisconsin-Madison, School of Social Work. They will be scanned and deposited on University of Wisconsin servers, and stored in the BFY Data Repository maintained by the University of Wisconsin.

Monitoring cooperation and increasing response rates

Production monitoring will be important to ensure visits are completed within the desired window before/after the fourth birthday. We anticipate that some participants may take longer to respond to our interview request and may voice concerns with participation. We plan to implement a few strategies to ensure participation within the specified time. Those strategies may include:

- Understanding and addressing participants concerns;
- Offering to break up one longer visit into shorter visits;
- Offering flexible choices of dates and time for conducting the visit;
- Utilizing assistants to reduce respondent burden by
    - Accelerating administration of the protocol (assistants will help interviewer move through the protocol quickly and reduce total amount of time spent in the lab)
    - Helping to manage distractions (e.g., assistants will help keep siblings entertained during the protocol administration);
- Monitoring production and implementing initiatives to boost on-time participation
    - Each site, monitored by a national sample management system, will monitor the number of successful / unsuccessful attempts to gain cooperation and record specific concerns raised by participants.
    - Non-respondents may receive additional mailings, emails, or text messages addressing their concern and offering appropriate alternatives.
    - In some cases we may increase the token of appreciation for participating in BFY from $200 up to $300.

Special Situations: Reports of Child Abuse/Endangerment and Support for Participants

Mandated reporting

As during previous waves of data collection, the research team plans to report evidence of suspected child abuse, neglect or endangerment, corresponding to statutes in the study sites included in the research, if observed by a staff member. Although researchers will not ask any interview questions or make any standardized observations which meet the definition of criminal child abuse, neglect or endangerment, researchers may very rarely overhear or observe something which requires a mandated report. Researchers who are mandated reporters in the state in which the interview visit is conducted will report to a study PI, who will file reports directly with the state.

Support for Study Participants

If a participant requires further assistance, the research assistant will contact the study PI who will then advise the staff to share resources directly with the participant, such as:

- Substance Abuse and Mental Health Services Administration (SAMHSA) National Helpline
- National Domestic Violence Hotline
- National Suicide Prevention Lifeline
- National Child Abuse Hotline
- Local Covid-19 support and information lines

Research assistants are not tasked with or required to provide counseling assistance to respondents, other than to take information for a referral to a supervisor, or refer to numbers from the hotline list above when requested to do so by the respondent. The supervisor will assess the respondent's situation in conjunction with the study's PIs, and make appropriate referrals as needed.

Age 4: Alternate Mode Protocol and Follow Up Visits

All participants will have the opportunity to participant in a university-based visit as described above.

Phone- or Zoom-based data collection

In some special circumstances, alternate arrangements will be made with participants to complete data collection via phone or Zoom if it is not feasible for them to come to the university lab, or to split data collection to administer the survey via phone and collect other measures in person at another time. For those who decline the university-based data collection but accept an alternate mode, or alternatively opt to take the survey via phone and come in at a later time for the lab visit, the following components will be offered, and reimbursement will be based on completed component (note that the estimated completion time for phone- or Zoom-based data collection is shorter than the lab-based data collection, and reimbursement rates have been adjusted accordingly):

- Maternal survey

See chart *Guidelines for Travel Allowance, Follow-up Visits, and Enhanced Financial Incentives for Data Collection* at the end of the Age 4 Protocol section for reimbursement rates.

Follow up visits

In some special cases, families will be invited back to the university lab for follow up data collection.

Follow-up visit eligibility criteria:

- Equipment malfunction (e.g., wifi issues, device or wiring issues);
- Experimenter error;
- Target child or sibling interruption (e.g. during the maternal survey);
- Family time constraint;
- Participant medical issue;
- Unexpected environmental event; and/or
- Temporary hair style (i.e. that would prevent cortisol collection or EEG).

When one or more of the above criteria is met, the research team will determine the feasibility of inviting participant to a follow-up visit or call.

See chart *Guidelines for Travel Allowance, Follow-up Visits, and Enhanced Financial Incentives for Data Collection* at the end of the Age 4 Protocol section for reimbursement rates.

Data collection at alternative locations

a. In some limited circumstances, as preferred by participants, data collection will take place away from the university labs and at alternate locations, such as libraries and rented office spaces, or in participants' homes. All measures except for EEG and BMI will be administered when data collection takes place at an alternate location. Participants will be compensated at the same rate as the lab visit. All Covid-19 safety protocols will be followed. The alternative locations must follow the criteria below to ensure privacy and security for data collection:
   i. **For alternate locations that are not participant's home:** The selected rooms will be isolated from outside noise; and sound proof, so that the conversations in these rooms will remain private and secure. Any windows will be covered for privacy. Participants will be greeted outside and no signs about the research will be posted publicly.
   ii. **For alternate locations that are participant's home:** if there is no private room for participant to respond to Maternal Survey, we will offer to have the survey responded via phone at another time. Compensation would follow the chart *Guidelines for Travel Allowance, Follow-up Visits, and Enhanced Financial Incentives for Data Collection* at the end of the Age 4 Protocol section for reimbursement rates.
   iii. **In any location:** Data will be collected on password-protected laptops, transmitted to the same Teachers College servers used by site-based visits, using a VPN protocol.

BFY Age 4 In-Person Locating, as needed only

In-person locating will be conducted during the Age 4 wave in order to help find respondents that may be unreachable for their BFY Age 4 interview via other mechanisms. Researchers will have exhausted all methods of locating these participants (e.g., calling, texting, emailing, database searches). Our last resort to keep the respondents in the study is to complete an in-person locating visit in an attempt to reach the respondent. The main goals of the visit are to:

6. Visit the best last-known address on file for the respondent
7. Speak with the respondent (if available) or the informant who opens the door
8. Obtain updated contact information for participants (address, cell phone, emails, or Contact Persons);
9. Schedule an appointment for the Age 4 visit (if speaking with the participant)
10. In some cases, interviewers may also deliver post-interview payments in cash to families.

TEACHERS COLLEGE
COLUMBIA UNIVERSITY
Institutional Review Board
Box 151 | 525 West 120th St. New York, NY 10027
212-678-4105 | RH 13 | IRB@tc.edu | tc.edu/IRB

Guidelines for Travel Allowance, Follow-up Visits, and Enhanced Financial Incentives for Data Collection

	**Data collection mode and travel allowances**				**Enhanced financial incentive for data completion**		
	**University-based data collection**		**Phone plus Zoom[1] option for survey, MEFS, epigenetics**	**Follow up visit for data collection (additional eligibility requirements apply)[2]**	**Keeping first appointment**	**End Game Incentive: Child ≥ 51 months OR data collection end-date is <4 weeks away**	**Incentive for keeping final appointment: Rescheduled >3 times OR Missed > 2 appointments[3]**
**Research incentive**	**$200[4]**		**Based on components completed:**<br><br>**Survey: $100**<br>**M MEFS: $10**<br>**Epi: $10**	**Based on components completed:**<br><br>**Phone Survey only: $50**<br><br>**One or more of the following: $50 + transportation**<br>- **Survey**<br>- **EEG**<br>- **Epi**<br>- **Child battery**<br>- **LENA+PCI**<br>- **MEFS**	**+ $10**	**+ $50**	**+ $50**
**Transportation Allowances**							
< 3 hours by car from initial assigned site	Mileage reimbursement or car service				X	X	X
< 3 hours by car from alternative site	Mileage reimbursement or car service				X	X	X
≥ 3 hours by car to initial or alternative site (options will be offered in the order given)	(Option 1) Mileage reimbursement; 2 nights at hotel (1 rm); 5-6 meals[5]; fun activity for children. 2 adults/2 children max. Offer to everyone in this category.		(Option 2) See above for details.		X	X	X

1. If possible for mom, no devices will be sent

2. Follow-up visit eligibility criteria: equipment malfunction (e.g., wifi issues, device or wiring issues); experimenter error; target child or sibling interruption (e.g. during the maternal survey); family time constraint; participant medical issue; unexpected environmental event; and/or temporary hair style (e.g. that would prevent cortisol collection or EEG). When one or more criteria is met, the research team will determine the feasibility of inviting participant to a follow-up visit or call.
3. Respondents who reschedule or cancel appointment for the 2nd time or respondents who have had two no shows
4. Note: if the participant is willing to come to the university for the remainder of data collection after the phone-based survey has already been administered, then the incentive payment for the visit will be adjusted to $150 (plus any additional incentives for which the participant qualifies.
5. Cost cap is $15/person/meal

Ages 6 and 8 Data Collection

At ages 6 and 8, participants recruited at Baseline will be asked to participate in lab visits around their child's birthday. Lab visits will take place in local university labs by locally hired research staff in the four study sites (see section 9 of this protocol for more details about partnering institutions). The measures that will be assessed can be found in section 13. More details about the data collection procedures at ages 6 and 8 will be submitted to the IRB for review closer to the fielding periods.

Box 151 | 525 West 120th St. New York, NY 10027
212-678-4105 | RH 13 | IRB@tc.edu | tc.edu/IRB

11.	Please check the box(s) that best describes the specific nature of your data.

In Microsoft Word, double-click the box or type an "X" to mark your selection.	
☐ I will personally collect new data.	☐ I will access existing data.
☒ Somebody else will collect the data via proxy (*please explain*). *Baseline through age 3:* All data collection will be conducted by U-M SRC interviewers. *Age 4, 6 and 8:* All data collection will be completed by local, lab-based teams. See p. 10-11 for more information.	☐ I will use a web-based data collection site (*e.g., Amazon's Mechanical Turk (MTurk) or ResearchMatch*).
☐ Other (*please explain*).	

12.	Please check the box(s) that best describe your study activities.

In Microsoft Word, double-click the box or type an "X" to mark your selection.	
☒ Audio recordings	☒ Clinical trials, Experiments, or Randomized Controlled Trials
☒ Documents and Records	☐ Ethnographies, Oral History, and Case Studies
☐ Interviews or Focus Group Sessions	☒ Online (*e.g., Qualtrics, RedCap, or other web-based collection method*)
☒ Observations	☐ Program Evaluations
☒ Other (*please explain*) Biometric collection (hair cortisol,	☒ Video recordings

epigenetics), infant EEG	

13. Please list the *activity*, *occurrence,* and *duration* in which your participants will be engaging.

**Name of Task or Procedure (*e.g., individual audio recorded interview*)**	**Number of occurrences (*e.g., twice*)**	**Activity Duration (*e.g., 30 minutes, each time*)**	**Total time per participant (*e.g., one day, two interviews, 60 minutes total*)**	**Describe the data collected**
**Baseline Survey**	Once	60 minutes	60 minutes	At time of recruitment, a baseline survey will be conducted to obtain information about demographic characteristics, economic habits, maternal health/mental health, housing, and other family process constructs.
**Age 1 home visit**	Once	120 minutes	120 minutes	A phone interview or home visit will be conducted with families in order to collect information about child health and general developmental milestones, as well as family process

				measures.
**Age 2 home visit**	Once	120 minutes	120 minutes	At age 2, an in-home visit was planned to be conducted to collect additional family process data, as well as to collect data from naturalistic observations, with questions related to language development, mother-child behavioral interaction quality, and generalized child development information. Because of the Covid-19 pandemic, data collection was limited to a phone survey.
**Age 3 maternal survey**	Once	60 - 90 minutes	60-90 minutes	At age 3, a phone interview will again collect family process information
**Age 4 lab visit**	Once	150-180 minutes	150-180 minutes	At age 4, the primary focus of the lab visit will be to collect measures of children's cognitive, behavioral, and brain functioning in a host of tasks.
**Age 6 lab visit**	Once	150-180 minutes	150-180 minutes	At age 6, the primary focus of

				the lab visit will be to collect measures of children's cognitive, behavioral, and brain functioning in a host of tasks.
**Age 8 lab visit**	Once	150-180 minutes	150-180 minutes	At age 8, the primary focus of the lab visit will be to collect measures of children's cognitive, behavioral, and brain functioning in a host of tasks.

Total hours of participation for all tasks: 15.5
Total duration of participation (*e.g., days, months, and/or years*): 8 years

14. If you will be audio/video recording, please state how you will ensure that all participants have consented to be recorded. How will you ensure that individuals who are not participating in your study (*e.g., other children in a classroom*) will not also be recorded?

Video/audiotaping:

Following standard SRC procedures, portions of the interviews will be recorded for quality control purposes only. Recordings are encrypted and are transmitted to SRC home offices via secure FTP. All recordings will be securely destroyed at the end of the study. Respondents are read a statement regarding recording at the beginning of the survey and recording is disabled if the respondent does not want to be recorded.

Subjects will be videotaped during naturalistic interactions during the home visit at age 2, as well as during the laboratory visit at age 3. Consent will be obtained on the day of the visit on Consent Addendum forms (forthcoming prior to data collection) for video/audiotaping procedures.

Videos will be stored on secure drives by the SRC and only viewable by researchers at SRC, the research staff of the site PIs, and the research staff of the core study PIs. Videos will be deidentified prior to storage, coded, and stored indefinitely at the University of Wisconsin, Madison following the deidentifying protocol described below.

Interviewers ensure that only the mother and child, as listed on the consent form, are playing at a designated area of the room, with no other individuals.

15. State whether participants will be compensated for their participation. ***NOTE:*** *If you plan to use a lottery system, please state odds of winning here and in the consent form. Also, if you will be offering course credit for study participation, you must discuss this here and include the alternative assignment for those who decline to participate in the study. Will compensation be pro-rated if the participant does not complete all aspects of the study? If you pay participants after their participation, please make it clear how you will link names/contact information confidentially to any record of the compensation.*

All participants will receive $50 for the baseline interview and $100 for the age 1, age 2, and age 3 interviews. For the age 4 laboratory visits, they will receive a larger compensation of up to $310, plus costs for transportation to the laboratory. Compensation will be provided in cash or by check or gift card.

Please note: the monthly payments given to participants are NOT considered subject compensation, but rather compose the intervention condition. They will receive these payments independent of research participation as described below.

Detailed compensation for each phase of the study is listed below:

- Phase 1 Pilot Testing: Baby's First Years Baseline Survey
  - Moms participating will receive $50 for completing the hour survey and $30 or a car service for transportation costs.
- Phase 2 Pilot Testing (site-based): Baby's First Years Age 1 Visit
  - Mothers participating in this study will receive $50 for participating in the visit. Compensation is not contingent on completing particular components of the visit, and families can choose to stop participating at any time
- Age 1 compensation: Mothers will receive $50 before the visit/interview and $50 upon completion of the visit/interview, for a total of $100. Two additional $50 incentive payments are mutually exclusive. Along with the original $100 payment for participation that is offered to all participants, the total possible incentives add up to a total of $150. Here is an outline of all of the incentive payments: See guidelines for compensation below.
  - (to all participants) $50 mailed in advance of the visit
  - (to all participants) $50 given upon visit completion
  - (to select participants) $50 to keep appointment for those who habitually break appointments
    - Eligibility: Respondents who reschedule or cancel appointment for the 2nd time or respondents who have had one no-show.

- (to select participants) $50 to participants whose children are approaching age 2
  - Eligibility: respondents whose child is 15 months or older Age 2 compensation: The token of appreciation for participating in BFY is $100, with some cases up to $200 (see "Monitoring cooperation and increasing response rates "for age 2 data collection).
- Age 2 compensation: The token of appreciation for participating in BFY is $100, with some cases up to $200 (see "Monitoring cooperation and increasing response rates" for age 2 data collection.
- Age 3 compensation: see "Age 3 protocol"
- Age 4 compensation: Participants will receive up to $310 in cash in appreciation for their time, plus transportation support, a meal, and a small gift for the child. In some cases we may increase the token of appreciation for participating in BFY from $200 up to $310, according to the criteria outlined in the Age 4 Protocol section, "Guidelines for Travel Allowance, Follow-up Visits, and Enhanced Financial Incentives for Data Collection" chart.
- Age 6 and 8 compensation will be defined closer to these data collection points.

16. Will deception be used? If so, please provide a rationale for its use. ***NOTE:*** *Upload a debriefing script as a separate document. Include a statement that gives your participants the opportunity to withdraw their participation at that time. Studies involving deception are given Full Board Review unless the deception is minor and risks are minimal.*

N/A

17. Will you have a control group, or a comparison group? If so, please describe your procedures and explain the purpose of using a control group.

Upon enrollment, participants are randomly assigned to either the experimental group (40% - $333) or the control group (60% - $20/month). Both groups will receive 76 monthly cash gifts. Upon enrollment, the interviewer activated the debit card for the participant, assigned a pin number, and provide instructions about its use during recruitment. Participants received their first payment on the card at this time.

18. Will you need bilingual interpreters or interviewers, and if so, what will you do to ensure participant confidentiality? What are your procedures for recruiting interpreters and interviewers?

Yes, about 25-30% of the sample is Spanish speaking. Bilingual interviewers were hired by U-M Survey and Research Center.

Data collection: Whenever possible, interviews will be conducted by bilingual research staff. As such, they will be trained on the full study protocols as well as confidentiality and HIPAA procedures in both English and Spanish, and will be fully aware of the proper ways to maintain subject confidentiality. They will be recruited at the discretion of SRC. They will be screened based on bilingualism and prior experience conducting research and/or working with similar

populations. Occasionally, in sites with a smaller bilingual population, it may be necessary to hire some monolingual English speaking interviewers. However, every effort will be made to have a bilingual interviewer available when needed.

Debit card support: A phone and email helpdesk will be established to support participants in the use of their debit cards. The helpdesk will be staffed by bilingual research assistants and other staff. In some limited circumstances when bilingual support is not available, we will contact a translator at Pacific Interpreters, who will facilitate communication. Pacific Interpreters is a comprehensive language services providers widely used within medical practices

SECTION III: DESCRIPTION OF RESEARCH RISKS & BENEFITS

19. Describe the potential risks to your participants. Risks can be physical, psychological, economic, or social. What is the likelihood of these risks occurring, and/or their seriousness (*e.g., exposure of sensitive data*)? How will you work to minimize these risks? ***NOTE:*** *The IRB regards no research involving human participants as risk-free. You may describe minimal risks for your study (such as discomfort, boredom, fatigue, etc.), or state that the research will involve minimal risk, similar to an activity (named) that participants would perform in their daily lives.*

This study includes less than minimal risk to children and adults. We have determined that it is not necessary to attain assent from children at this young age for minimal participation.

The primary potential risk of this study for adults is discomfort with some of the questions, or boredom during lengthy questionnaire administration. Similarly, children may experience boredom, frustration, or discomfort during parent-child interaction or in-lab cognitive/neural assessment tasks.

Another risk, as in most studies, is a loss of confidentiality, in the event of a lost computer, security breech, or other similar occurrence, but all precautions will be taken to protect all personal information (see Confidentiality procedures & Participant Privacy).

Another potential risk to parents is that a debit card could be lost/stolen. If the card is lost/stolen, the card will be deactivated and the participant will be furnished with a new card by a member of the research team at no cost. Alternatively, they can request a new card directly from Greenphire for a $7 fee. If the card is stolen with knowledge of the PIN code, the participant must report it to Greenphire, and the fraudulent charges will be reimbursed to the new card. The incidence of loss or theft was relatively low in the pilot study (11 instances for 31 participants, none of whom had fraudulent charges).

One major risk of this study would be that the cash gift could increase income, thereby leading to a loss of social service benefits. However, to avoid this risk we will only enroll mothers in sites where we have secured the appropriate exemptions or approvals, ensuring that the mothers will not lose

eligibility for public benefits as a result of our cash transfer. We were aware of the complexity of these tasks when sites were selected. .Since the gift is under $14,000 a year and is being given with no expectation for goods or services in return, it is not taxable and should not be reported as income on tax returns. (See Memo from Legal Consultant.) As a result, this gift does not count towards participants' eligibility for government support programs that use modified adjusted gross income in their calculations, including and not limited to Medicaid and Head Start, but with the exception of Social Security Income (SSI), Section 8 Housing Assistance, and Women, Infants, and Children (WIC) Food and Nutrition Service. We have secured federal agency support from the U.S. Department of Health and Human Services, indicating that these determinations can be left to the states. (see attached letter from DHHS). See Appendix A for information of clawback implementation at each site and copy of site specific letters that will be given to participants to decide if they would like to sign up to receive the gift.

It is possible that the intervention may result in harm rather than benefit to the families. The protocol described below is consistent with standard practices of a clinical trial.

- If we determine that receipt of a predictable monthly cash gift is somehow harmful for many of the children or families in our study, then we will inform you of these findings, stop the research study, and give you the choice of whether or not to continue receiving the monthly cash gift. We will define "harmful" as significant negative effects on 75% of the maternal mental health outcomes we measure when your child is 12 or 24 months of age, and/or 75% of child cognitive outcomes when your child is 24 months of age.

- In the unlikely situation that the principal investigator team believes that the cash gift is causing harm to an individual child or mother, we would discontinue the payments. We would make every effort not to do so against the mother's wishes.

20. What are your plans for ensuring necessary intervention in the event of a distressed participant and/or your referral sources if there is a need for psychological and/or physical treatment/assistance?

If a participant is distressed or asks to stop participation at any time, we will accommodate their request by terminating whatever portion of participation is causing distress.

21. What qualifications and preparations enable you to estimate and minimize risk to participants?

A pilot study was conducted under another protocol in 2014 that tested feasibility of using debit cards for unconditional cash transfers (UCTs). It also tested the feasibility of collecting the initial data set. The pilot informed the design of Baby's First Years, to ensure that participants' information ins protected.

22. Describe any possible direct benefits to your participants. Most research will not have any direct benefits to participants. Occasionally, a study design will include a diagnosis, evaluation, screening, counseling or training, etc., that has a concrete benefit to participants, independent of the nature or results of a research study.

Participants may derive benefit from the financial components of this study, particularly those who are randomized into the higher payment condition. We expect that these participants may experience some benefits in terms of financial security and quality of life. Other participants may derive some benefit from the smaller monetary compensation they receive. No other direct benefits are foreseen from this study.

SECTION IV: CONFIDENTIALITY PROCEDURES & PARTICIPANT PRIVACY

23. Please check the box(s) that best describes your data. ***Note:*** *Sensitive data potentially poses substantial threat to research subjects and can become problematic for the researcher, researched collection, and/or the dissemination of research data (Lee & Renzetti, 1990). Substantial threat may include threat to reputation, employment, or access to resources. Sensitive data may include studies of domestic violence, immigration status, political activism, homicide, death, trauma, assault, and/or mental, sexual, or physical health* (Lee, R. M., & Renzetti, C. M. (1990). The problems of researching sensitive topics: An overview and introduction. *The American Behavioral Scientist, 33*(5), 510–528).

☐ Completely anonymous data (*both sensitive and non-sensitive*)	☐ Non-sensitive data with identifiers
☒ Sensitive data with identifiers	☐ Other (*please explain*)

24. For data with identifiers please describe your method for de-identifying the data to maintain confidentiality. ***Note:*** *The term de-identified data refers to subject data from which all information that could reasonably be used to identify the subject has been removed or replaced. For example, the researcher may use the safe-harbor method to remove specified identifiers (name, address, phone, or any other unique identifier, etc.) from a dataset; the partially de-identified method to remove most, but not all identifiers from the data set (may require a data use agreement); or the generation of variables method to replace study subjects' identifiers, like using a unique code or pseudonym. To be truly de-identified data, the investigator cannot have codes that link to identifiers.*

Identifiable information:

Research staff will be collecting participant names, contact information, birth dates, and social security numbers. SSNs will be entered by Research staff who will also, given consent, have access to administrative records from selected agencies, including TANF, food stamps, child care, and WIC (see Transaction and Administrative Records Consent Form).

Interviewers will ask participants to provide name, address, email, and phone number for contact persons who may help the research team locate primary participants for re-interview in Waves 1 - 4. Information for those individuals will be attached to the record of the primary participant and referenced by the primary participant unique identifier.

Contact persons will not be asked for their consent to provide this information. Their identifiable information will be secured with the same level of protection afforded to the identifiable information of the primary research participant and used solely for the purposes of locating primary research participants to invite them to participate in the subsequent study waves/years.

All information collected from participants is completely confidential, and is only accessible by the research team and IRB. Greenphire, Inc. (the organization managing the debit cards) has protocols in place to ensure data security and compliance with HIPAA regulations. Greenphire, Inc. will have access to basic PII (name, phone number with permission, etc.), and has security protocols in place to protect participant privacy, which utilize web-based encryption SSL, field level encryption and firewalls for its web and application servers (see attached "Greenphire Application Model," page 7, section 5). Participants will be informed of state law regarding mandated reporters for child abuse and neglect.

Deidentification of study data:

Personal identifiers collected during the Baseline interview will be retained for the duration of the study to allow re-contact in future waves. Links between the data and identifying information will be made only on secure data files that are used by research staff to process, edit, and clean the data. These data are stored on internal servers at University of Michigan behind firewalls and are password protected and accessible only by staff who need the data for processing, editing, and cleaning, and have signed a Pledge of Confidentiality as a condition of their employment at the University of Michigan Institute for Social Research (UM ISR).

University of Michigan will periodically provide data to the study Principal Investigators at University of Wisconsin-Madison. Data containing identifiers will be encrypted and provided to via secure file transfer protocol.

All information will be securely transferred to the University of Wisconsin Madison. The data, and video/audio recordings will be deidentified and stored according UWM data storage protocol described below.

Social security numbers:

Age 1 and Age 2: Collected SSN will be encrypted at the time of the interview and will be only decrypted once received by University of Wisconsin and stored on secured project servers.

*Age 4: (*Nebraska only): SSN will be collected on a hard copy form. The completed form will be scanned and uploaded onto TC's secure server. The physical forms will be kept in a locked cabinet in a locked room, and then mailed through secure shipping to UWM. They will be scanned and stored according UWM data storage protocol described below.

Data containing identifying information are stored and remain on the secure project servers (SILO) at the Social Science Computing Cooperative in the Social Science building at the University of Wisconsin-Madison Institute for Research on Poverty (IRP). Access is maintained on a server which is appropriate for the use of fully-identified PHI (HIPAA-protected data). Access is restricted to programming staff (8). This environment requires two-factor authentication and cannot access the internet or local computer drives, including those used for removable media devices. Files containing identifiers are encrypted when stored (not in immediate use).

Access to the research data (use is restricted) is limited to those study team members who have completed data security training and have signed a confidentiality agreement. Researcher also uses SILO, on servers that are appropriate for Limited Data Sets as defined by HIPAA and data with similar security requirements. Researchers must also use two-factor authentication and can only remove files containing aggregate information from this server.

IRP data security training requires that data access individuals sign a confidentiality agreement and complete a checklist of understanding. These documents are renewed on an annual basis. They must sign a SILO Access Agreement with the Social Science Computing Cooperative (SSCC).

To protect the confidentiality of subjects, the data used for analysis (Research Data) provided to the study team has personal identifiers stripped, ID numbers masked, and dates rounded to MM/YYYY.

Data from other sites will be delivered to IRP programmers for linkage administrative data records via secure file transfer protocol. Only IRP programmers will have access to identifying information linked across data sources.

All participants will be assigned subject ID numbers. All paper and electronic documents will be de-identified, removing names and other identifiable information, and replaced with the ID numbers.

Additionally, we have a Certificate of Confidentiality from the U. S. Department of Health and Human Services for this study. This certificate helps to protect participant's privacy by not allowing the research team to tell people who are not part of the study about the participant's participation. This includes courts and the police. University and government officials who are responsible for monitoring this study may inspect records, but they will not be able to identify the participant, their child, or family.

Age 4 Confidentiality Procedures & Participant Privacy

All information will be securely transferred from a VPN-protected Teachers College server to the University of Wisconsin Madison. The data, and video/audio recordings will be deidentified and stored according UWM data storage protocol described in this section.

25. If you are working with sensitive identifiable data, please explain why identifiers are necessary to carry out your research. Sensitive identifiable data should never be sent as an email attachment. ***NOTE:*** *If you are collecting private, identifiable heath information as part of your research, please see our website* ***www.tc.edu/irb*** *under Forms and Guidelines for the Health Insurance Portability and Accountability Act (HIPAA) document.*

Names in combination with social security numbers: Respondents who provided Administrative Consent but their Social Security Number was invalid will be asked to update their SSN. SSN is immediately encrypted upon entering in the survey instrument and can only be decrypted once transmitted to the secure data enclave. SSNs will be entered Research staff who will also, given consent, have access to administrative records from selected agencies, including TANF, food stamps, child care, and WIC (see Transaction and Administrative Records Consent Form).

Age 4 wave: Nebraska's Department of Health and Human Services (DHHS) requires that their Authorization for Disclosure of Protected Health Information form be completed in order to release administrative records of participants, as described above. The completed form will be scanned and uploaded onto TC's secure server. Digital (scanned) copies will be deleted from the local computer and scanner after upload. The physical form will be kept in a locked cabinet in a locked room. Once data collection is complete for all participants, the physical forms will be mailed through secure shipping to Dr. Katherine Magnuson at University of Wisconsin-Madison, School of Social Work. They will be scanned and deposited on University of Wisconsin servers, and stored in the BFY Data Repository maintained by the University of Wisconsin.

SECTION V: DATA SECURITY

26. Please respond to the following sections.

Please check the box(s) that best describes how you will transfer your data.	
☒ I will use a Virtual Private Network	☐ Not applicable (*e.g., data will not be*

(VPN) for secure data transfer or other form of encryption (*e.g., Teachers College's secure remote network access*).	*accessed remotely or transferred. It will exist only on a locally stored password-protected hard drive*).

**Please check the box(s) that best describes how you will store your data.**	
☐ On a password-protected local or external computer hard drive.	☐ On Teachers College's local password-protected network.
☐ On Teachers College's Google Drive, in a password-protected folder.	☐ In Teachers College's Dropbox (*faculty only*).
☒ Other (*e.g., cloud-based, password-protected storage*) (*please explain*).<br>*Baseline through age 3:* The Survey Research Center at the University of Michigan will be leading the data collection. The research team uses an approved protocol for maintaining the security of participants' data.<br><br>All participants will be assigned subject ID numbers. Respondent's personal information obtained by UM SRC will be collected on password-protected and encrypted laptops, transmitted to UM SRC/ISR servers using a VPN protocol, and then stored on secured UM SRC/ISR servers. Study electronic records will be accessible only to the approved UM SRC study staff.<br><br>Periodically, data collected by UM SRC will be transferred to the research team using SFTP transfer protocols.<br><br>Age 4: The research teams at Teachers College Columbia University, the University of New Orleans, the University of Nebraska at Lincoln, and the University of Minnesota use an approved protocol for maintaining the security of participants' data.<br><br>Respondent's personal information obtained by	

Box 151 | 525 West 120th St. New York, NY 10027
212-678-4105 | RH 13 | IRB@tc.edu | tc.edu/IRB

the team will be collected on password-protected and encrypted laptops, transmitted to TCCU servers using a VPN protocol, and then stored on a secured TCCU server. Study electronic records will be accessible only to the approved study staff.<br><br>Periodically, data collected by staff will be transferred to the University of Wisconsin, Madison servers using SFTP transfer protocols.<br><br>The database that links identifiers to participant names will be stored with highest security at the University of Wisconsin, Madison, supervised by one of our Core Principal Investigators, Professors, Katherine Magnuson, Ph.D.. The Wisconsin site will act as a repository for all survey, administrative, home visit and lab visit data. All datasets will be de-identified by University of Wisconsin before being used for substantive analysis by the external PIs from UCI, TCCU, NYU, CUMC, and other investigators external to University of Michigan.<br><br>Debit card information: Information used to sign on for the debit card will be protected by Greenphire.. Greenphire, Inc. will have access to basic PII (name, phone number with permission, etc.), and has security protocols in place to protect participant privacy, which utilize web-based encryption SSL, field level encryption and firewalls for its web and application servers.<br><br>More information on the UWM security protocols can be found in section IV.	

**Please check the box(s) to affirm your data security plans.**			
☒	I will encrypt my data (e.g., *conceal data by converting it into a code*).	☒	I will use anti malware protections and automatic software updates.
☒	I will block unauthorized access to my data (*e.g., firewall*).	☒	I will disable file and media sharing if I do not need it.

☒ I will delete old files from cloud-based backups and local hard drives.	☒ I will take care of privacy settings immediately upon setup.

27. Teachers College classifies all data associated with ongoing research studies as confidential, meaning only project staff, academic advisors, collaborators and other individuals at the college on a need-to-know basis may have access to it. Confidential data at a minimum should be stored on a password-protected computer, or in a password-protected file or folder if the computer is shared. Paper and other physical media should be kept under lock and key. All computers accessing data should have anti-virus software installed.

**Please check the box below:**
☒ Yes, I acknowledge and understand how Teachers College classifies research data.

SECTION VI: INFORMED CONSENT PROCEDURES

Informed consent is a process, not just a form.

28. What are your procedures for obtaining a participant's informed consent to take part in the research?

Researchers will obtain signed consent in person at the time of recruitment to the study using a consent form. Bilingual research assistants obtain consent in English or in Spanish, depending on preferred language of the participant. The purposes and procedures of the study will first be explained in lay language, and the parent will have the opportunity to have any questions answered before signing. The researcher will explain the study in detail and then will offer to read the full consent form (Research Participation Consent Form) to the mother if she desires, or she will have the opportunity to read it herself. It will be made clear that she can withdraw participation at any time with no consequences. The researcher then will explain that as part of conducting research in a hospital setting she will need to complete a HIPAA authorization to complete the survey.

A separate form (Cash Gift Letter) will be signed by participants agreeing to participate in the cash gift. This will describe the duration of the gift, efforts in place to secure eligibility to government support services, and information about the use of the debit card. This form also allows participants to opt-out of receiving monthly text message or email reminders (provided by Greenphire) pertaining to account balances. Participants will receive a debriefing form where explaining the purpose of the study is to gain an understanding of how receiving a cash gift might affect the experiences of families with new babies. Specifically, the effects of the cash gift on family life and child development.

In addition, participants are able to indicate whether they are willing to participate in research on how the money is spent via spending tracking and disclosing their Social Security Number in order for researchers to access administrative records. This form will be administered in the same way as the Transaction and Administrative Records Consent Form. For this form, it will be made clear that the participant can choose to not participate in or withdraw from research activities at any time while still receiving the monthly compensation earned in the cash gift. That is, receiving the monthly payment is not (and continues not to be) contingent upon research participation (or vice versa).

All consent forms will be administered via an Electronic Document Utility nested in Blaise, a computer-assisted interview software tool. Signatures will be obtained electronically. Participants will receive paper copies of all consent forms and the gift letter for their records.

Research will be described verbally in plain language to potential subjects. Participants also will receive a package of materials that explains participation components in plainer language.

Consent and understanding is determined throughout the informed consent and other participation processes, by asking questions like “do you understand?,” “is that okay?,” and “do you have any questions?” at multiple points throughout the process.

Age 4: See details for the age 4 consent procedures on pages 51-53.

29. How will you describe your research to potential participants?

A recruitment brochure was given to potential participants. It included study information as well as a brief description on what the study is about: “The goal of this research study is to gain an understanding of the family experiences and backgrounds of families with new babies. We hope that examining these factors during the first three years of child’s life will help us understand how they influence how children learn and grow, and how programs can support families during the first years of their child’s life.” This brochure is under modification #1.

30. What will you do to ensure participants’ understanding of the study and what it involves?

Data collectors will explain the research in plain language and ask the participant at multiple points if she understands, or have any questions. Research does not continue until mother confirms her understanding of the research and what is being asked of her.

31. Use this section to provide a request for a full or partial waiver of informed consent, and justify this request. Indicate “*not applicable*,” if you are not requesting a waiver. ***Note:*** *You may cite criteria from the following link regarding Federal regulations and guidelines:* **www.hhs.gov/ohrp/humansubjects/guidance/45cfr46.html#46.116**

N/A

TEACHERS COLLEGE
COLUMBIA UNIVERSITY
Institutional Review Board
Box 151 | 525 West 120th St. New York, NY 10027
212-678-4105 | RH 13 | IRB@tc.edu | tc.edu/IRB

Note for Researchers: *Templates are available in Mentor/Documentation. Drafts of forms will not be accepted. Please proofread all files.*
