# Supplementary material for: Unconditional Cash Transfers and Maternal Assessments of Children's Health, Nutrition, and Sleep: A Randomized Clinical Trial
Source: JAMA Netw Open. 2023 Sep 29;6(9):e2335237. doi: 10.1001/jamanetworkopen.2023.35237 (PMC10543132; doi:10.1001/jamanetworkopen.2023.35237)
Supplement: Supplement 2. — eMethods. eReferences eTable 1. Marginal Effects of the Cash Gift on Dichotomous Outcomes eTable 2. Descriptive Statistics and Treatment Impacts of the Child Health Index eTable 3. Intent-to-Treat (ITT) Impacts of Unconditional Cash Transfer on Global Measures of Child Health, Nutrition, Sleep, and Health Care Utilization eTable 4. Factor Loadings for a 2-Factor Solution of the Child Health Index Items eTable 5. Findings From Current Published Papers, Working Papers, and Preprints Reporting BFY Impacts eFigure. Flow Diagram for Baseline, Age-1, Age-2, and Age-3 Data Collection [file jamanetwopen-e2335237-s002.pdf]

Supplemental Online Content

Sperber JF, Gennetian LA, Hart ER, et al. Unconditional cash transfers and maternal assessments of children's health, nutrition, and sleep: a randomized clinical trial. *JAMA Netw Open.* 2023;6(9):e2335237. doi:10.1001/jamanetworkopen.2023.35237

eMethods

eReferences

eTable 1. Marginal Effects of the Cash Gift on Dichotomous Outcomes

eTable 2. Descriptive Statistics and Treatment Impacts of the Child Health Index

eTable 3. Intent-to-Treat (ITT) Impacts of Unconditional Cash Transfer on Global Measures of Child Health, Nutrition, Sleep, and Health Care Utilization

eTable 4. Factor Loadings for a 2-Factor Solution of the Child Health Index Items

eTable 5. Findings From Current Published Papers, Working Papers, and Preprints Reporting BFY Impacts

eFigure. Flow Diagram for Baseline, Age-1, Age-2, and Age-3 Data Collection

This supplemental material has been provided by the authors to give readers additional information about their work.

© 2023 Sperber JF et al. *JAMA Network Open.*

eMethods

The specific amount for the cash gift was motivated by quasi-experimental work which finds a $4,000 increase in annual income (adjusted for inflation) during the first few years of life to be associated with significant boosts to children's academic achievement[1,2] Additionally, this amount aligns with social services many low-income families are eligible for (e.g., the Earned Income Tax Credit), highlighting the policy relevance of the study design.

At the time of recruitment, the distribution of the cash gifts was planned for the first 40 months of the children's lives. The duration of the monthly cash gifts was subsequently extended twice. First, in response to the need to postpone in-person data collection due to the COVID-19 pandemic, the cash gifts were extended for an additional year, through 52 months. Subsequently, motivated by the evidence that the likelihood of adverse outcomes increases the more time a child experiences poverty, additional philanthropic funding extended the monthly cash transfers to 76 months.

Prior to the launch of the study, we secured approvals from state or local officials to ensure that participants would not lose eligibility for public benefits because of the cash gift, including Medicaid.[3] A 40/60 division of the cash gifts across the sample was used to reduce study costs while also maintaining sufficient statistical power. We have summarized the currently published impact findings of this study in eTable 5.

Exploratory Analyses

We conducted exploratory analyses using a composite measure of each outcome of interest. The composites were formed by standardizing the outcomes in that domain, averaging them together, and then re-standardizing. Standardization was performed separately at each wave of data collection, across both high-cash and low-cash gift groups. Higher values indicate poorer outcomes in that domain.

Global Nutrition. Global nutrition consists of the servings of unhealthy and healthy (reverse coded) foods consumed each day.

Global Sleep. Global sleep consists of the PROMIS-Sleep Disturbance Scale and two items relating to bedtime routines and consistent bedtimes from the CHAOS scale.

Global Health. Global health consists of the overall health rating and the indicator of disability diagnosis.

Global Healthcare Utilization. Global healthcare utilization consists of the 2+ doctor visits due to illness or injury indicators and the number of ER/urgent care visits

Preregistered Primary Analyses

Child Health Index. The Child Health Index is a pre-registered, primary outcome available in the publicly released dataset. It is an additive index of the following items: maternal rating of the child's overall health status on a Likert scale ranging from 1 (*excellent*) to 5 (*poor*); child disability status or diagnosis with a health condition, indicated by either 1 (*yes*) or 0 (*no*); number of doctor visits due to illness measured by either 0 (*0 – 1 visits*), 1 (*2 – 5 visits*), or 2 (*6+ visits*); number of doctor visits due to injury measured by either 0 (*0 – 1 visits*), 1 (*2 – 5 visits*), or 2 (*6+ visits*); any ER/urgent care visit in the last year indicated by either 1 (*yes*) or 0 (*no*); and the total number of ER visits measured as either 0 (*none*), 1 (*1 visit*), 2 (*2 – 5 visits*), or 3 (*6+ visits*).

Factor Analysis. As part of our pre-registration, we conducted an exploratory factor analysis (EFA) of the five health and healthcare items. We hypothesized the items would load onto either one "health" or two "health" and "healthcare utilization" factors. The child's overall

© 2023 Sperber JF et al. *JAMA Network Open.*

health rating, disability indicator, doctor visits due to illness and injury indicators, and the frequency of ER/urgent care visits were entered into an EFA with oblique oblimin rotation.

eResults

The coefficients below reflect the standardized treatment impact on a respective outcome, divided by the standard deviation (SD) of the control group. Marginal effects of the dichotomous outcomes derived from probit regressions are available in eTable 1.

Poor Bedtime Routines. Bedtime routines were indexed via two items from the Confusion, Hubbub, and Order Scale administered at Age 1 and Age 2 (CHAOS).[21] Participants selected whether the statements "We have an evening bedtime routine" and "My child goes to bed at a regular time" were true (or false) of their home most of the time. We averaged the scores of these two items together, with higher scores indicating fewer sleep routines. Scores were standardized for linear analyses. There was no statistically detectable effects of the cash gift on bedtime routines at Age 1 ($\beta_{Age1} = -0.04$, SE = 0.07, $p = 0.55$) or Age 2, $\beta_{Age2} = -0.01$, SE = 0.07, $p = 0.90$)

Diagnoses. At each age, mothers provided information about their child's specific health conditions and diagnoses. The most common diagnoses at Age 1 included eczema (n=21) and asthma (n=21). At Age 2, the most commonly reported diagnoses were asthma (n=20) and autism (n=9). At age 3, autism (n=28) and asthma (n=20) were again the most common diagnoses, as reported in the main manuscript. There were no statistically detectable effects of the cash gift on the likelihood of reporting a diagnosis of autism ($\beta_{Age3} = 0.01$, SE = 0.01, p = 0.76) or asthma ($\beta_{Age3} = 0.01$, SE = 0.01, $p = 0.91$) at Age 3.

Vaccinations. At each wave of data collection, mothers also responded to a dichotomous indicator of whether the child was up-to-date on routine childhood vaccinations. Between 90 – 94% of all mothers indicated their child was up-to-date on their vaccinations. There was no effect of the cash gift on maternal report of child vaccination status, $\beta_{Age1} = -0.01$, SE = 0.02, p = 0.67; $\beta_{Age2} = 0.01$, SE = 0.02, $p = 0.66$; $\beta_{Age3} = 0.01$, SE = 0.02, $p = 0.51$. We did not have access to medical records to confirm maternal report of vaccination status.

Missed Medical Care. We also surveyed the mothers on missed medical or dental care. Across time points, between 2 – 6% of all mothers reported they had missed needed medical or dental care for themselves or their child in the last year due to cost. In comparison, only 1.2% of caregivers nationally reported delaying medical care for their child due to cost (95% CI 0.9, 1.6; NCHS, 2020). There were no group differences in the likelihood of missing needed medical or dental care at any timepoint, $\beta_{Age1} = -0.01$, SE = 0.02, $p = 0.93$; $\beta_{Age2} = 0.02$, SE = 0.02, p = 0.40; $\beta_{Age3} = -0.01$, SE = 0.02, $p = 0.50$.

Consumption of Cow's Milk. At the Age-2 visit, mothers reported on the frequency with which their child consumed unflavored cow's milk on an average day, with options ranging from 0 (*not at all*) to 5 (*5+ times per day*). On average, mothers reported that their child consumed cow's milk 2.11 times per day (SD = 1.62). There were no statistically detectable effects of the cash gift on the frequency of cow's milk consumption, $\beta_{Age2} = 0.07$, SE = 0.07, p = 0.35

Medicaid Receipt. Finally, at the Age-3 visit, 71% of the sample reported receiving Medicaid. In an exploratory (non-preregistered) analysis, we detected a significantly lower Medicaid enrollment among families in the high-cash gift group. Receiving the high-cash gift resulted in a lower likelihood of being enrolled in Medicaid at Age 3, $\beta_{Age3} = -0.07$, SE = 0.03, p = 0.02. This effect was trending at the Age-1 ($\beta_{Age1} = -0.05$, SE = 0.03, $p = 0.12$) and Age-2 wave, $\beta_{Age2} = -0.05$, SE = 0.03, $p = 0.08$. As noted above, mothers were informed upon enrolling

in the study that because the monthly cash transfers were being given as a gift, they were not taxable income and should not affect their or their child's eligibility for Medicaid.

Child Health Index. eTable 2 presents the descriptive statistics and treatment impacts of the preregistered Poor Health Index at each wave of data collection. We observed no statistically significant effect of the intervention on the Poor Health Index.

Global Measures. No differences were observed by treatment status on any global measure of health, nutrition, sleep, or healthcare utilization at any timepoint (eTable 3).

Factor Analysis. Neither a one- or two-factor solution appropriately fit the data. We observed poor factor loadings (eTable 4). Indeed, the KMO indicated that the variables are poorly related for factor analysis ($KMO_{Age1} = 0.57$, $KMO_{Age2} = 0.57$, $KMO_{Age3} = 0.63$), and the high uniqueness values suggests the variables are not well explained by the factors.

© 2023 Sperber JF et al. *JAMA Network Open.*

eReferences

1. Duncan GJ, Morris PA, Rodrigues C. Does Money Really Matter? Estimating Impacts of Family Income on Young Children's Achievement With Data From Random-Assignment Experiments. *Dev Psychol*. 2011;47(5):1263-1279. doi:10.1037/a0023875

2. Dahl G, Lochner L. The Impact of Family Income on Child Achievement: Evidence from the Earned Income Tax Credit. Published online December 2008. doi:10.3386/w14599

3. Noble KG, Magnuson K, Gennetian LA, et al. Baby's First Years: Design of a Randomized Controlled Trial of Poverty Reduction in the United States. *Pediatrics*. 2021;148(4):e2020049702. doi:10.1542/peds.2020-049702

eTable 1. Marginal Effects of the Cash Gift on Dichotomous Outcomes

	**Age 1**	**Age 2**	**Age 3**	**Cumulative Impacts (Age 1 – 3)**
Health Outcomes				
Diagnosed with health condition or disability	0.03<br>(0.02)	-0.01<br>(0.02)	0.03<br>(0.02)	0.02<br>(0.03)
Healthcare Utilization				
2+ doctor visits due to illness	0.05<br>(0.03)	-0.01<br>(0.03)	-0.01<br>(0.03)	0.01<br>(0.02)
2+ doctor visits due to injury	0.01<br>(0.01)	-0.01<br>(0.01)	-0.02<br>(0.02)	-0.01<br>(0.01)
Non-Preregistered Outcomes				
Up-to-Date on Vaccinations	-0.01<br>(0.02)	0.01<br>(0.02)	0.01<br>(0.02)	0.01<br>(0.01)
Missed Medical/Dental Care	0.01<br>(0.02)	0.02<br>(0.02)	-0.02<br>(0.02)	0.01<br>(0.01)
Medicaid Enrollment	-0.05<br>(0.03)	-0.05+<br>(0.03)	-0.07*<br>(0.03)	-0.06*<br>(0.02)
Autism Diagnosis	N/A	N/A	0.01<br>(0.01)	N/A
Asthma Diagnosis	N/A	N/A	0.01<br>(0.01)	N/A
**Sample Size**	**929**	**919**	**920**	**2,768**

Note. + p<0.10, *p<0.05. Dichotomous outcomes were estimated using logistic regression and the coefficients represent marginal effects. Standard errors are reported in parentheses. Estimates are adjusted for the covariates listed in Table 1, site-based fixed effects, survey administration method (i.e., phone or in-person) at the Age 1 survey, and child age at the time of the assessment.

© 2023 Sperber JF et al. *JAMA Network Open.*

eTable 2. Descriptive Statistics and Treatment Impacts of the Child Health Index

	Mean (SD)		Treatment Impacts
	Low-Cash Gift	*High-Cash Gift*	ES (SE)
Age 1	5.56<br>(2.03)	5.75<br>(2.22)	0.12<br>(0.07)
Age 2	4.91<br>(1.91)	4.93<br>(1.98)	0.02<br>(0.14)
Age 3	4.92<br>(2.12)	4.92<br>(1.96)	0.04<br>(0.07)
**N**	**542 – 547**	**372 – 382**	**919 – 929**

Note. +p<0.10, *p<0.05. The composite is an additive index of the following items: child's health rating ranging from 1 (*excellent*) to 5 (*poor*); child disability status or diagnosis with a health condition, 1 (*yes*) or 0 (*no*); number of doctor visits due to illness, 0 (*0 – 1 visit*), 1 (*2 – 5 visits*), or 2 (*6+ visits*); number of doctor visits due to injury, 0 (*0 – 1 visit*), 1 (*2 – 5 visits*), or 2 (*6+ visits*); any ER/urgent care visit in the last year 1 (*yes*) or 0 (*no*); and the total number of ER visits, 0 (*0 visits*), 1 (*1 visit*), 2 (*2 – 5 visits*), or 3 (*6+ visits*). ES reflects the standardized difference between the two groups, divided by the SD of the control group. Robust standard errors are in parentheses. Estimates are adjusted for the covariates listed in Table 1, site-based fixed effects, survey administration method (i.e., phone or in-person) at the Age 1 survey, and child age at the time of the assessment.

© 2023 Sperber JF et al. *JAMA Network Open.*

eTable 3. Intent-to-Treat (ITT) Impacts of Unconditional Cash Transfer on Global Measures of Child Health, Nutrition, Sleep, and Health Care Utilization

	**Age 1**	**Age 2**	**Age 3**	**Cumulative Impacts (Age 1 – 3)** [a]
	ES (SE)	ES (SE)	ES (SE)	ES (SE)
Global Health [b] (*z*)	0.10<br>(0.07)	0.02<br>(0.07)	0.11<br>(0.07)	0.08<br>(0.05)
Global Nutrition [c] (*z*)	N/A	-0.10<br>(0.07)	N/A	N/A
Global Sleep [d] (*z*)	-0.10<br>(0.07)	0.03<br>(0.07)	N/A	-0.04<br>(0.06)
Global Healthcare Utilization [e] (*z*)	0.10<br>(0.07)	-0.01<br>(0.07)	-0.04<br>(0.07)	0.01<br>(0.05)
**Sample Size**	**929**	**919**	**920**	**2,768**

Note. + $p < 0.10$. Each composite was formed by averaging together the relevant indicators and standardizing the mean, with higher values reflecting poorer outcomes in that domain. ES reflects the standardized difference between the two groups, divided by the SD of the control group. Robust standard errors are in parentheses. Estimates are adjusted for the covariates listed in Table 1, site-based fixed effects, survey administration method (i.e., phone or in-person) at the Age 1 survey, and child age at the time of the assessment.

[a] Cumulative Impacts reflect the estimates of the intervention on the respective outcome, pooled across waves (i.e., ages 1, 2, and 3).

[b] Global health consists of the overall health rating and the indicator of disability diagnosis.

[c] Global nutrition consists of the servings of unhealthy and healthy (reverse coded) foods consumed each day.

[d] Global sleep consists of the PROMIS-Sleep Disturbance Scale and two items relating to bedtime routines and consistent bedtimes from the CHAOS scale. The CHAOS scale was administered only at the Age 1 and Age 2 assessment; therefore, the only available Age 3 sleep measure was the PROMIS-Sleep Disturbance scale, and results for this are presented in Table 3.

[e] Global healthcare utilization consists of the 2+ doctor visits due to illness or injury indicators and the number of ER/urgent care visits.

© 2023 Sperber JF et al. *JAMA Network Open.*

eTable 4. Factor Loadings for a 2-Factor Solution of the Child Health Index Items

	Age 1			Age 2			Age 3		
	Factor 1	*Factor 2*	*Uniqueness*	*Factor 1*	*Factor 2*	*Uniqueness*	*Factor 1*	*Factor 2*	*Uniqueness*
Child Health Rating	-0.06	0.43	0.84	-0.10	0.41	0.86	-0.07	0.46	0.81
Disability Diagnosis	-0.01	0.33	0.89	-0.01	0.37	0.86	-0.05	0.40	0.86
2+ doctor visits due to illness	0.19	0.44	0.69	0.14	0.47	0.69	0.24	0.42	0.65
2+ doctor visits due to injury	-0.07	0.15	0.97	0.01	0.32	0.89	0.11	0.23	0.91
Any ER/Urgent Care Visit	0.94	-0.08	0.17	0.95	-0.07	0.15	0.95	-0.06	0.15
Number of ER/Urgent Care Visits	0.87	0.12	0.13	0.89	0.09	0.12	0.91	0.07	0.11
Eigenvalue	1.94	0.35		1.97	0.44		2.10	0.40	

Note. +p<0.10, *p<0.05. Exploratory factor analysis was conducted with oblique oblimin rotation

© 2023 Sperber JF et al. *JAMA Network Open.*

eTable 5. Findings From Current Published Papers, Working Papers, and Preprints Reporting BFY Impacts

**Age-1 Wave: Brain activity (electroencephalography)**	
Troller-Renfree et al., 2022, *PNAS*	Infants in the high-cash gift group demonstrated statistically significantly higher brain activity than infants in the low-cash gift group in the beta and gamma frequency bands, as well as in a composite of mid-to-high-frequency power . No statistically significant differences were detected in the low-frequency (theta) band.
**Age-1 Wave: Substance Use and Alcohol and Cigarette Expenditures (maternal reports)**	
Yoo et al., 2022, *BMC Public Health*	No statistically significant treatment impacts were detected on substance use or expenditures on alcohol or cigarettes.
**Age-1 Wave: Financial and Time Investments in Child-related Goods and Activities (maternal reports and transaction data)**	
Gennetian et al., 2022, *NBER Working Paper*	Families in the high-cash gift group spent more money on child-specific goods and spent more time engaging in early-learning activities than families in the low-cash gift group. No statistically significant treatment impacts were detected on general household expenditures, maternal labor supply, infants' time in childcare, various metrics of economic hardship, social services receipt, or mothers' subjective wellbeing.
**Age-1 Wave: Family Stress and Stress-Related Processes (maternal reports)**	
Magnuson et al., 2022, *SSRN Preprint*	Families in the high-cash gift group reported statistically significantly higher household income, engagement in child activities, and maternal anxiety than families in the control group. No statistically significant treatment impacts were detected for feelings of economic pressure, psychological distress, interparental relationship quality, or quality of play with infants.
**Age-1, -2, and -3 Waves: Maternal Employment (maternal reports)**	
Sauval et al., 2022, *SSRN Preprint*	No statistically significant treatment impacts were detected for maternal workforce participation, hours of paid work, or household earned income.

Notes. This table reports on the primary findings from current published papers, working papers, and preprint reporting BFY treatment impacts. The reported findings are a snapshot of each paper's take-aways. Readers are encouraged to refer to the original papers for more detail and nuance on the magnitude and robustness of all findings.

© 2023 Sperber JF et al. *JAMA Network Open.*

eFigure 1. Flow Diagram for Baseline, Age-1, Age-2, and Age-3 Data Collection[a]

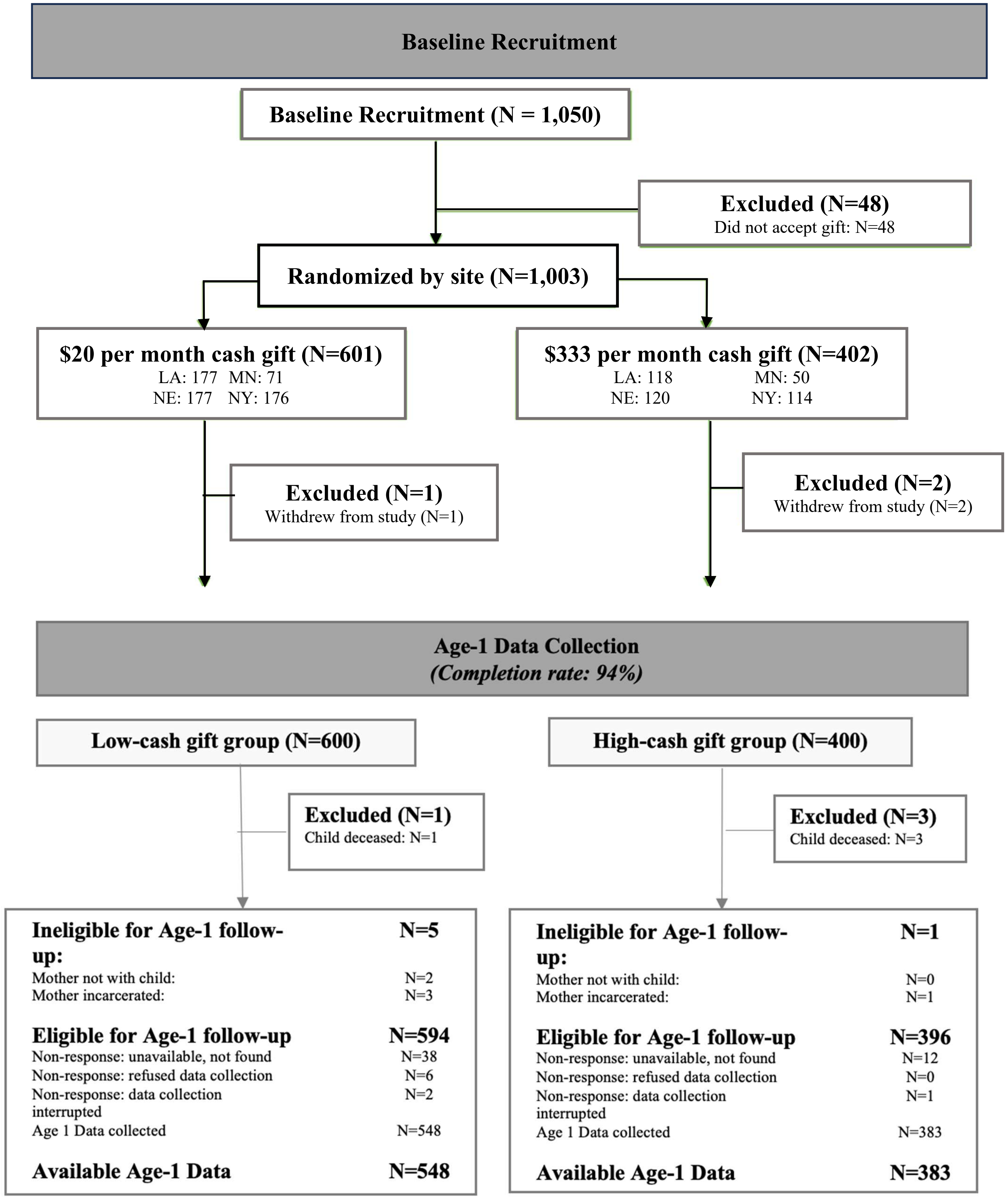

© 2023 Sperber JF et al. *JAMA Network Open.*

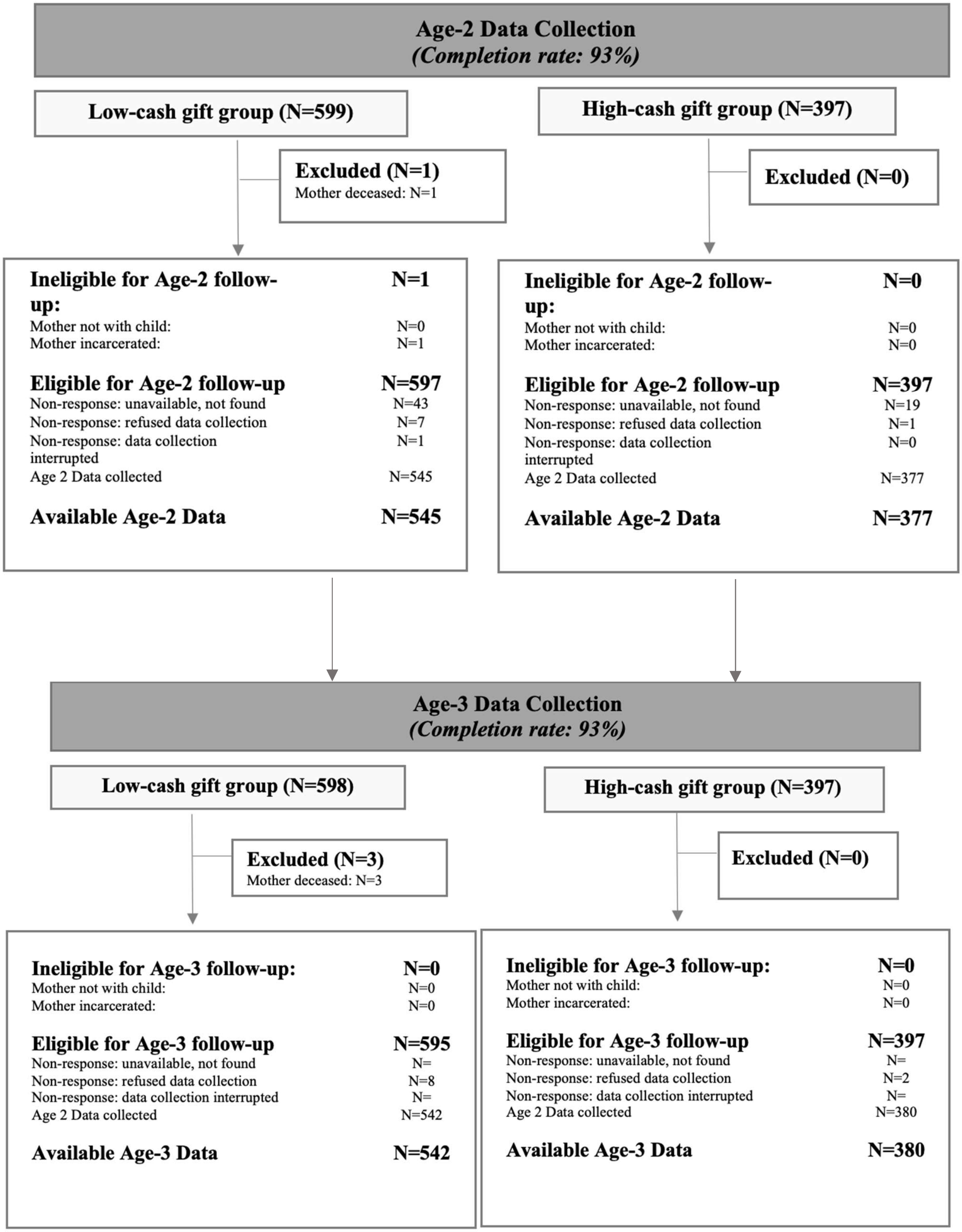

Note: [a] Baseline CONSORT diagram adapted from Noble et al. 2021. Ages 1 – 3 CONSORT diagrams adapted from Savaul et al. 2022

© 2023 Sperber JF et al. *JAMA Network Open.*
