# Supplementary material for: Unconditional Cash Transfers and Maternal Assessments of Children's Health, Nutrition, and Sleep: A Randomized Clinical Trial
Source: JAMA Netw Open. 2023 Sep 29;6(9):e2335237. doi: 10.1001/jamanetworkopen.2023.35237 (PMC10543132; doi:10.1001/jamanetworkopen.2023.35237)
Supplement: Supplement 3. — Data Sharing Statement [file jamanetwopen-e2335237-s003.pdf]

Data Sharing Statement

Sperber. Unconditional Cash Transfers and Maternal Assessments of Children's Health, Nutrition, and Sleep. *JAMA Netw Open*. Published September 22, 2023. doi:10.1001/jamanetworkopen.2023.35237

Data
Data available: Yes
Data types: Deidentified participant data
How to access data: https://www.icpsr.umich.edu/web/DSDR/studies/37871/versions/V3
When available: With publication

Supporting Documents
Document types: None

Additional Information
Who can access the data: Data is publicly available. Analytic code will be provided upon request
Types of analyses: Data is publicly available
Mechanisms of data availability: Data is publicly available
